# Supplementary material for: ReSeq simulates realistic Illumina high-throughput sequencing data
Source: Genome Biol. 2021 Feb 19;22:67. doi: 10.1186/s13059-021-02265-7 (PMC7896392; doi:10.1186/s13059-021-02265-7)
Supplement: Supplementary file 1 — Additional file 1 Supplementary figures, tables and formulas. [file 13059_2021_2265_MOESM1_ESM.pdf]

Supplementary: ReSeq simulates realistic Illumina high-throughput sequencing data

Supplementary Figures

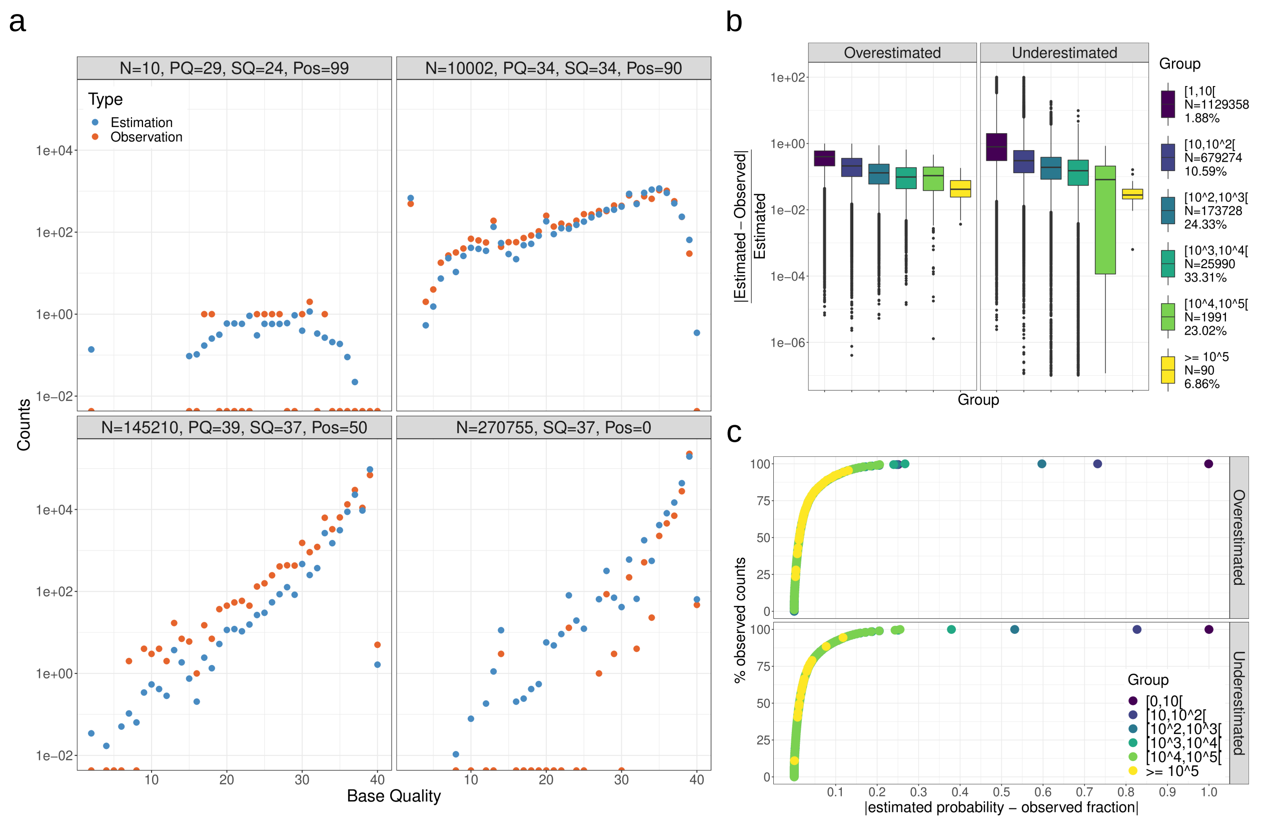

Fig. S1 Accuracy of two-dimensional estimation of quality matrix for Ec-Hi2000-TruSeq. All values are only based on the matrix part for the first read, nucleotide A and error rate 0. a) Sample slices of the matrix comparing the estimation to the observed counts for different values of previous quality, sequence quality and position. N specifies the total number of observed counts in this slice. The two lower slices with high total number of counts show consistent, moderate over- or underestimation, while the top left slice with only 10 total counts shows how the estimation deals well with low numbers of observed counts. b) Relative difference between observation and estimation grouped by number of observed counts. The plot lists for each group the number of compared values N and the percentage of counts from the matrix. Deviations from low counts are also caused by the integer nature of counts and not necessarily an estimation error. More than 75% of the values with at least 100 observed counts deviate by less than 30% from the observation. c) The maximum absolute deviation for the given percent of all observed counts. The deviation is normalized by the total number of counts in each slice of previous quality, sequence quality and position. The deviations falling into each percentage bin are grouped based on their observed counts. For more than 80% of the data the deviation is below 0.05.

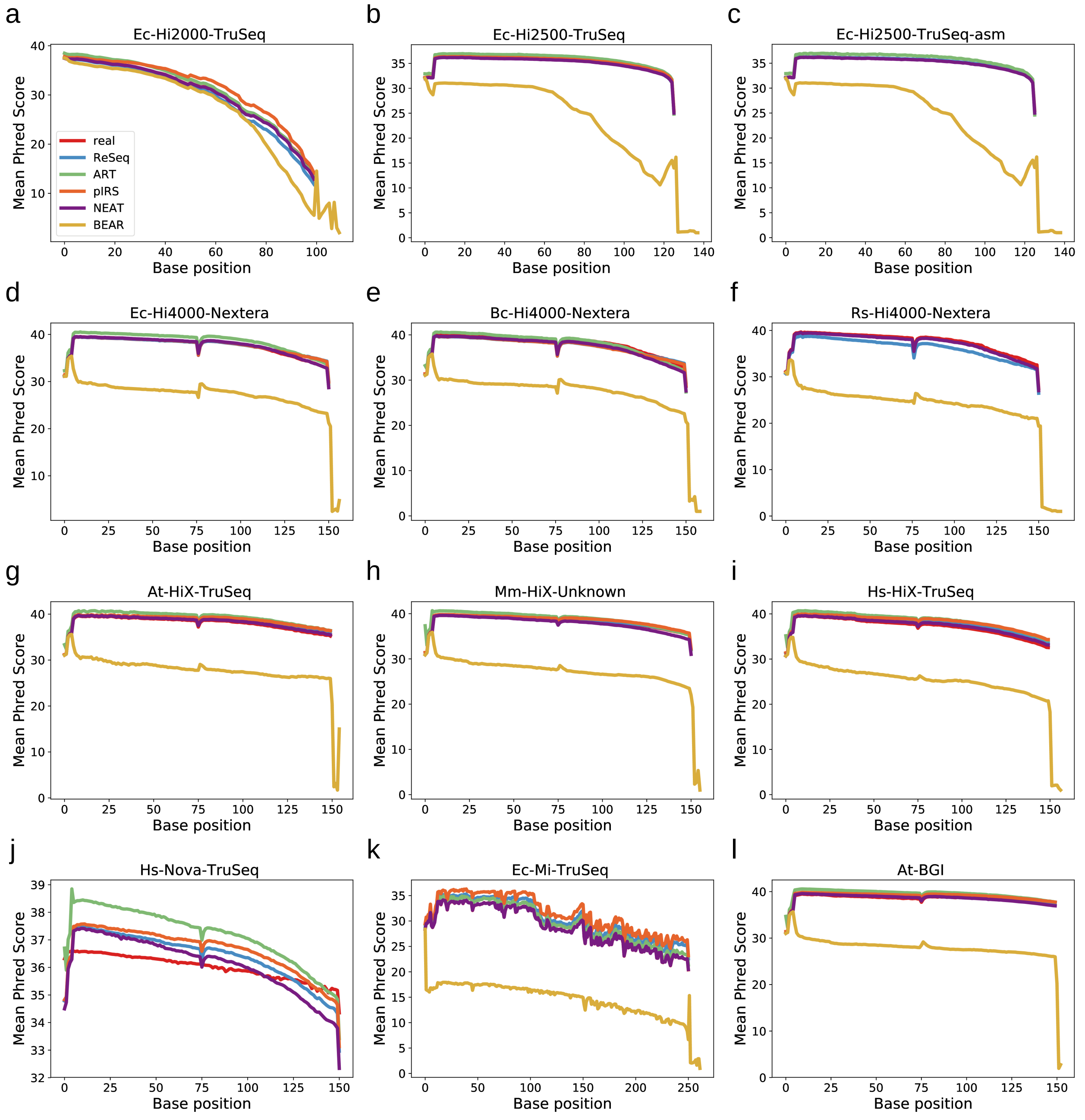

Fig. S2 Mean quality values by position for real and simulated data. Note that: c) preqc crashed for pIRS f) preqc crashed for pIRS and ART j) BEAR was omitted due to excessive runtimes

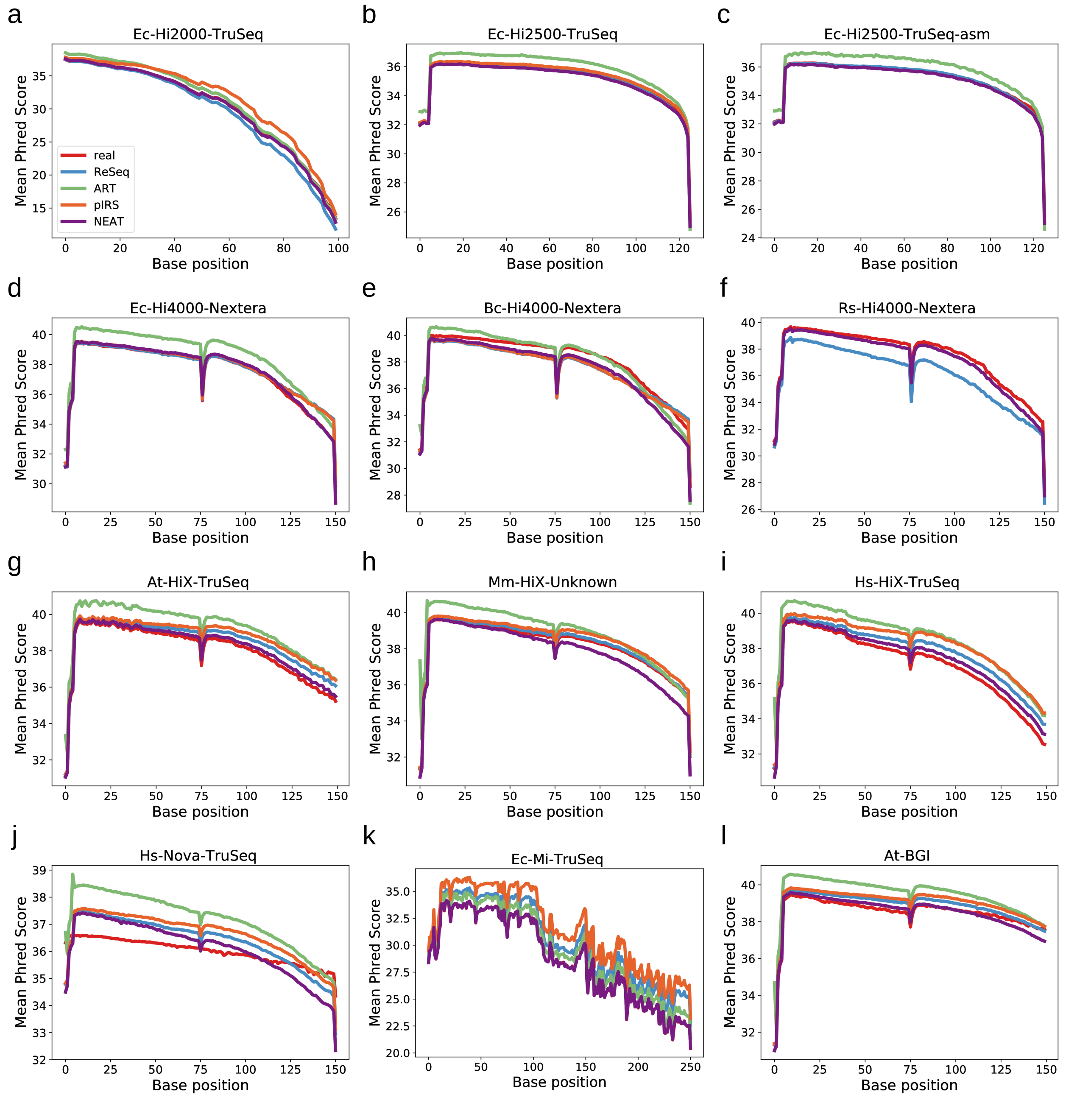

Fig. S3 Mean quality values by position for real and simulated data without BEAR. Note that: c) preqc crashed for pIRS f) preqc crashed for pIRS and ART

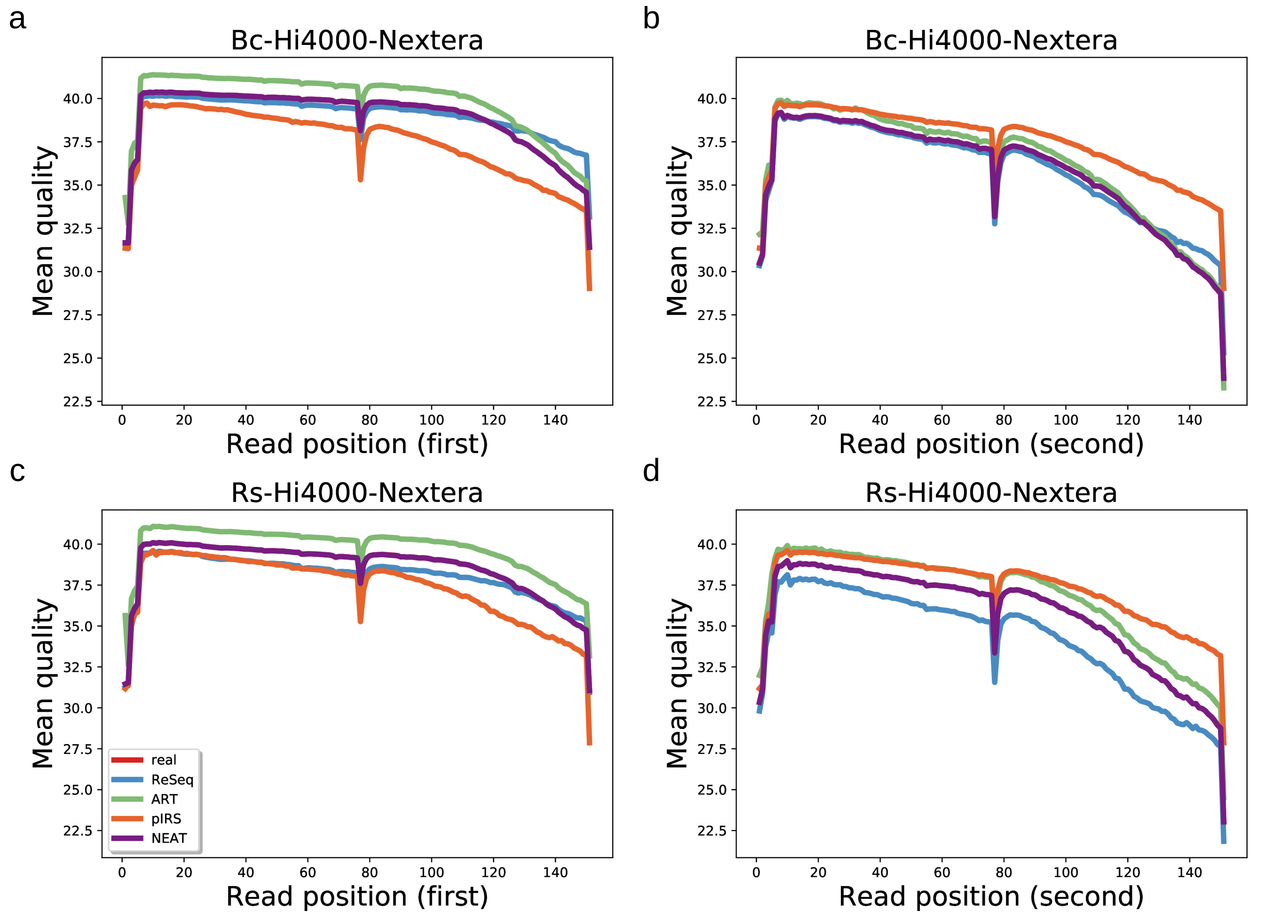

Fig. S4 Mean quality values by position for real and simulated data, separately for first and second read. NEAT follows the real data perfectly and covers the real data line. This is slightly different from Figure S3, where preqc filters some reads. BEAR is excluded from this plot to achieve better resolution for the other simulators.

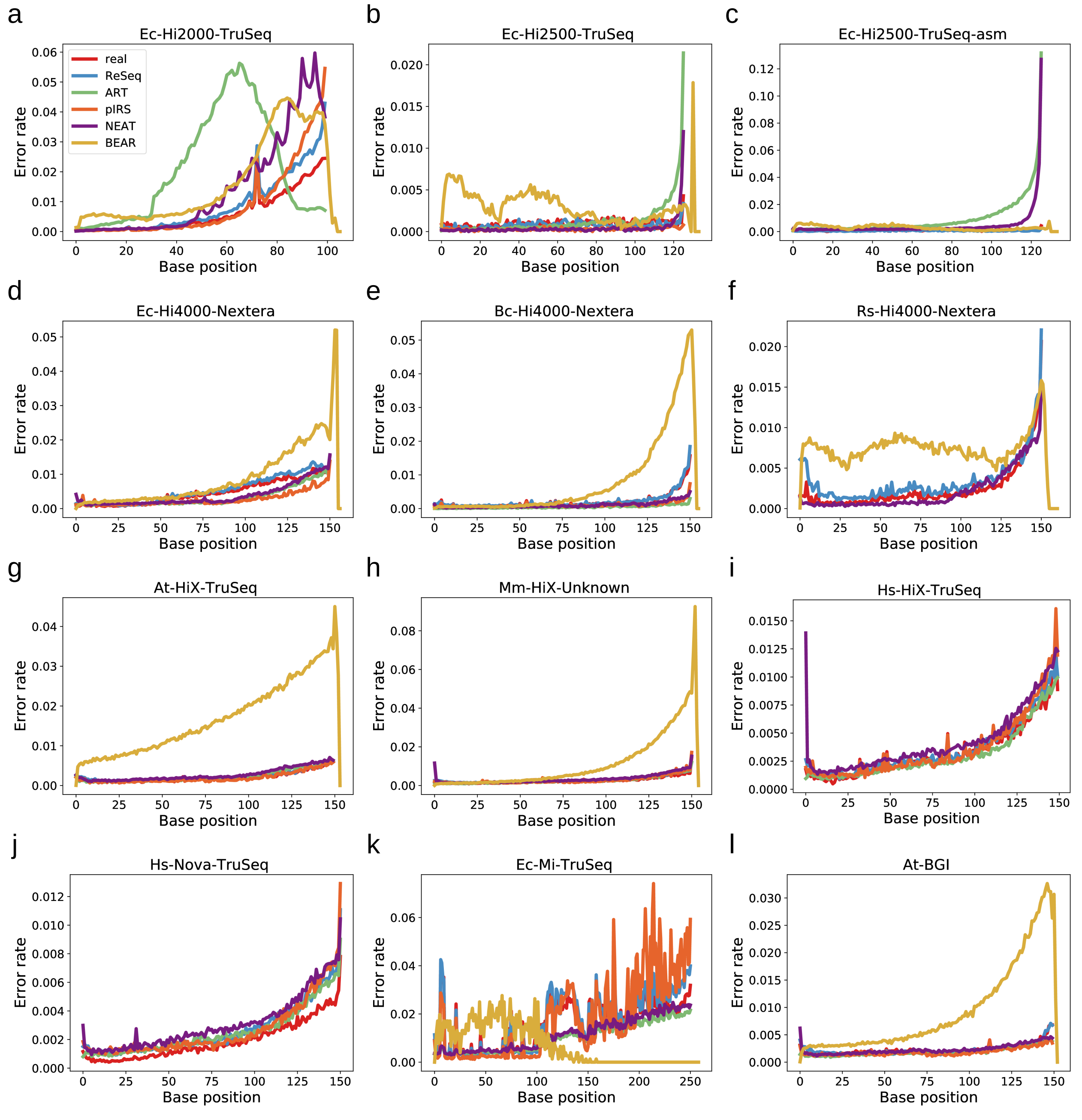

Fig. S5 Mean error rates by position for real and simulated data. Note that: c) preqc crashed for pIRS f) preqc crashed for pIRS and ART i) BEAR was excluded due to sporadic reports of error rates up to 1 for positions up to 700. ȷ) BEAR was omitted due to excessive runtimes

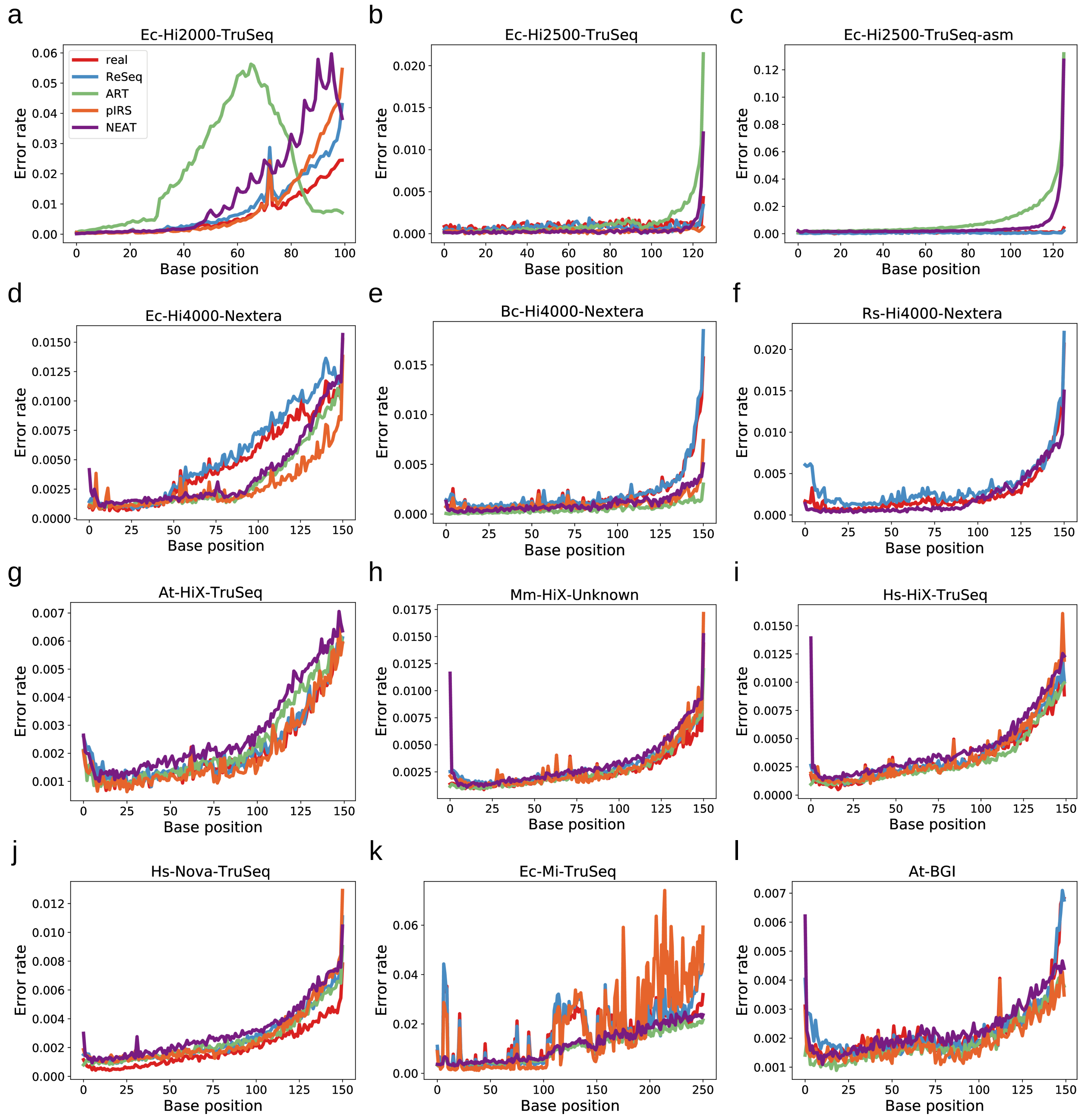

Fig. S6 Mean error rates by position for real and simulated data without BEAR. Note that: c) preqc crashed for pIRS f) preqc crashed for pIRS and ART

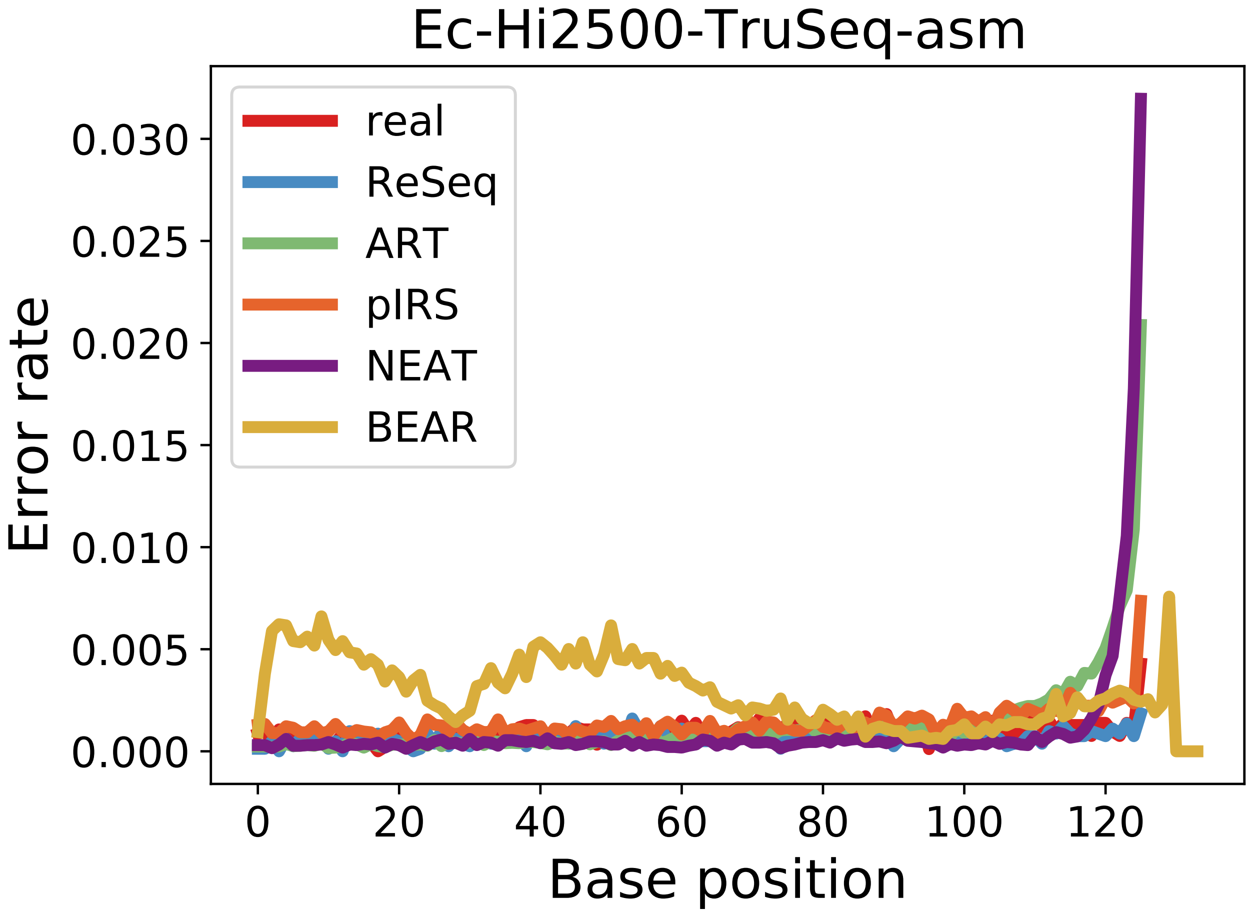

Fig. S7 Mean error rates by position for real and simulated data for Ec-Hi2500-TruSeq-asm with the median coverage from Ec-Hi2500-TruSeq provided to all simulators except BEAR.

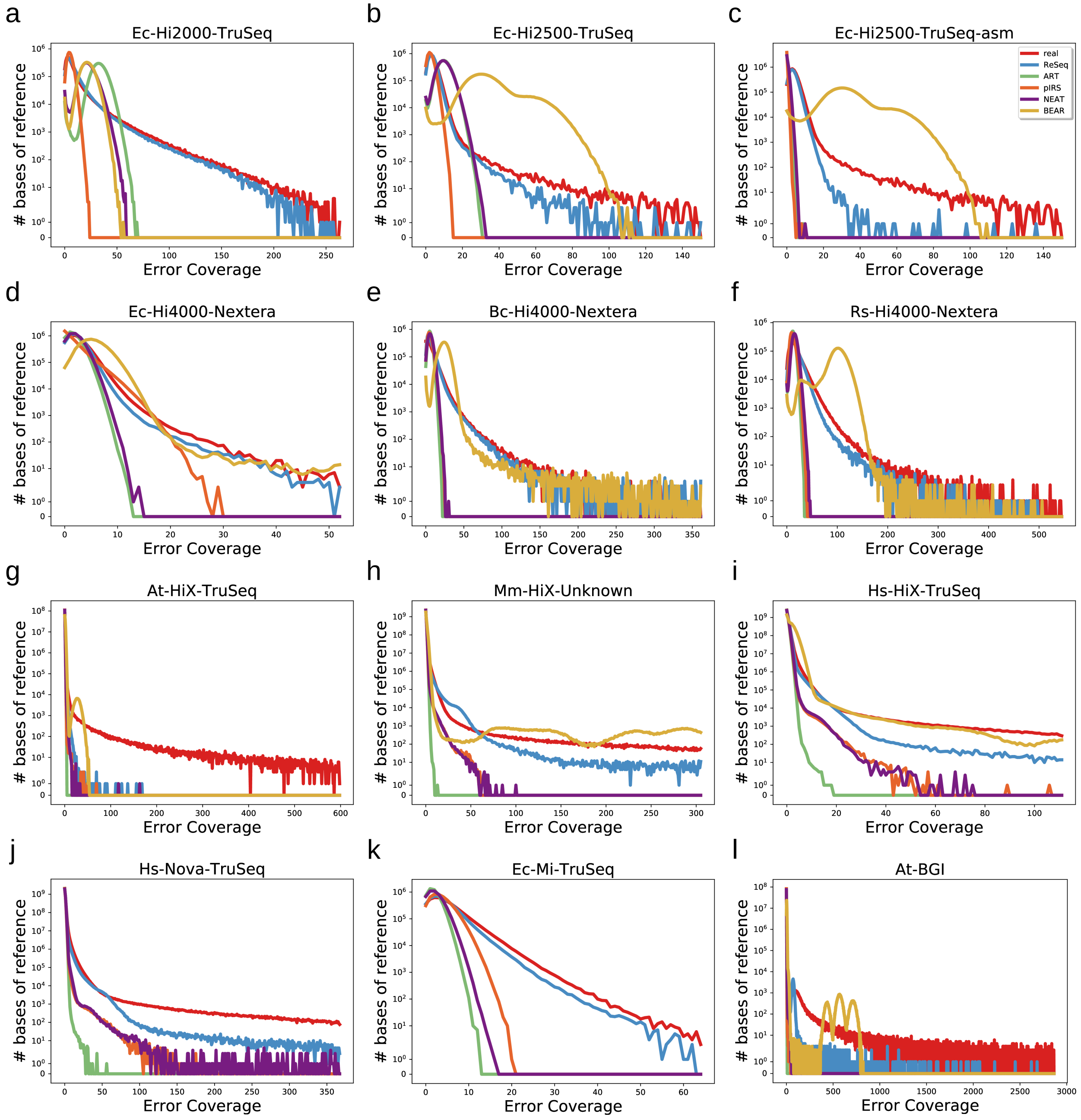

Fig. S8 Systematic error distribution in the genome. Note that: j) BEAR was omitted due to excessive runtimes. k) BEAR was omitted, because it did not have a single proper pair mapped.

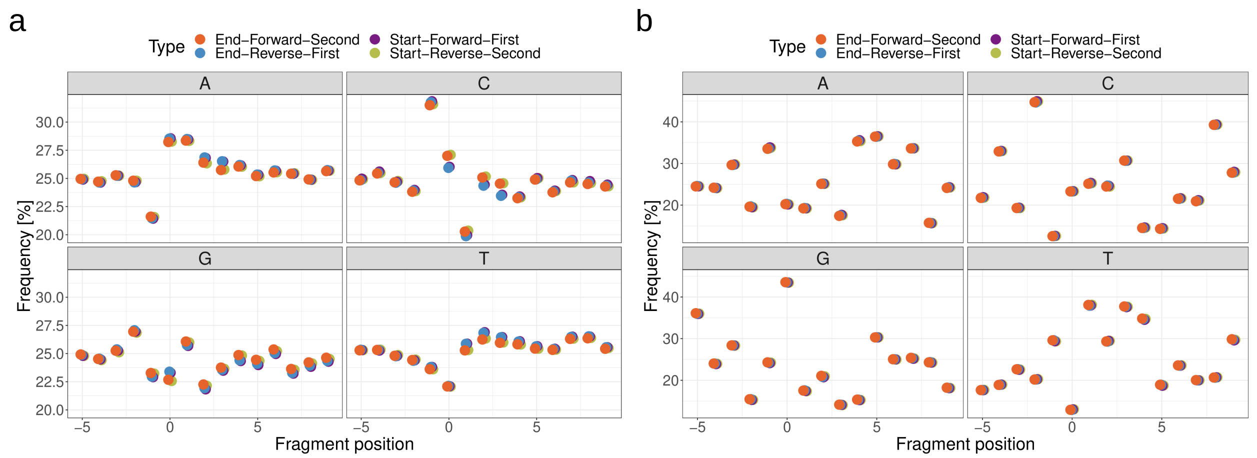

Fig. S9 Observed frequency of nucleotides in the fragments. The position is relative to the fragment start/end. Positive positions and zero are in the fragment. Negative positions are outside of the fragment. The flanking bias is less pronounced in the mechanically-fragmented Ec-Hi2000-TruSeq (a) compared to Ec-Hi4000-Nextera (b), where enzymes have been used. The colour specifies, whether the flanking sequences of the start or end of the fragment are shown, if the fragment is on the forward or reverse strand and if the first or second read maps on this side of the fragment. A small deviation between first and second reads is visible (a), which is not included in Reseq's coverage model.

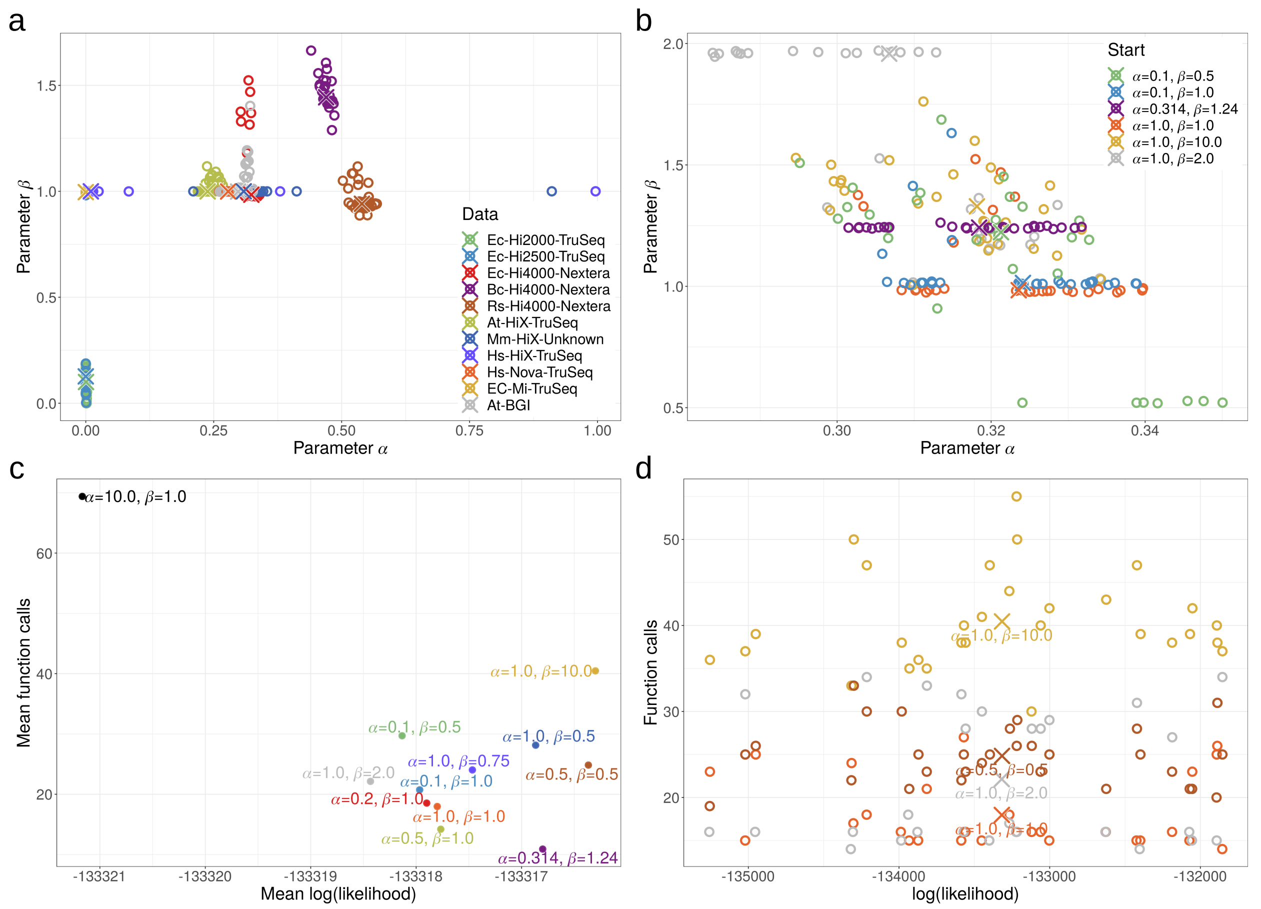

Fig. S10 Dispersion parameter overview. a) Comparison of the resulting parameters over 11 tested datasets. Each circle is a fit. Crosses represent the median (final result) for each dataset. Ec-Hi2000-TruSeq and Ec-Hi2500-TruSeq seem to suppress parameter α, while all other datasets require it. A likely explanation are patterned flow cells, which are build into newer Illumina sequencers, but not into the HiSeq2000/2500 used for those two datasets. Strong increases of optical duplicates for patterned flow cells have been reported before [1]. That parameter β is fixed to its start value of 1 is a precision artifact (panel b) mostly observed for low-coverage datasets. b) Converged parameters for Ec-Hi4000-Nextera depending on the start parameters. Circles are single fits and crosses medians for each set of start parameters. If parameter β starts too close to the optimal value, it remains in proximity of its starting value. Changing the default start parameters is not a good solution (panel c). c) Final log(likelihood) vs. total number of calls to the likelihood calculation for Ec-Hi4000-Nextera. Each dot represents the mean over all fits. Start values further away from the optimal value are not guaranteed to increase the likelihood and often need more function calls. d) Final log(likelihood) vs. total number of calls to the likelihood calculation for Ec-Hi4000-Nextera. Each circle is a single fit and the crosses are the mean values shown in (c). The mean shift of log(likelihood) caused by different start parameters is minor compared to the spread between individual fits.

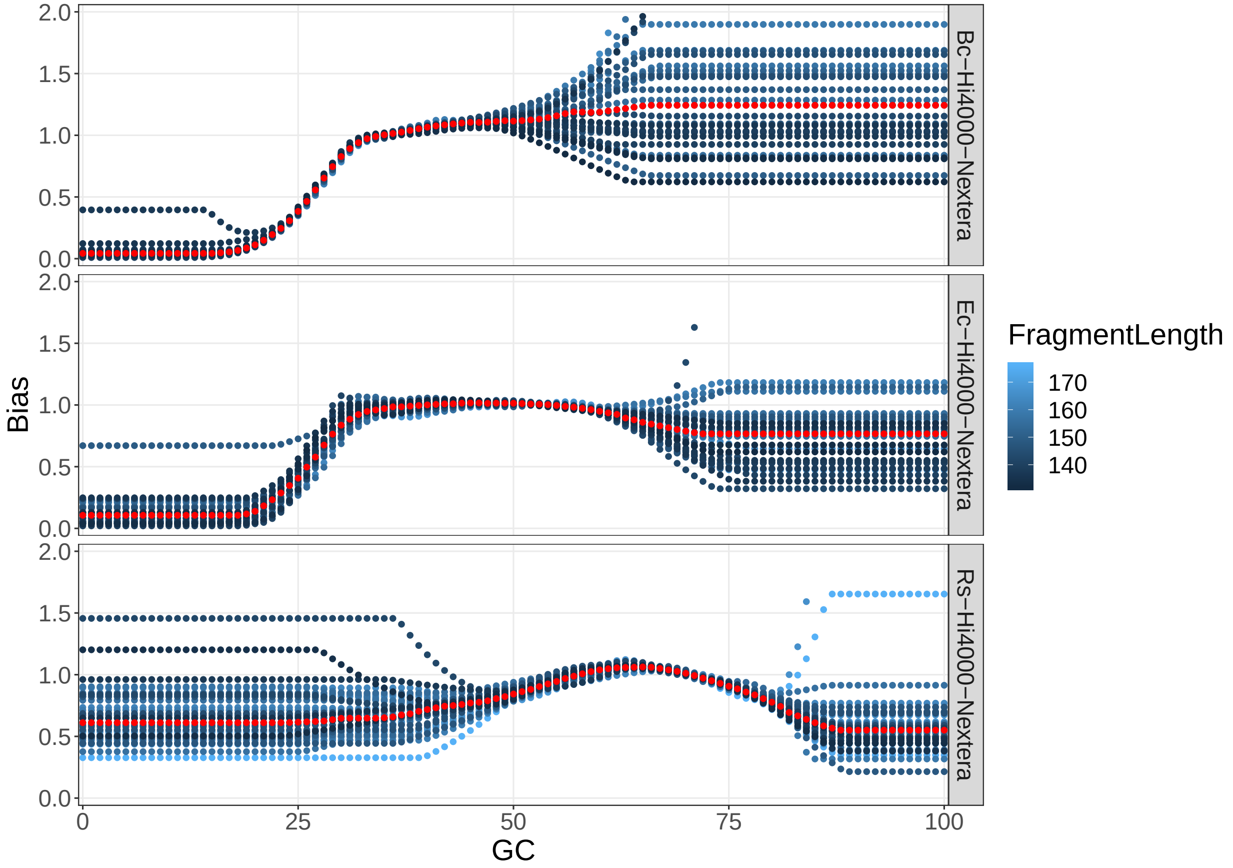

Fig. S11 Comparison of GC bias for three dataset created with Nextera adapters.

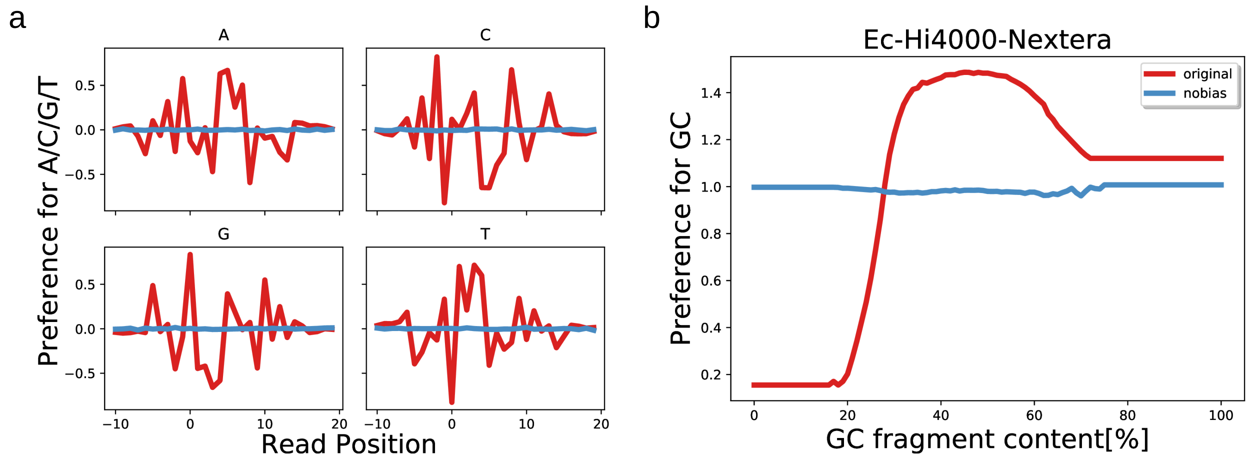

Fig. S12 Estimated bias for simulated, uniformly distributed data based on Ec-Hi4000-Nextera. The bias estimated for the original dataset is given as a reference for the magnitude of the biases. a) Flanking bias b) GC bias

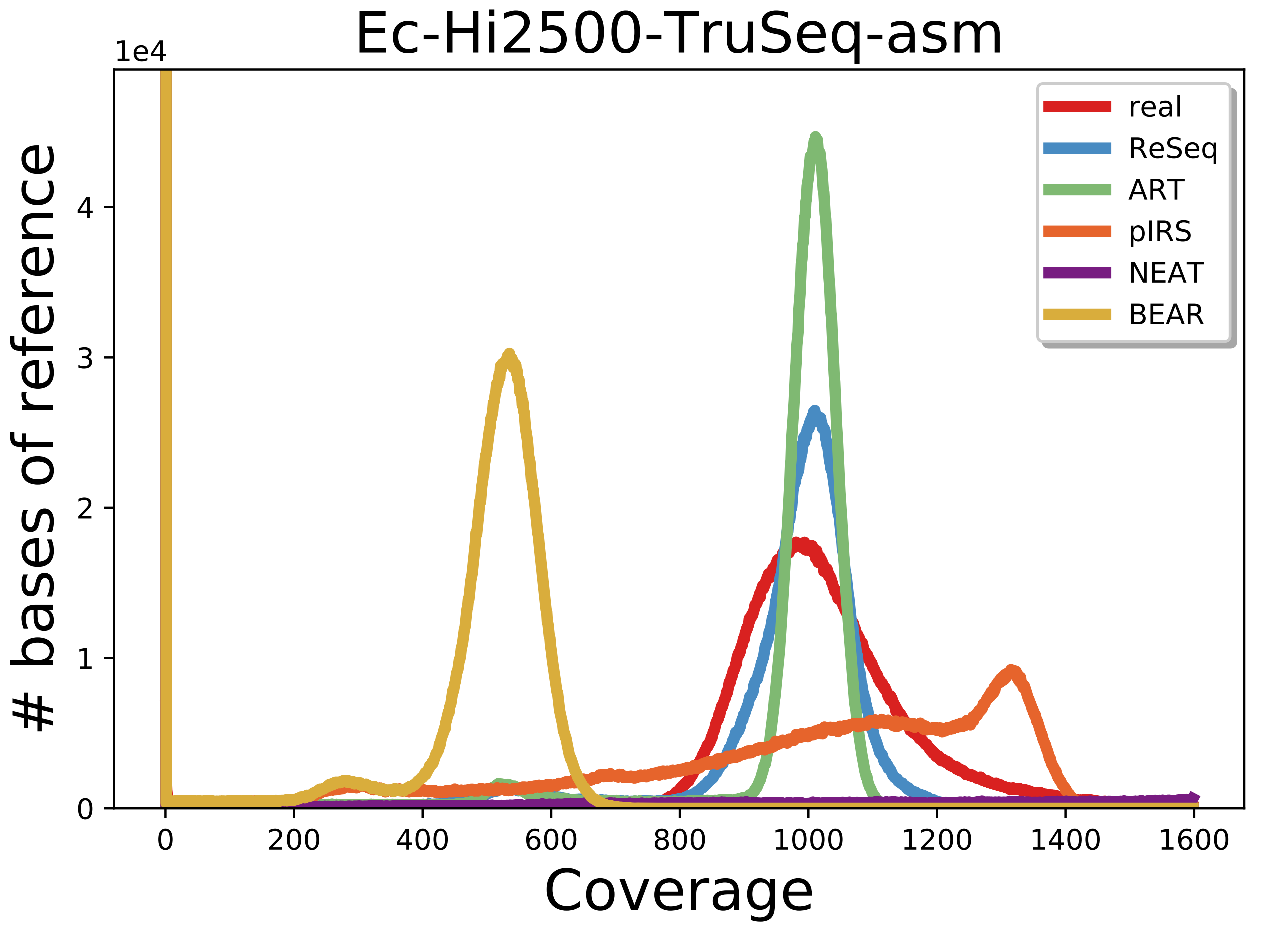

Fig. S13 Base-coverage distribution of real and simulated data for Ec-Hi2500-TruSeq-asm with the median coverage from Ec-Hi2500-TruSeq provided to all simulators except BEAR.

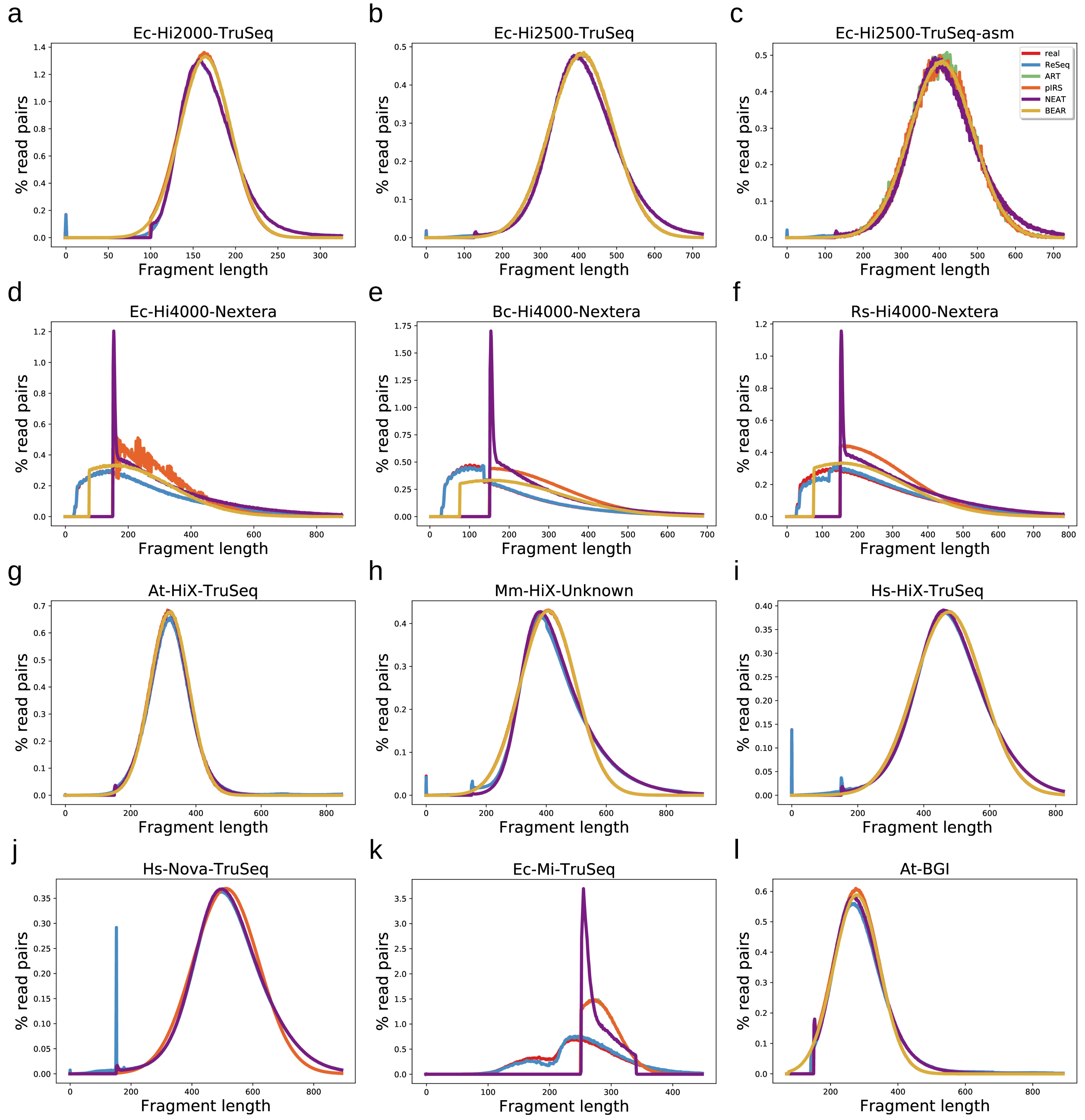

Fig. S14 Fragment length distribution for real and simulated data. Note that: j) BEAR was omitted due to excessive runtimes. k) BEAR was omitted, because it did not have a single proper pair mapped.

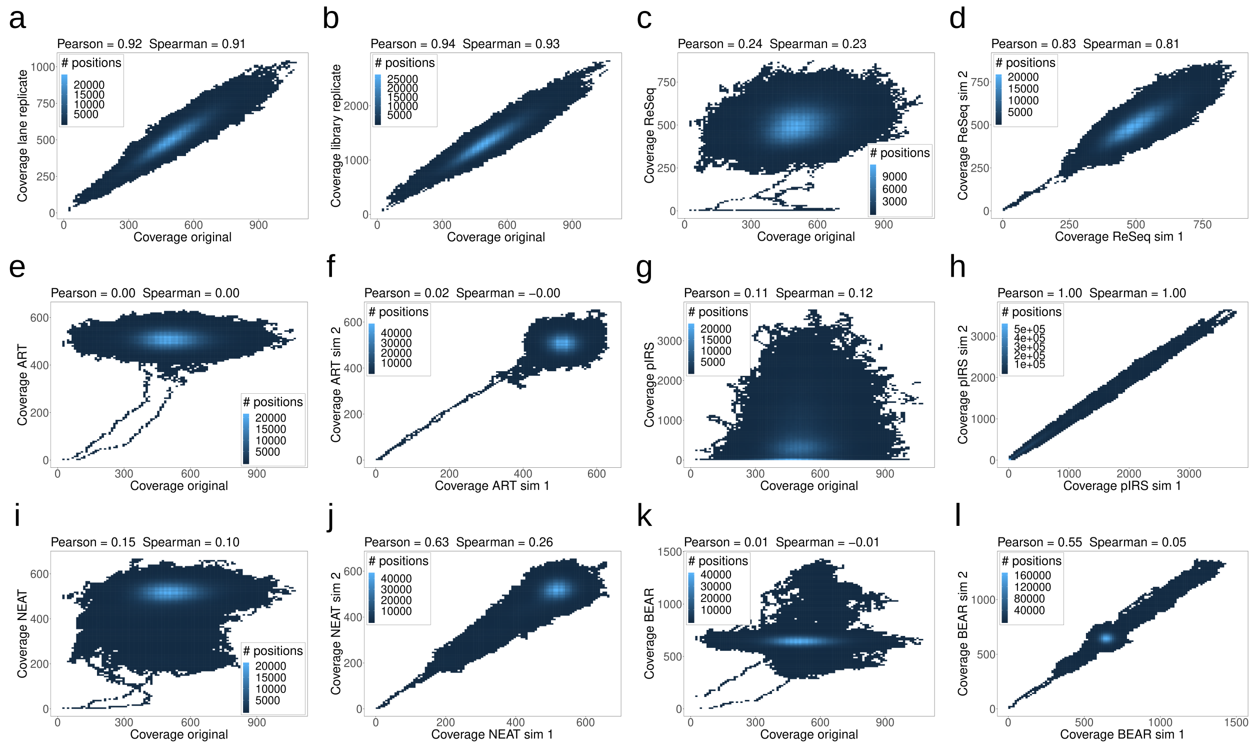

Fig. S15 Correlations of real and simulated coverages across datasets. a) Ec-Hi4000-Nextera (Ecoli1_L001) vs Ecoli1_L002 b) Ec-Hi4000-Nextera (Ecoli1_L001) vs. Ecoli2_L001 c) Ec-Hi4000-Nextera vs. ReSeq d) ReSeq e) Ec-Hi4000-Nextera vs. ART f) ART g) Ec-Hi4000-Nextera vs. pIRS h) pIRS i) Ec-Hi4000-Nextera vs. NEAT j) NEAT k) Ec-Hi4000-Nextera vs. BEAR l) BEAR

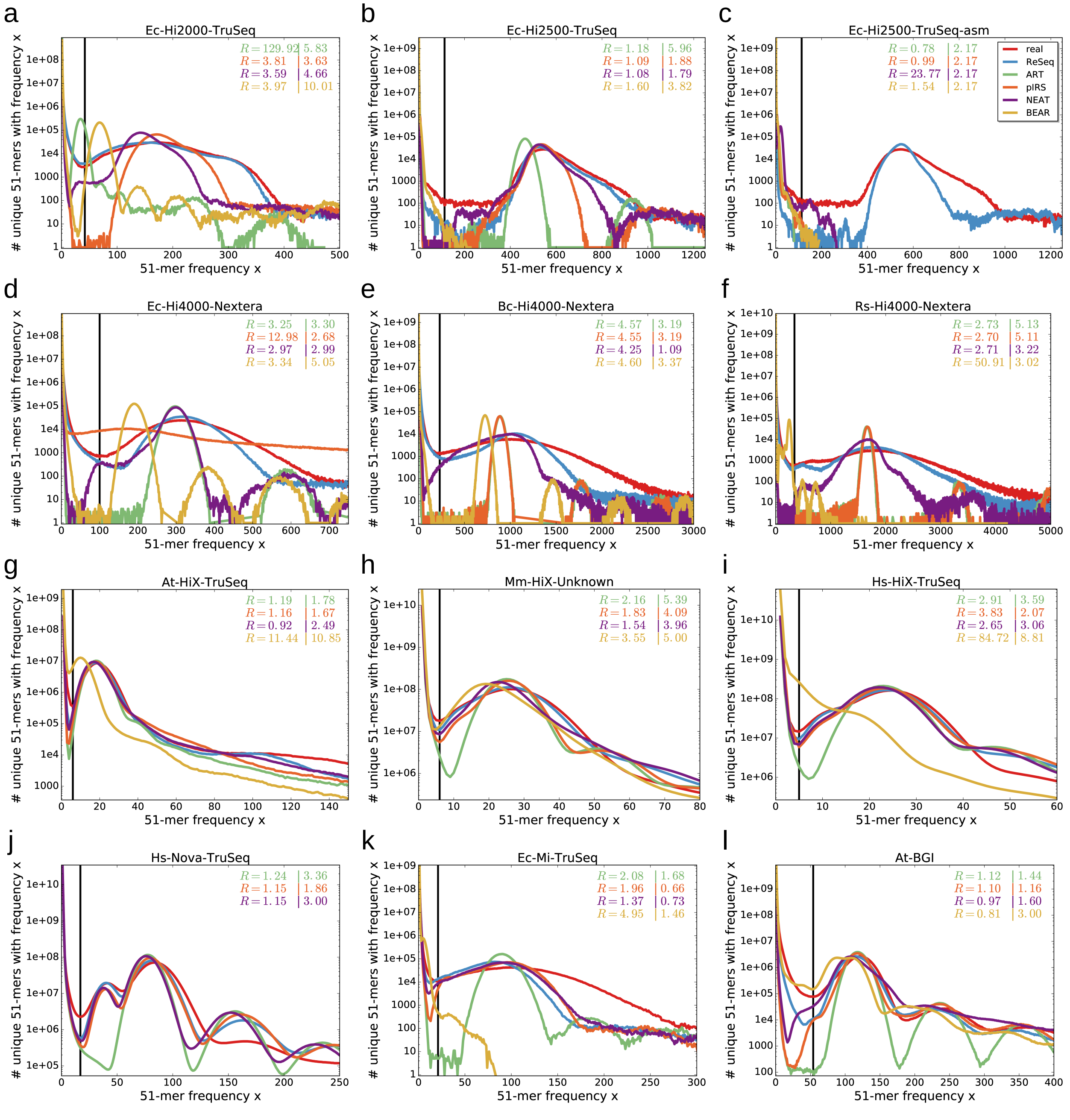

Fig. S16 51-mer spectra of real and simulated data. The shape and position of peaks reflects the coverage distribution, while the exponential decrease at low frequencies is defined by systematic errors. The black lines show the minimum between the exponential decrease and signal peak for real data. The first value of R gives the sum of relative deviations for frequencies (x) up to the minimum. The second value of R gives the sum of absolute deviations for frequencies larger than the minimum. Both values are stated relative to ReSeq. When the signal peak of the simulation does not exist or starts before the minimum in real data, the values lose their interpretability, which happens especially for ART (a,c), pIRS (c,d,l), NEAT (a,c-e,l) and BEAR (b-c,f-g,i,k-l).

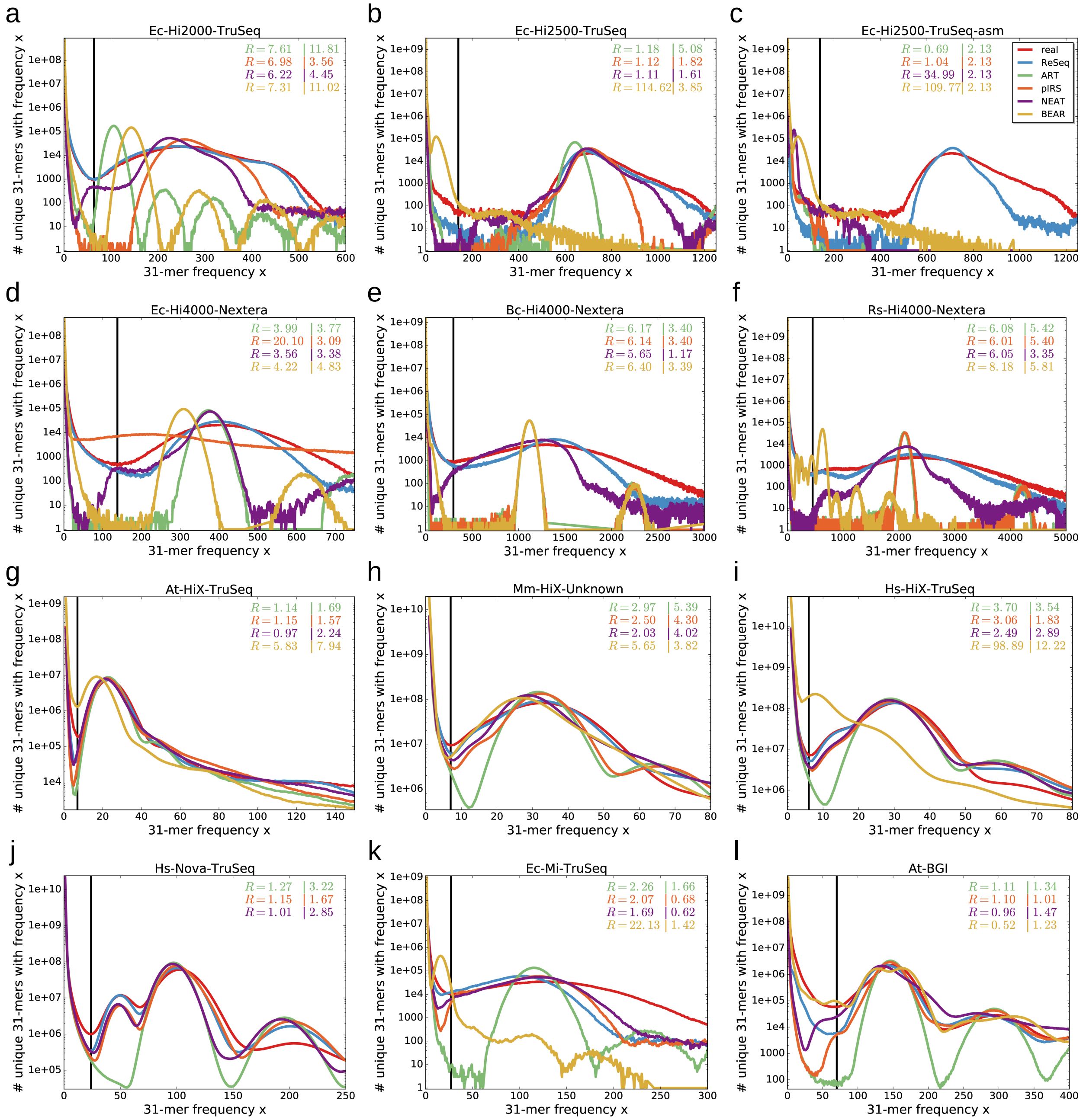

Fig. S17 31-mer spectra of real and simulated data. The shape and position of peaks reflects the coverage distribution, while the exponential decrease at low frequencies is defined by systematic errors. The black lines show the minimum between the exponential decrease and signal peak for real data. The first value of R gives the sum of relative deviations for frequencies (x) up to the minimum. The second value of R gives the sum of absolute deviations for frequencies larger than the minimum. Both values are stated relative to ReSeq. When the signal peak of the simulation does not exist or starts before the minimum in real data, the values lose their interpretability, which happens especially for ART (c), pIRS (c,d,l), NEAT (c,l) and BEAR (b-c,f,i,k-l).

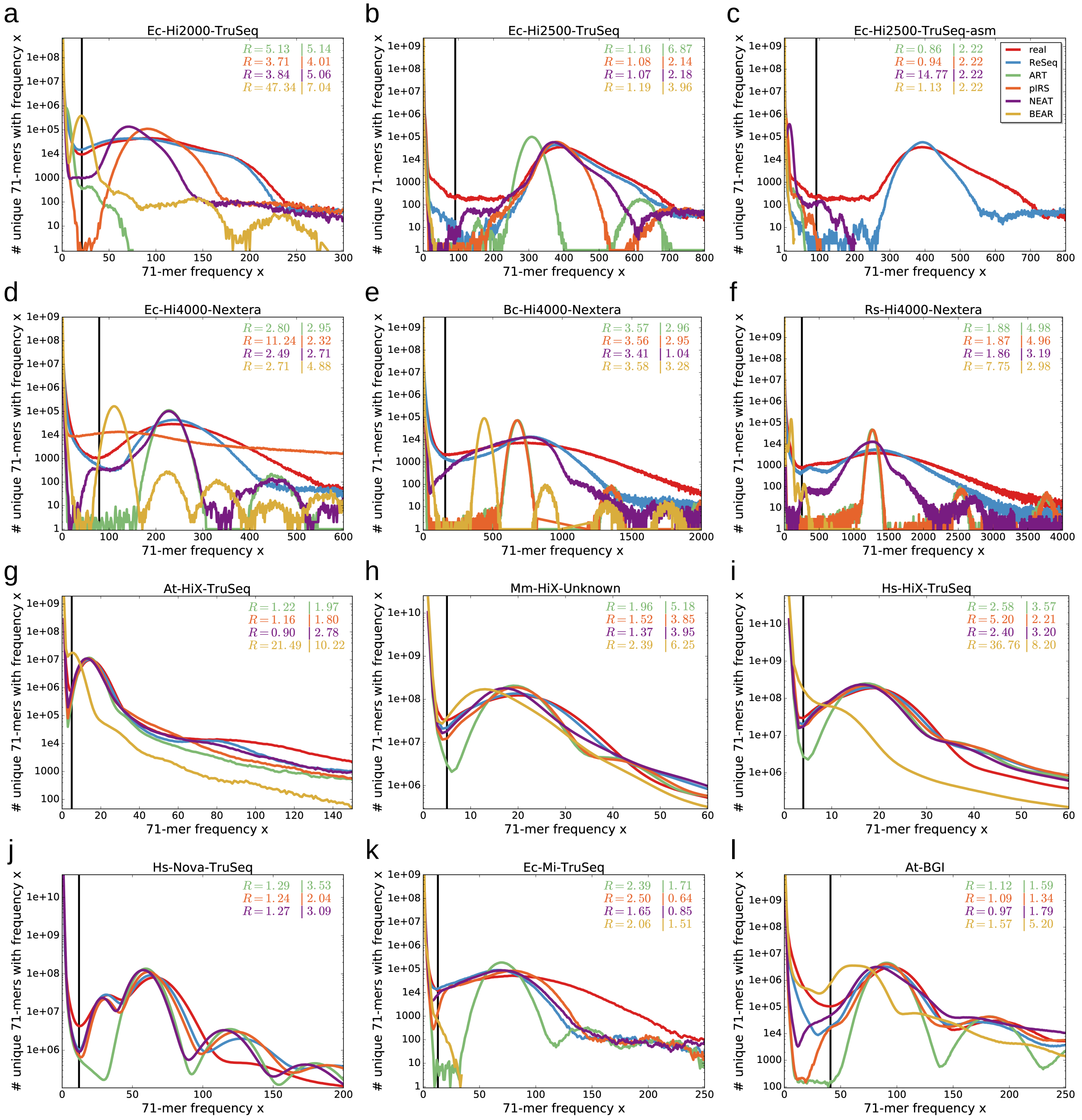

Fig. S18 71-mer spectra of real and simulated data. The shape and position of peaks reflects the coverage distribution, while the exponential decrease at low frequencies is defined by systematic errors. The black lines show the minimum between the exponential decrease and signal peak for real data. The first value of R gives the sum of relative deviations for frequencies (x) up to the minimum. The second value of R gives the sum of absolute deviations for frequencies larger than the minimum. Both values are stated relative to ReSeq. When the signal peak of the simulation does not exist or starts before the minimum in real data, the values lose their interpretability, which happens especially for ART (a,c), pIRS (c,d,l), NEAT (a,c-e,l) and BEAR (b-c,f-g,i,k-l).

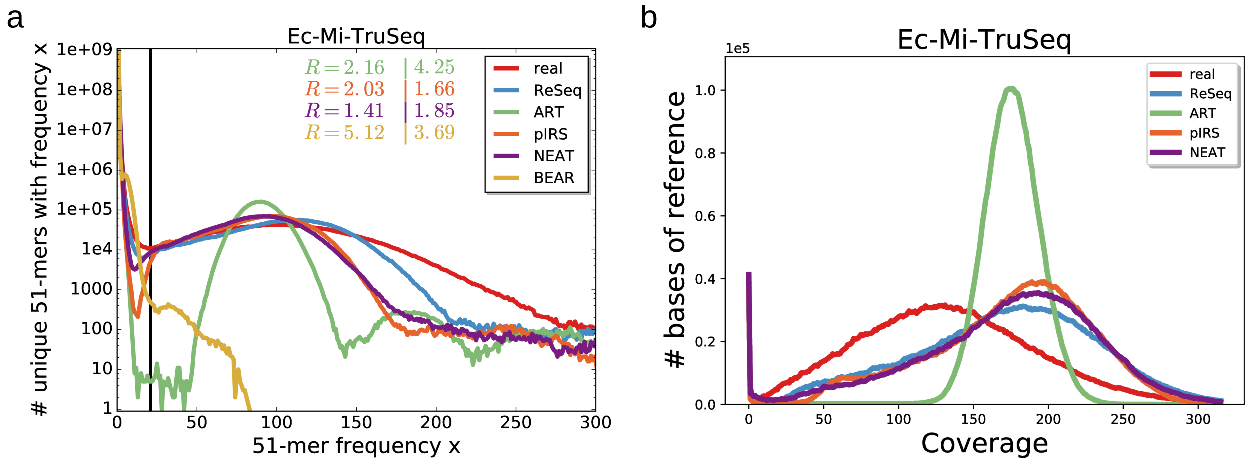

Fig. S19 51-mer spectra and base-coverage distribution of real and simulated data for Ec-Mi-TruSeq with the median coverage provided to all simulators except BEAR. a) 51-mer spectrum. The shape and position of peaks reflects the coverage distribution, while the exponential decrease at low frequencies is defined by systematic errors. The black lines show the minimum between the exponential decrease and signal peak for real data. The first value of R gives the sum of relative deviations for frequencies (x) up to the minimum. The second value of R gives the sum of absolute deviations for frequencies larger than the minimum. Both values are stated relative to ReSeq. When the signal peak of the simulation does not exist or starts before the minimum in real data, the values lose their interpretability, which happens for BEAR. b) Base-coverage distribution. BEAR was omitted, because it did not have a single proper pair mapped.

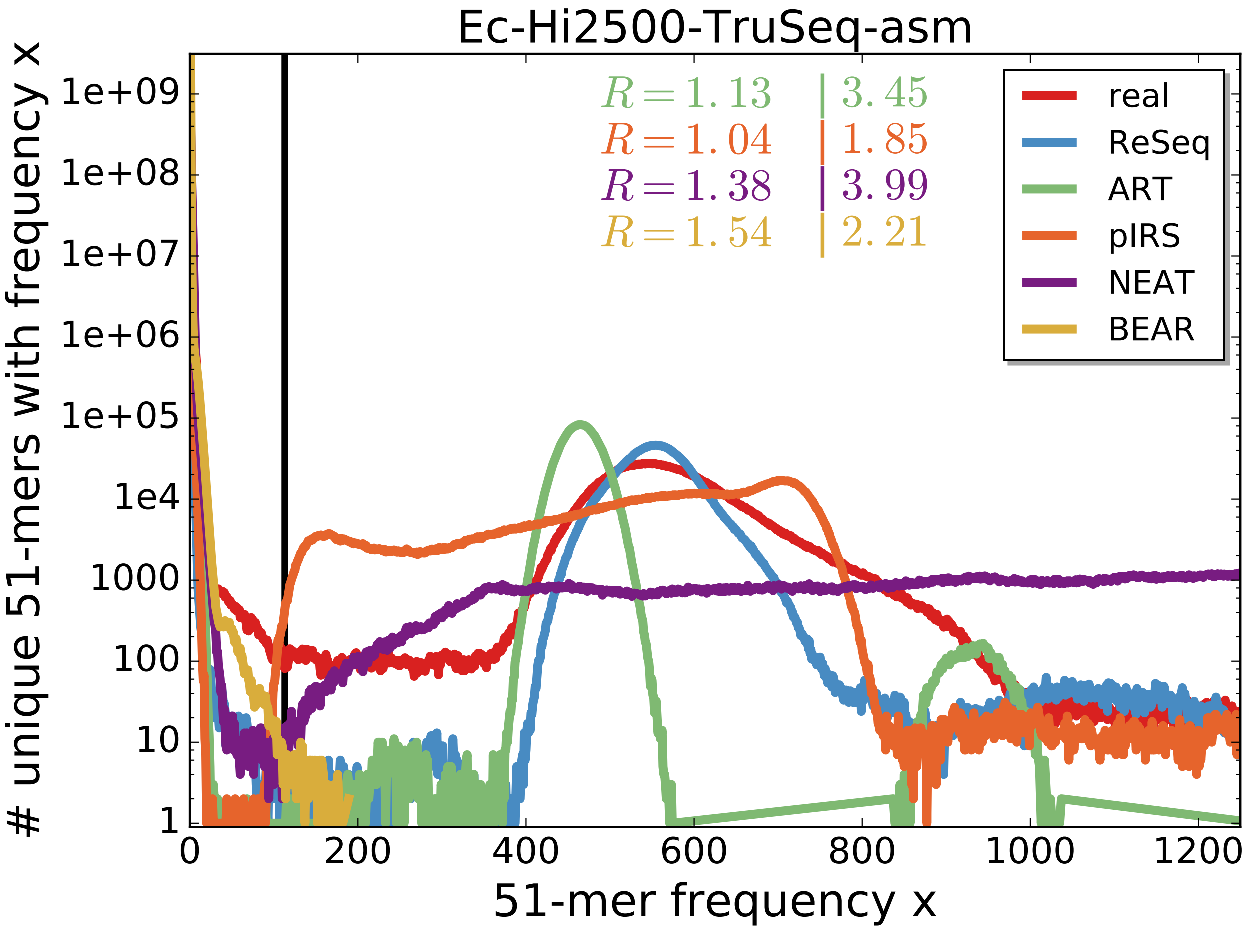

Fig. S20 51-mer spectra of real and simulated data for Ec-Hi2500-TruSeq-asm with the median coverage from Ec-Hi2500-TruSeq provided to all simulators except BEAR. The shape and position of peaks reflects the coverage distribution, while the exponential decrease at low frequencies is defined by systematic errors. The black lines show the minimum between the exponential decrease and signal peak for real data. The first value of R gives the sum of relative deviations for frequencies (x) up to the minimum. The second value of R gives the sum of absolute deviations for frequencies larger than the minimum. Both values are stated relative to ReSeq. When the signal peak of the simulation does not exist or starts before the minimum in real data, the values lose their interpretability, which happens for BEAR and to a smaller extent for pIRS.

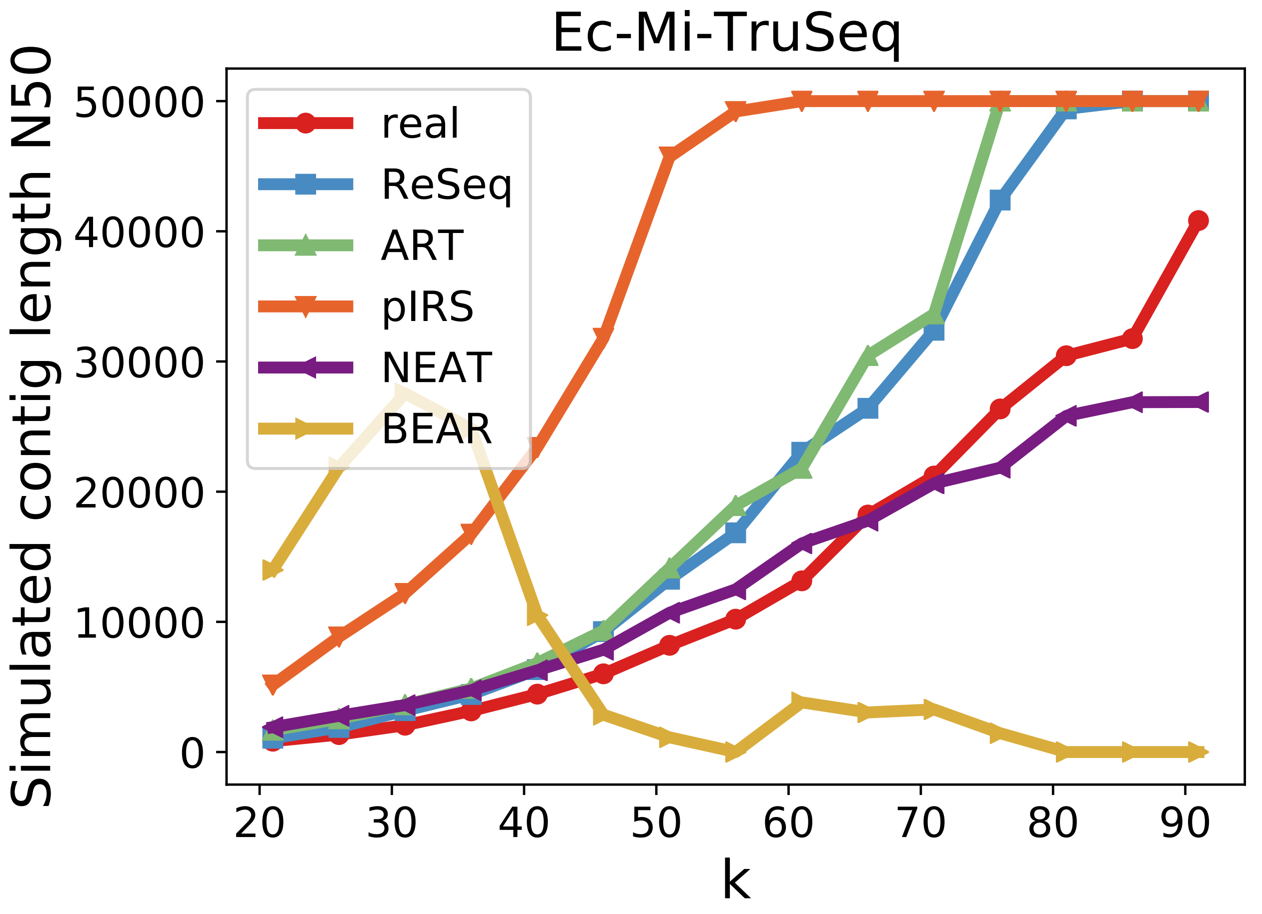

Fig. S21 Simulated contig N50 for real and simulated data for Ec-Mi-TruSeq with the median coverage provided to all simulators except BEAR.

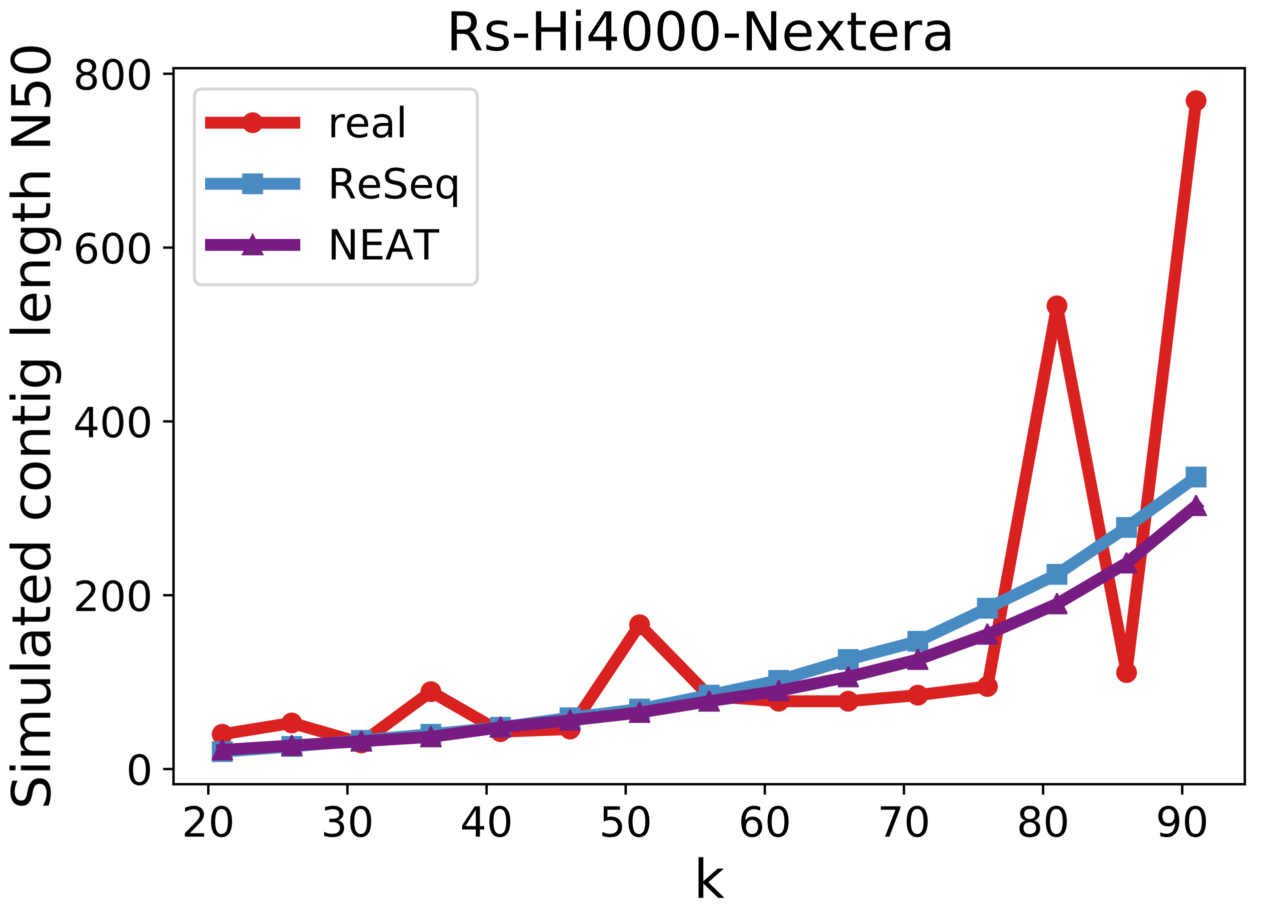

Fig. S22 Simulated contig N50 for real and simulated data for RS-Hi4000-Nextera with BEAR removed. Bear was removed due to its strong deviation from the real N50 values. pIRS and ART are also missing, because the preqc module crashed while processing them.

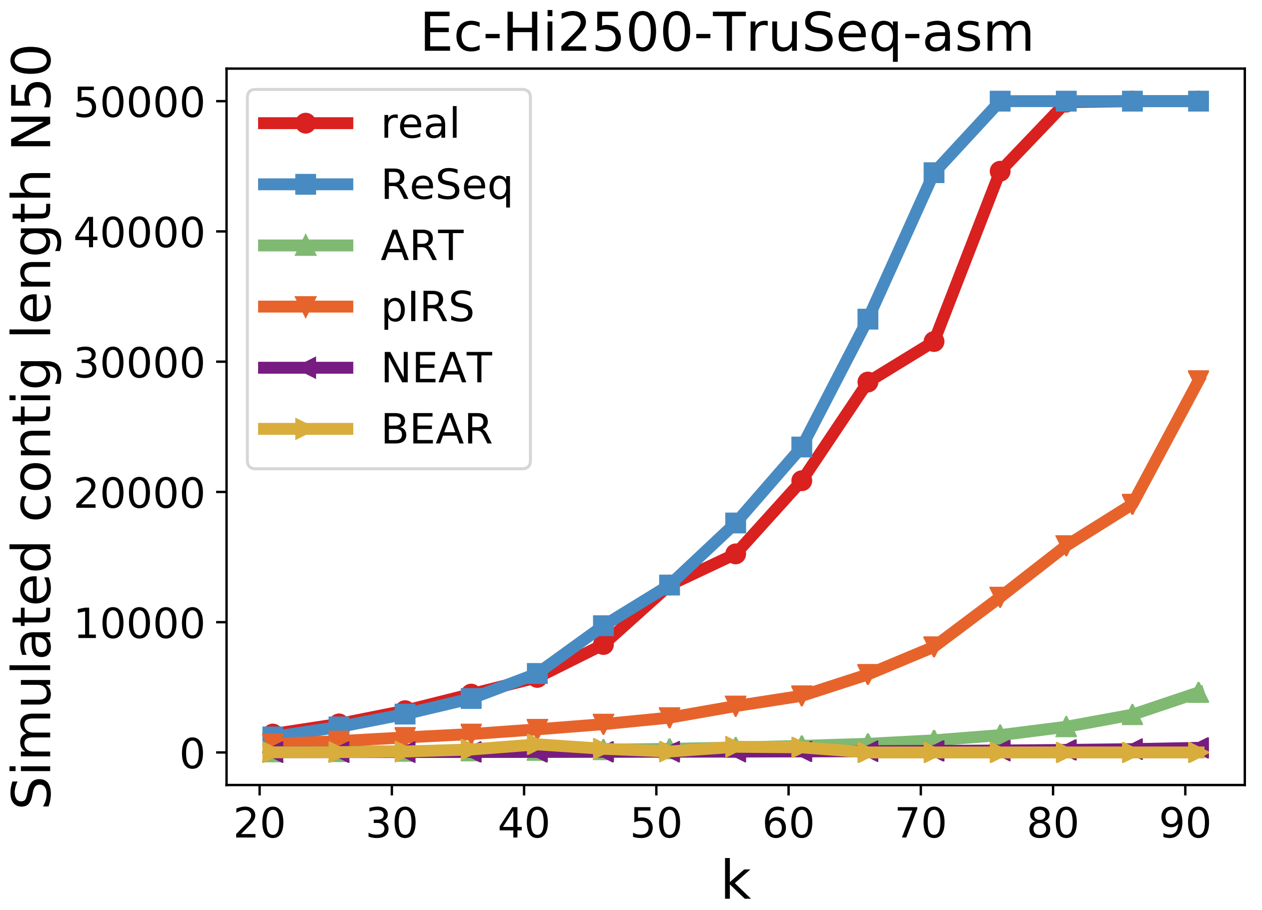

Fig. S23 Simulated contig N50 for real and simulated data for Ec-Hi2500-TruSeq-asm with the median coverage from Ec-Hi2500-TruSeq provided to all simulators except BEAR.

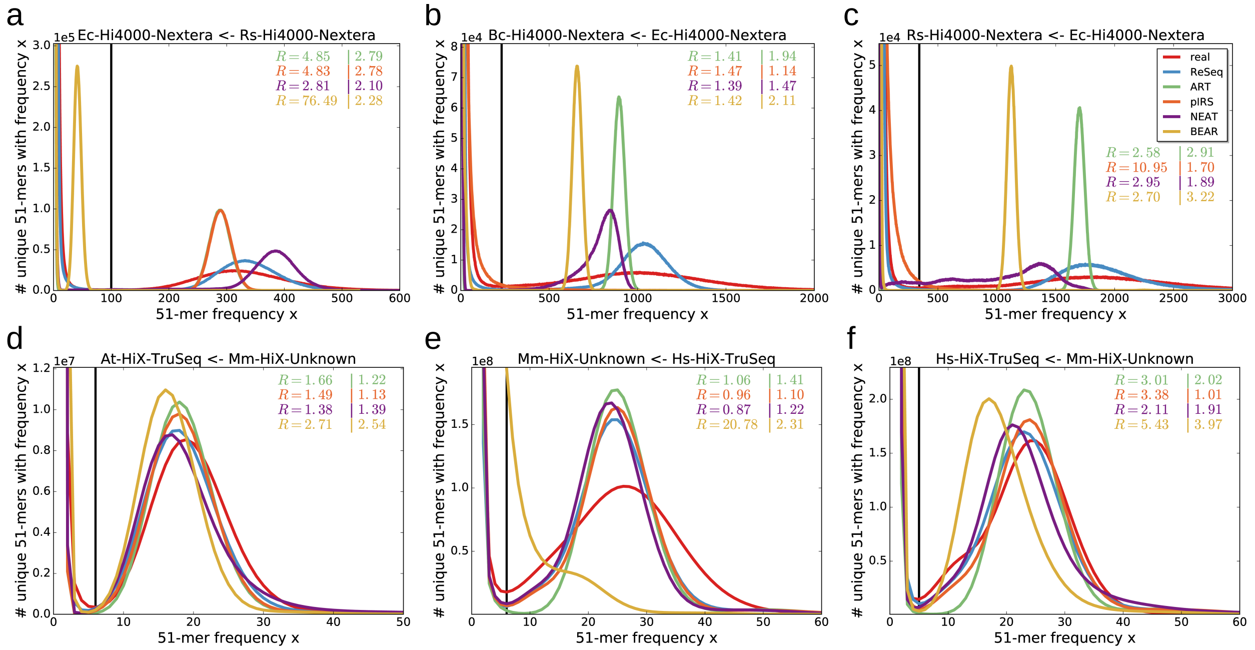

Fig. S24 51-mer spectra of real data and cross-species simulations. The simulation profiles are trained on a different training dataset. The pairings are given in the plot titles in the following notation: "simulated dataset $\leftarrow$ training dataset". The shape and position of peaks reflects the coverage distribution, while the exponential decrease at low frequencies is defined by systematic errors. The black lines show the minimum between the exponential decrease and signal peak for real data. The first value of R gives the sum of relative deviations for frequencies (x) up to the minimum. The second value of R gives the sum of absolute deviations for frequencies larger than the minimum. Both values are stated relative to ReSeq. When the signal peak of the simulation does not exist or starts before the minimum in real data the values lose their interpretability. This happens for pIRS (b-c), NEAT (a-c) and BEAR (a,c,e).

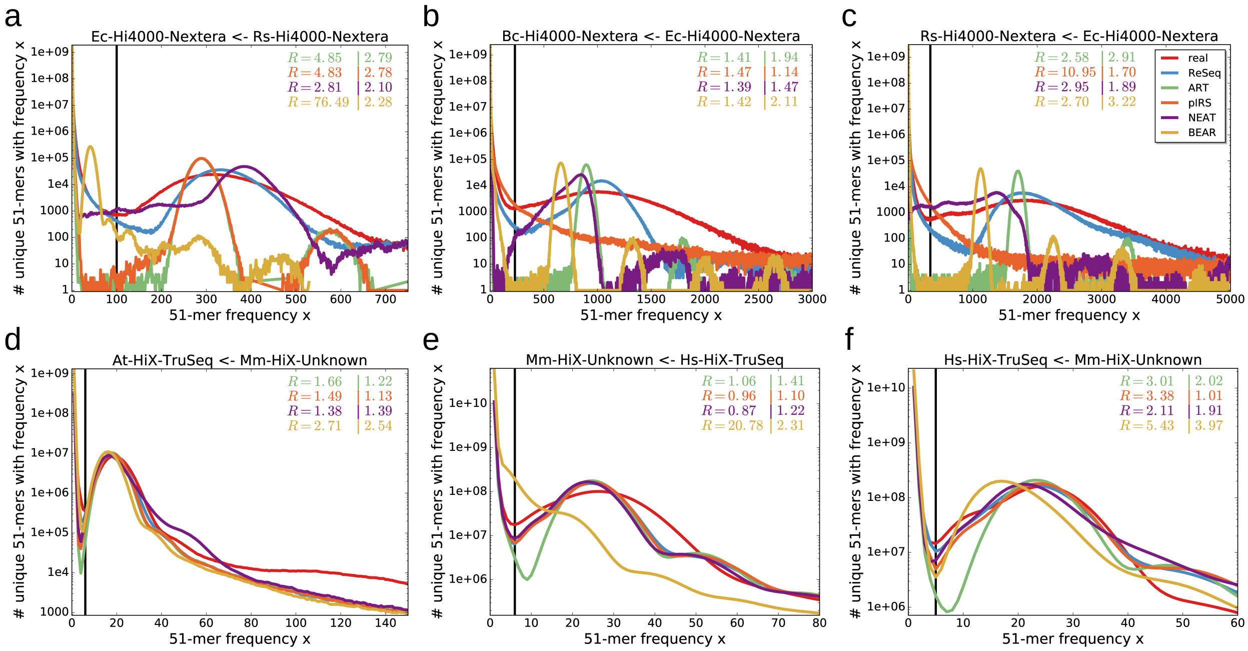

Fig. S25 51-mer spectra of real and cross-simulated data. The simulation profiles are trained on a different training dataset. The pairings are given in the plot titles in the following notation: "simulated dataset ← training dataset". The shape and position of peaks reflects the coverage distribution, while the exponential decrease at low frequencies is defined by systematic errors. The black lines show the minimum between the exponential decrease and signal peak for real data. The first value of R gives the sum of relative deviations for frequencies (x) up to the minimum. The second value of R gives the sum of absolute deviations for frequencies larger than the minimum. Both values are stated relative to ReSeq. When the signal peak of the simulation does not exist or starts before the minimum in real data, the values lose their interpretability, which happens for pIRS (b-c), NEAT (a-c) and BEAR (a,c,e).

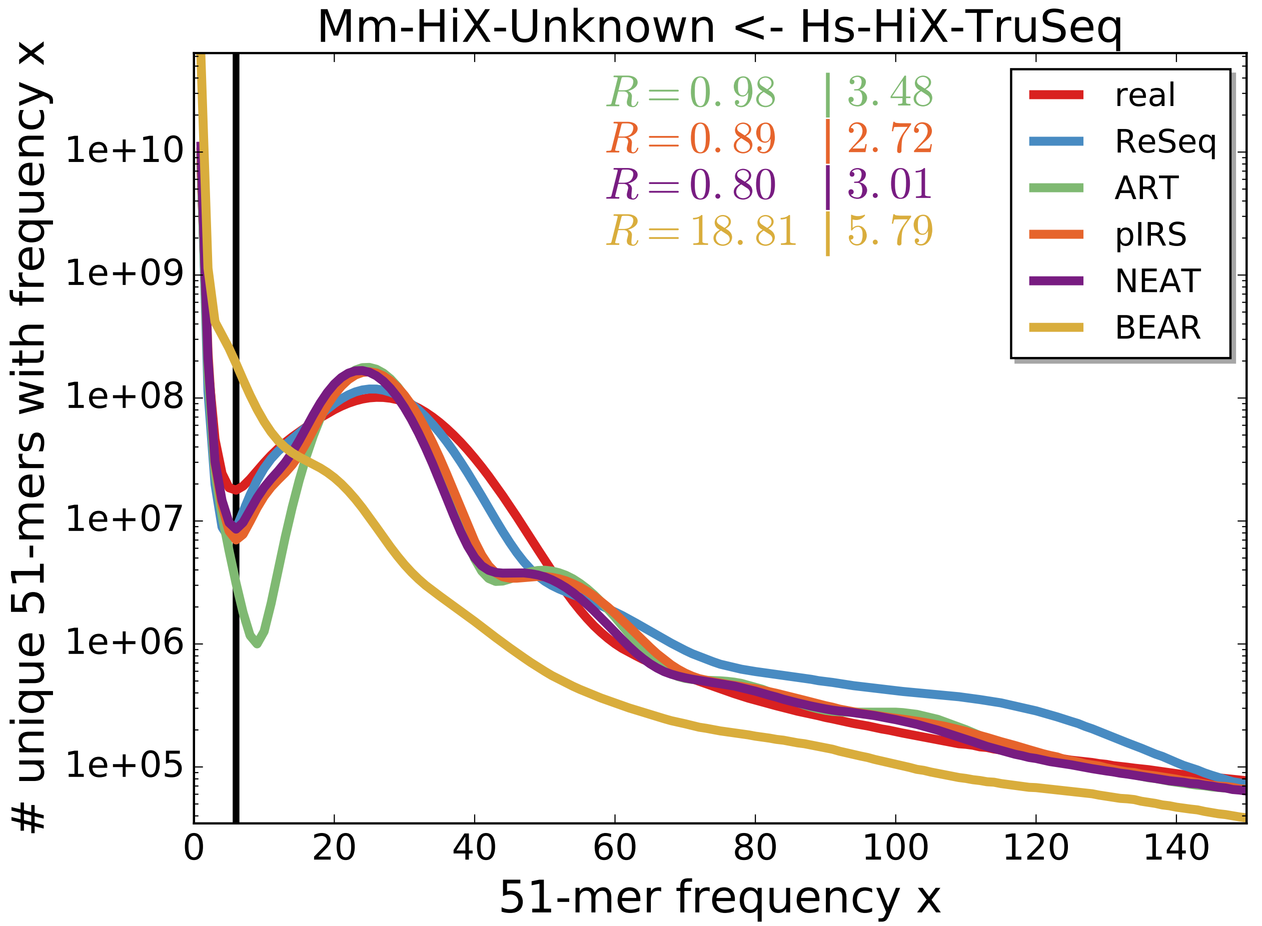

Fig. S26 51-mer spectrum for real and simulated data of Mm-HiX-Unknown. The simulations are trained on Hs-HiX-TruSeq. ReSeq and BEAR were provided with the reference bias trained on Mm-HiX-Unknown to reproduce the known fluctuations in chromosome abundance in this cell-line. The shape and position of peaks reflects the coverage distribution, while the exponential decrease at low frequencies is defined by systematic errors. The black lines show the minimum between the exponential decrease and signal peak for real data. The first value of R gives the sum of relative deviations for frequencies (x) up to the minimum. The second value of R gives the sum of absolute deviations for frequencies larger than the minimum. Both values are stated relative to ReSeq. When the signal peak of the simulation does not exist or starts before the minimum in real data the values lose their interpretability, which happens for BEAR.

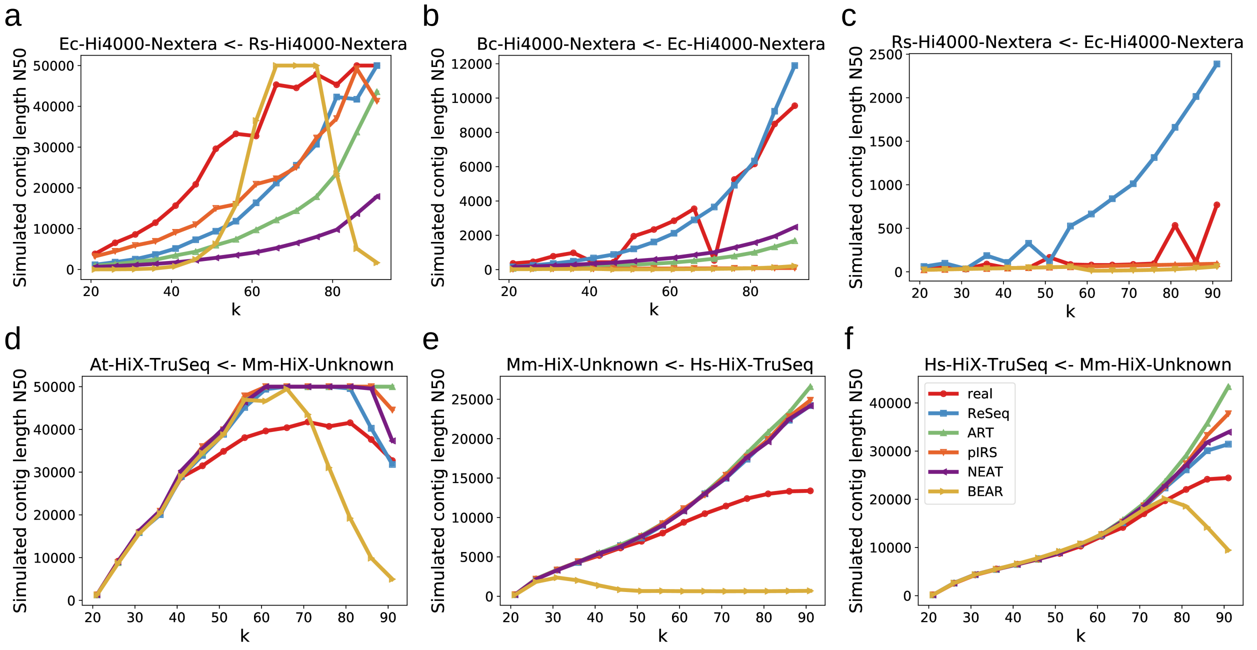

Fig. S27 Simulated contig N50 for real and simulated data. The simulation profiles are trained on a different training dataset. The pairings are given in the plot titles in the following notation: "simulated dataset ← training dataset". Note that: c) preqc crashed for ART and NEAT.

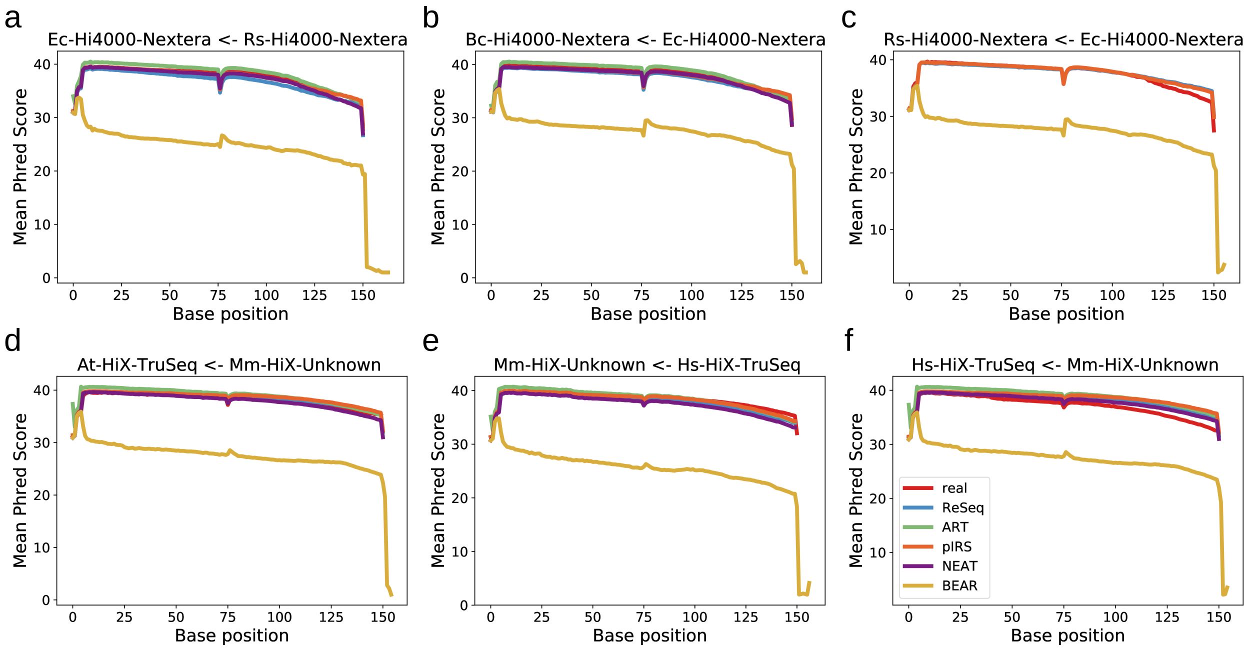

Fig. S28 Mean quality values by position for real and simulated data. The simulation profiles are trained on a different training dataset. The pairings are given in the plot titles in the following notation: "simulated dataset ← training dataset". Note that: c) preqc crashed for ART and NEAT.

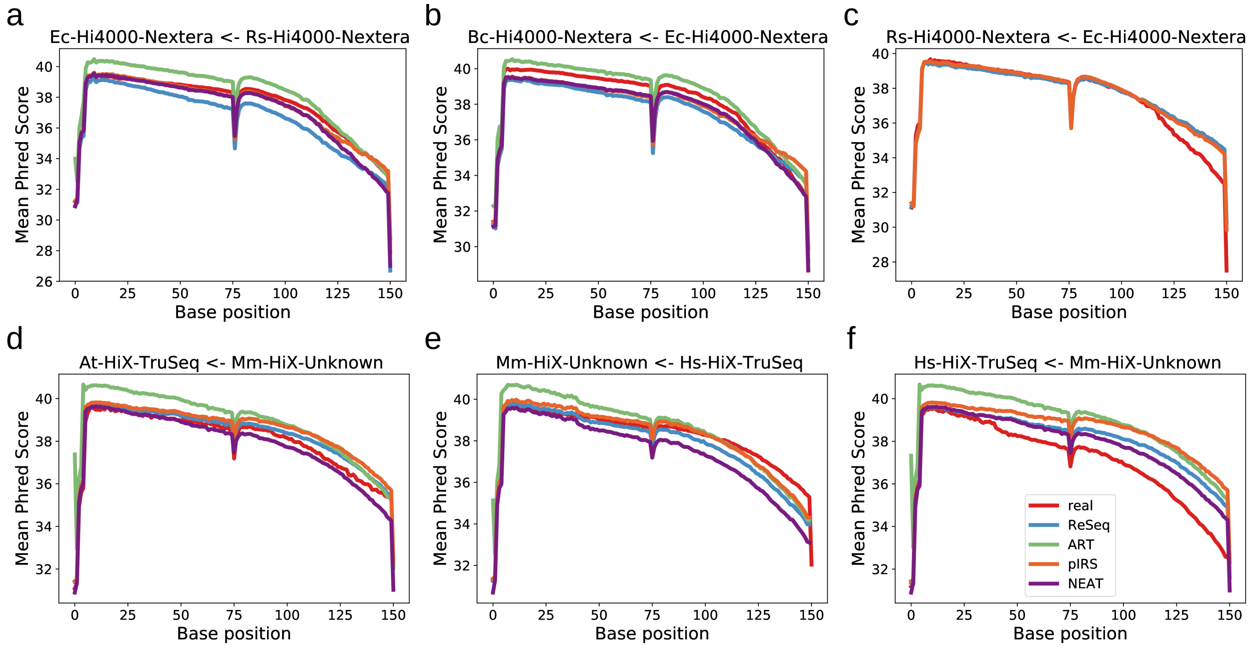

Fig. S29 Mean quality values by position for real and simulated data without BEAR. The simulation profiles are trained on a different training dataset. The pairings are given in the plot titles in the following notation: "simulated dataset ← training dataset". Note that: c) preqc crashed for ART and NEAT.

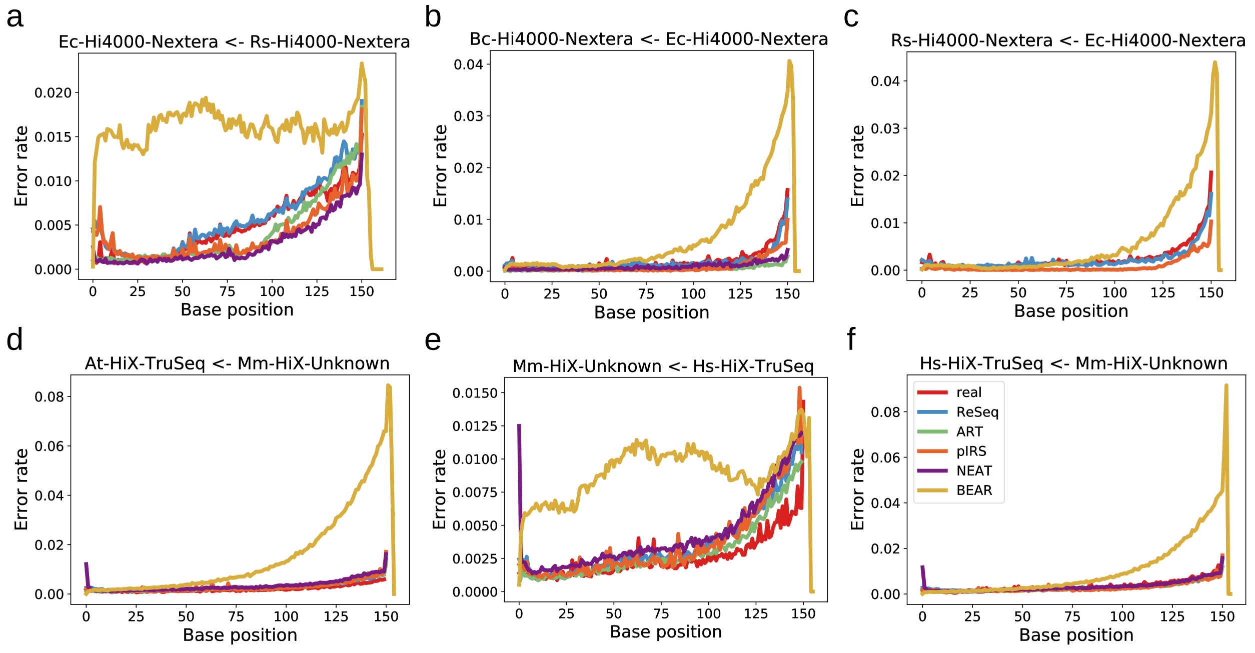

Fig. S30 Mean error rates by position for real and simulated data. The simulation profiles are trained on a different training dataset. The pairings are given in the plot titles in the following notation: "simulated dataset ← training dataset". Note that: c) preqc crashed for ART and NEAT.

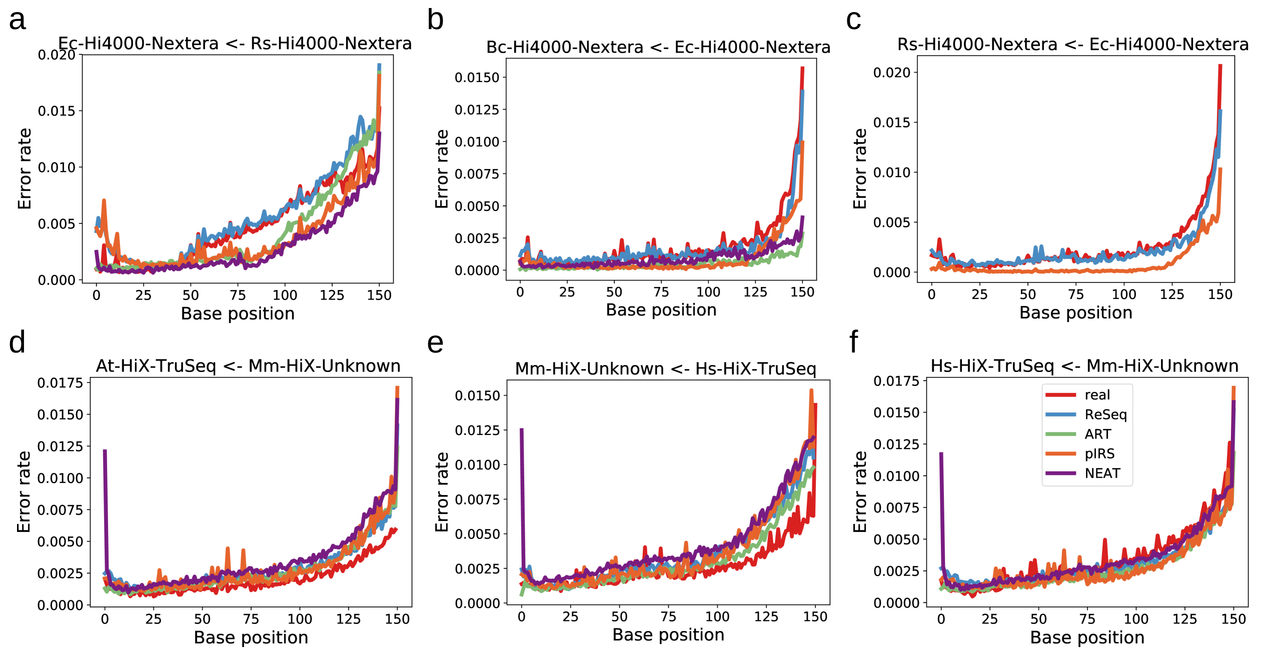

Fig. S31 Mean error rates by position for real and simulated data without BEAR. The simulation profiles are trained on a different training dataset. The pairings are given in the plot titles in the following notation: "simulated dataset ← training dataset". Note that: c) preqc crashed for ART and NEAT.

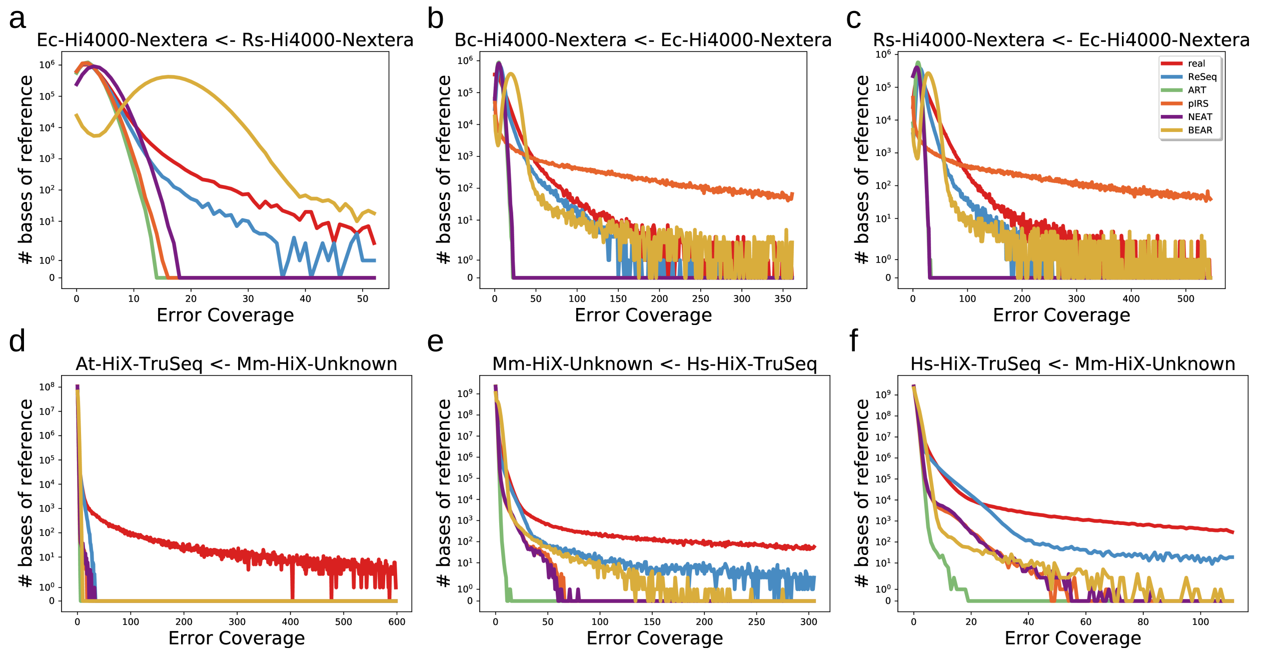

Fig. S32 Systematic errors of real and simulated data. The simulation profiles are trained on a different training dataset. The pairings are given in the plot titles in the following notation: "simulated dataset ← training dataset".

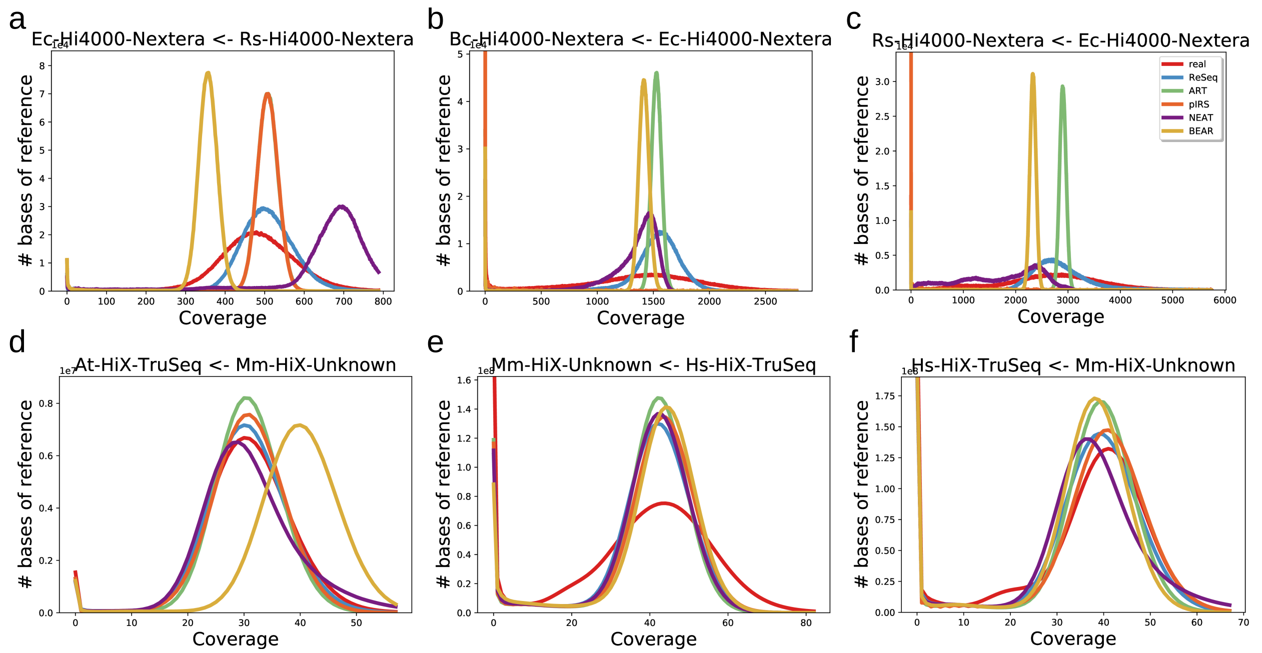

Fig. S33 Base-coverage distribution of real and simulated data. The simulation profiles are trained on a different training dataset. The pairings are given in the plot titles in the following notation: "simulated dataset ← training dataset".

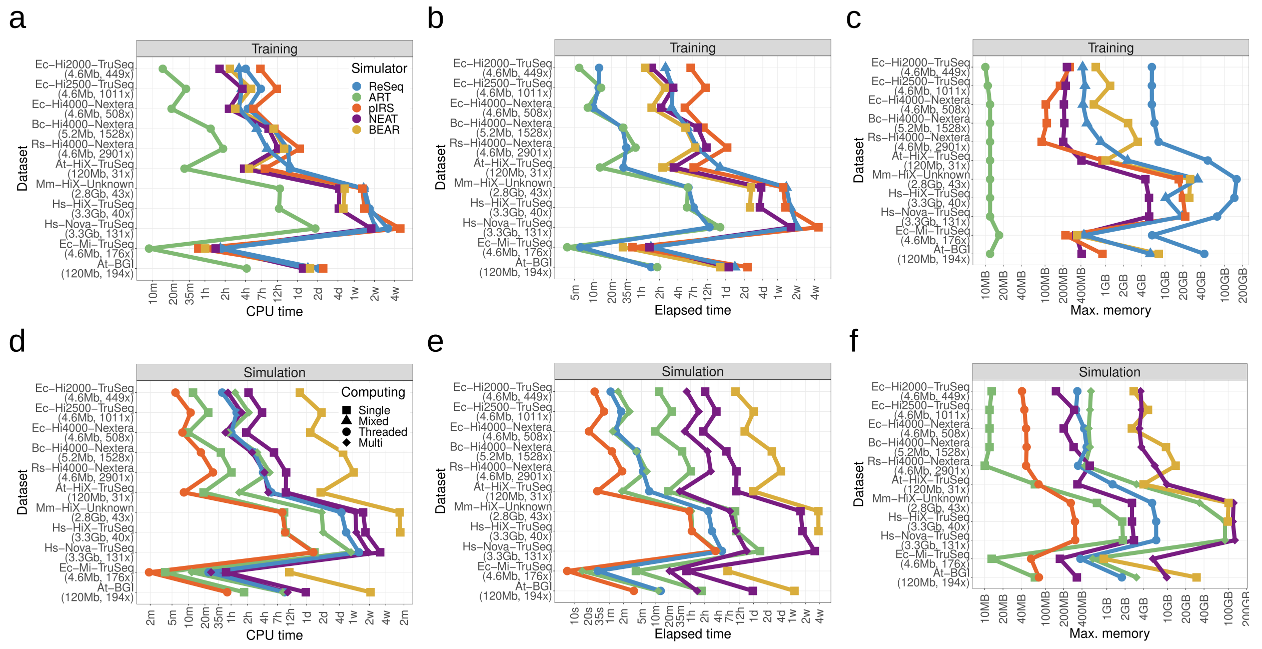

Fig. S34 Speed and memory benchmark of simulation process. The training part needs to be performed only once to generate the profiles. The simulation part needs to be done for every synthetic dataset created. All datasets have the reference size and the coverage specified. BEAR was omitted for Hs-Nova-TruSeq, because the estimated runtime was above three month. Single: Single thread per task, but often multiple task in parallel. Mixed: A mixture between single- and multi-threaded for memory optimization. Threaded: Multi-threaded execution. Multi: Using multiple instances for simulators without multi-threading support.

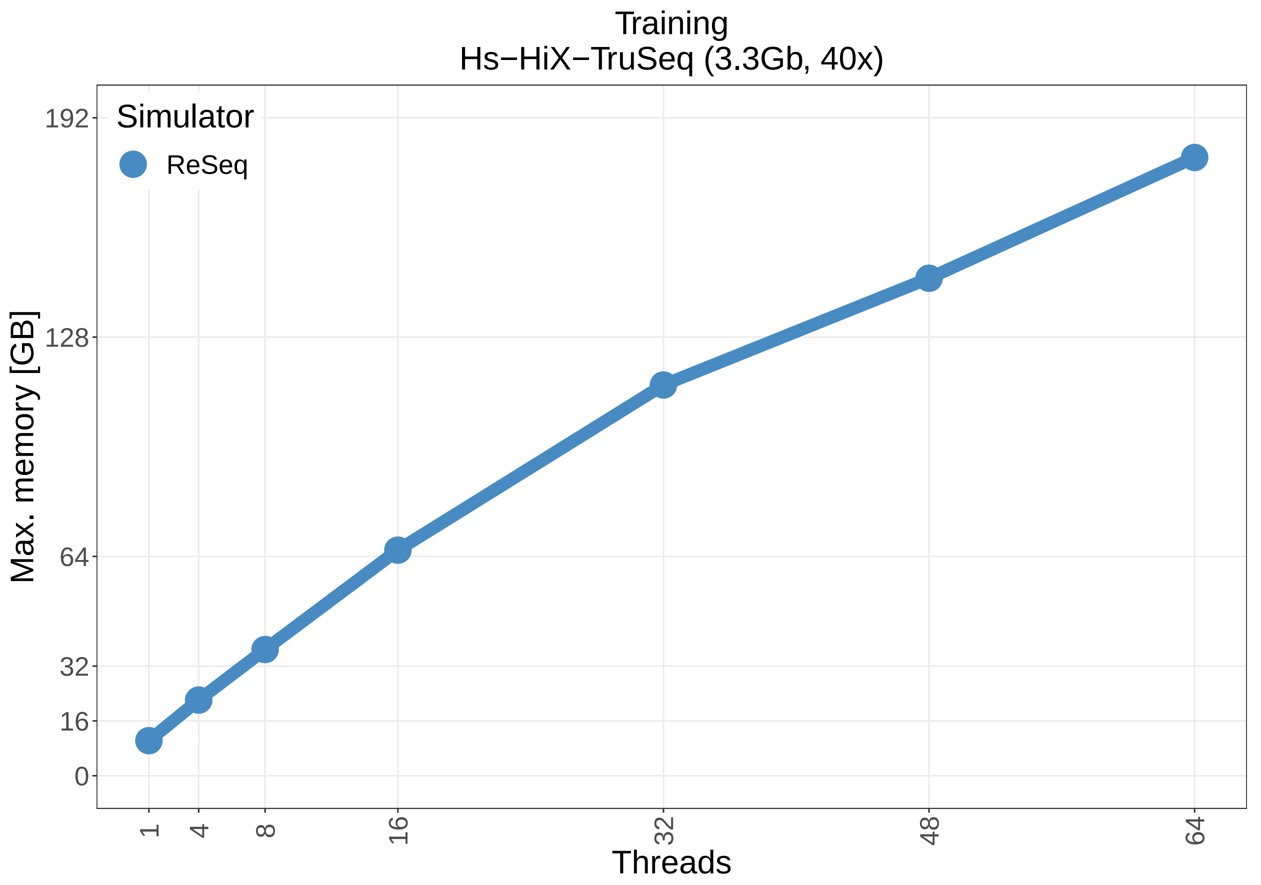

Fig. S35 Memory requirements for ReSeq training on Hs-HiX-TruSeq for different numbers of computing threads.

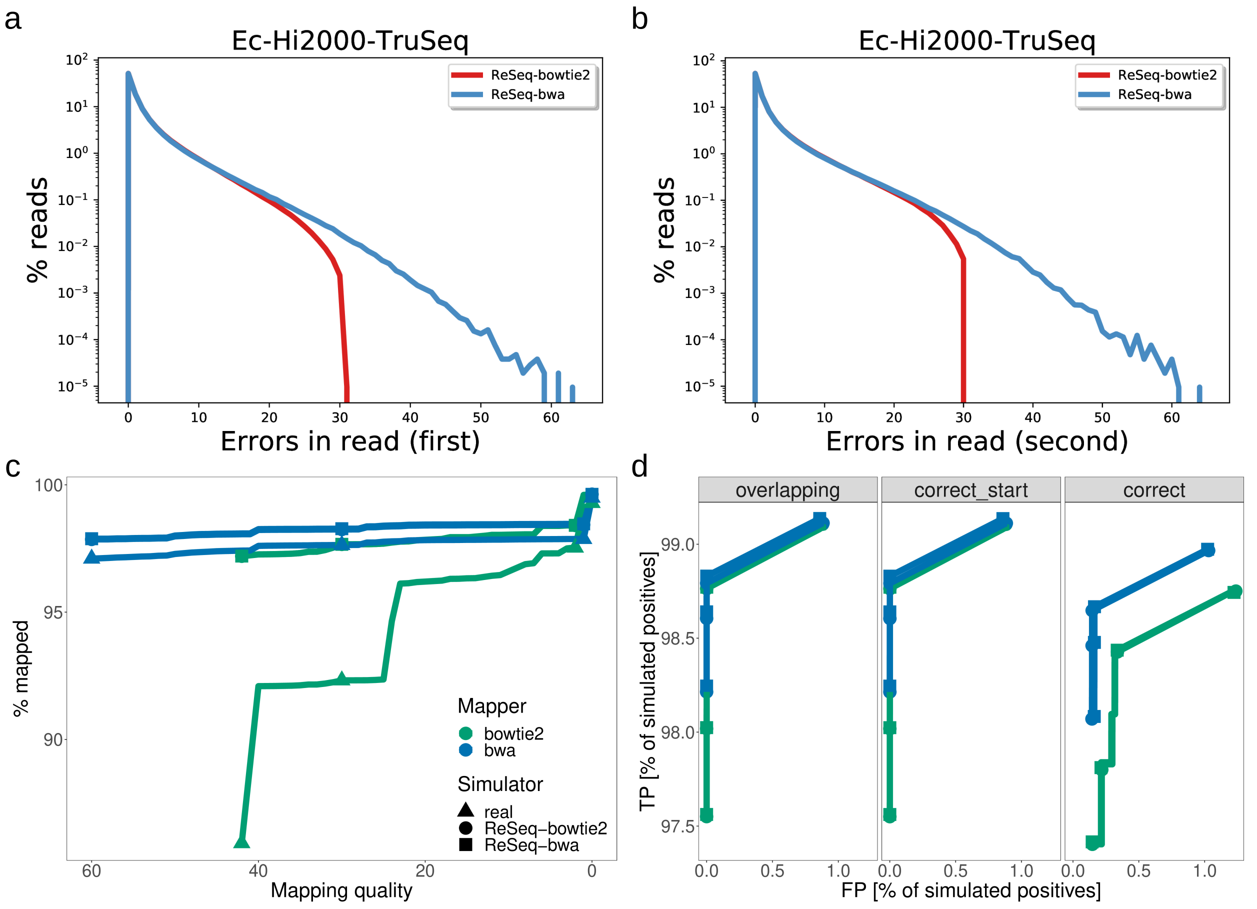

Fig. S36 Difference in errors per read between ReSeq trained on bowtie2 and bwa mappings. a,b) Number of errors observed by ReSeq in the Ec-Hi2000-TruSeq dataset using bowtie2 and bwa mappings of first (a) and second reads (b). c) Mapping rate over mapping quality for real and simulated data, with all reads removed that have more than 10 simulated errors. Real data are unfiltered. Mapping qualities are cumulative, i.e. all mapping qualities at the given score or higher. Markers are only shown for mapping qualities 0,2,30,42 for bowtie and 0,1,30,60 for bwa. Due to the highly-erroneous read removal, the mapping rates for bowtie2 are nearly identical independent of what mapper was used for the training data. d) Mapping accuracy for all mapping quality thresholds with all reads removed that have more than 10 simulated errors. Markers are only shown for mapping qualities 0,2,30,42 for bowtie and 0,1,30,60 for bwa. Positives are mapped reads, which fulfill the correctness criteria (TP) or do not (FP). Overlapping: True and mapped positions overlap independent of strand. Correct_start: Perfect match of start position and strand. Correct: Perfect match of start and end positions and strand. Due to the highly-erroneous read removal, bwa scores are almost identically and strongly improved, when requiring perfect matches, independent of what mapper was used for the training data.

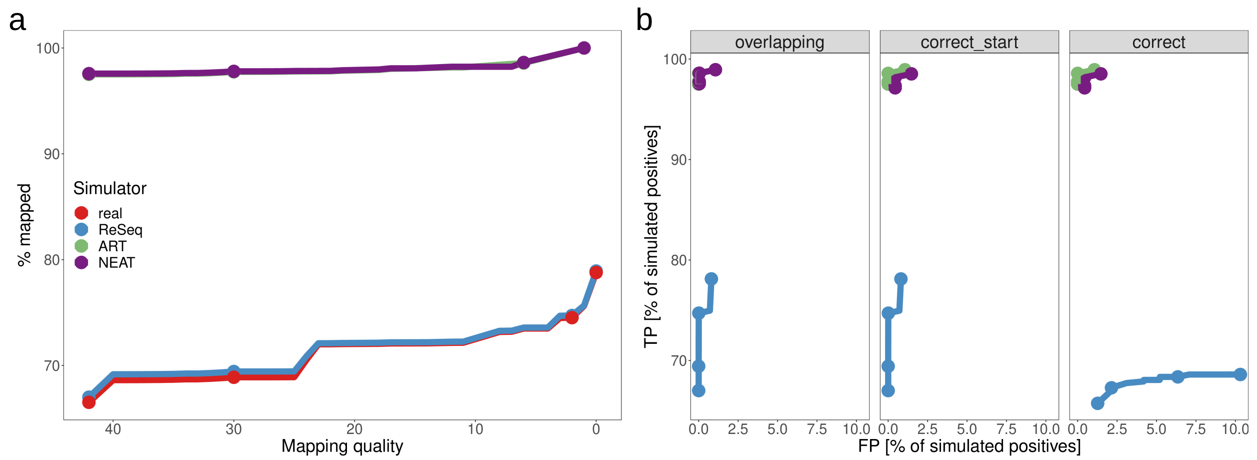

Fig. S37 Comparison of bowtie2 mapping on Ec-Hi4000-Nextera for different simulations. pIRS and BEAR have been omitted for this analysis due to their inaccurate representation of the real coverage distribution (S15g) or error rate (S5d) for this dataset. The discrepancies caused by not simulating adapters (ART, NEAT) are clearly visible. a) Mapping quality distribution. Mapping qualities are cumulative, i.e. all mapping qualities at the given score or higher. Markers are only shown for mapping qualities 0,2,30,42. Instead of 0 and 2, ART and NEAT show markers for mapping quality 1 and 6, which are the two lowest assigned qualities in their case. b) Mapping accuracy for all mapping quality thresholds. Markers are only shown for mapping qualities 0,2,30,42. Instead of 0 and 2, ART and NEAT show markers for mapping quality 1 and 6, which are the two lowest assigned qualities in their case. Positives are mapped reads, which fulfill the correctness criteria (TP) or do not (FP). Overlapping: True and mapped positions overlap independent of strand. Correct_start: Perfect match of start position and strand. Correct: Perfect match of start and end positions and strand.

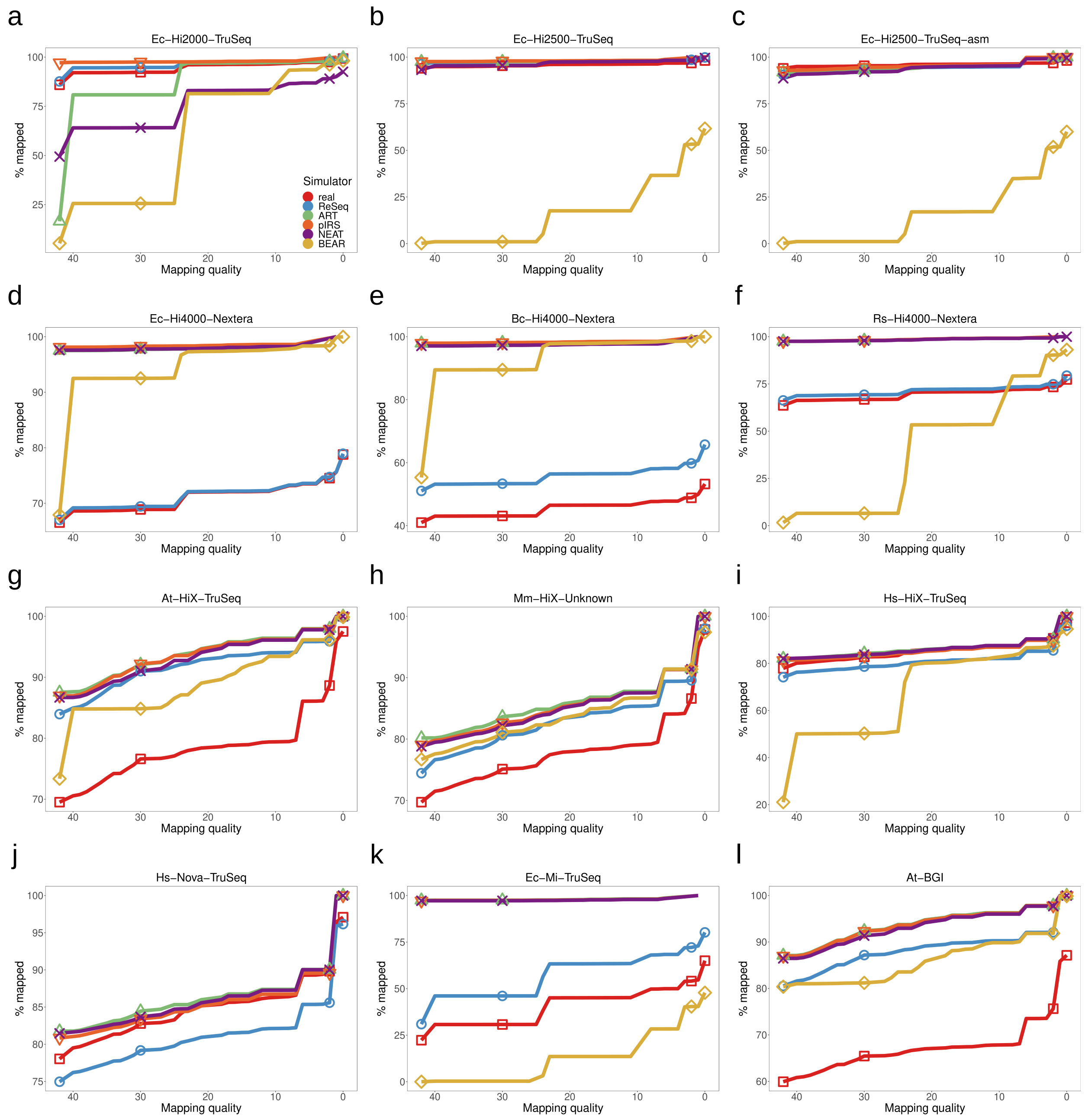

Fig. S38 Bowtie2 mapping qualities for real and simulated datasets. Mapping qualities are cumulative, i.e. all mapping qualities at the given score or higher. Markers are only shown for mapping qualities 0,2,30,42. j) BEAR was omitted due to excessive runtimes.

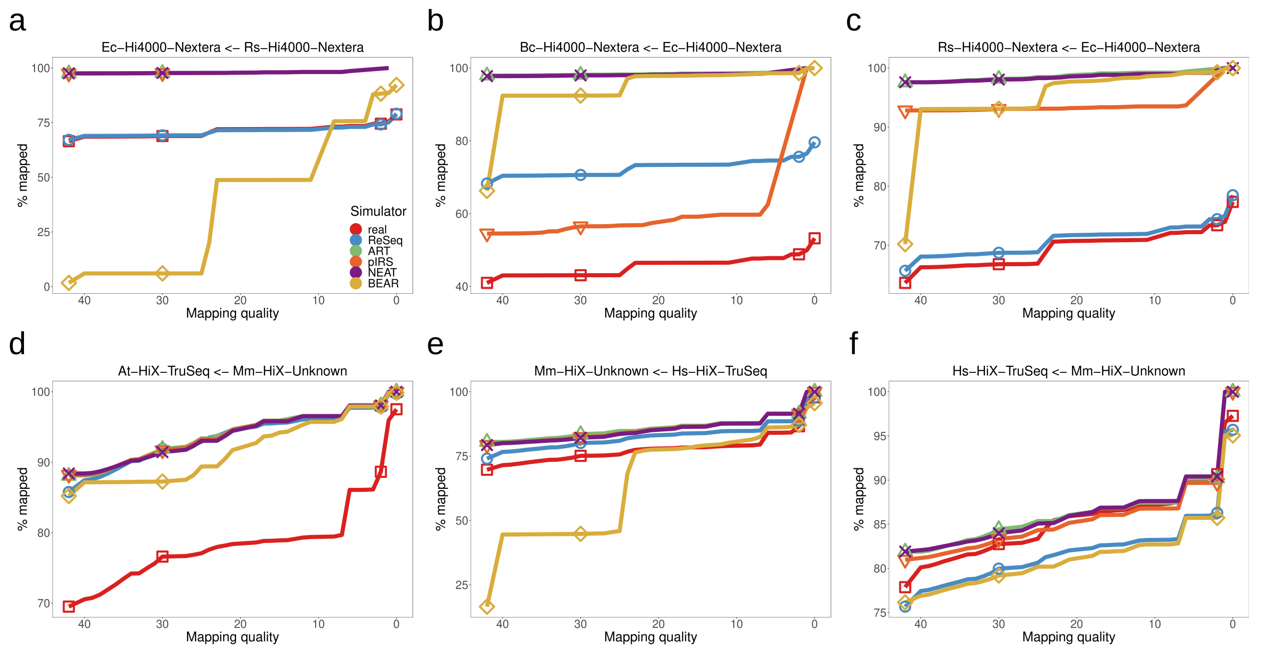

Fig. S39 Bowtie2 mapping qualities for real and simulated datasets. The simulation profiles are trained on a different training dataset. The pairings are given in the plot titles in the following notation: "simulated dataset ← training dataset". Mapping qualities are cumulative, i.e. all mapping qualities at the given score or higher. Markers are only shown for mapping qualities 0,2,30,42

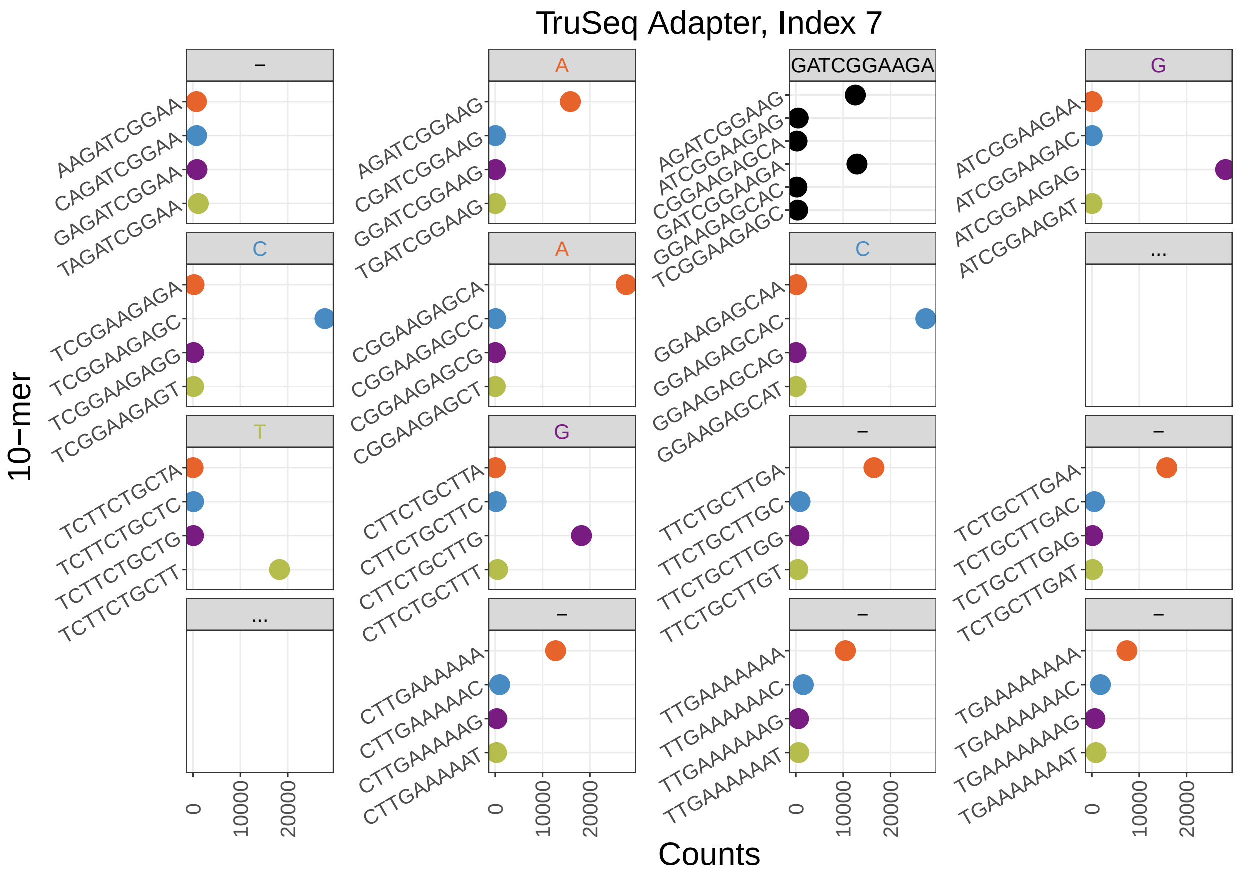

Fig. S40 10-mers and their counts for the automatic adapter detection in first reads of Ec-Hi2000-TruSeq. The panel with black dots and a full 10-mer as label is the starting 10-mer. Only the six 10-mers with the highest counts are shown for this panel. The other panels show the four possible extending 10-mers for both sides with the true adapter sequence in the label. The direction is from left to right and then from top to bottom. Empty panels with dots in the label represent positions skipped in the plotting. The first and the last panel are not included in the detected adapter sequence, because they fulfill the stopping requirement of the second highest count being bigger than one fifth of the highest count. The poly-A tail, visible in the six panels after the end of the true adapter sequence, is cut from the detected adapter sequence. The only panel that has more than one dominant 10-mer is the starting panel, but the two dominant 10-mers in it are just shifts by one position. Therefore, independent of the selected 10-mer the final detected adapter sequence is the same.

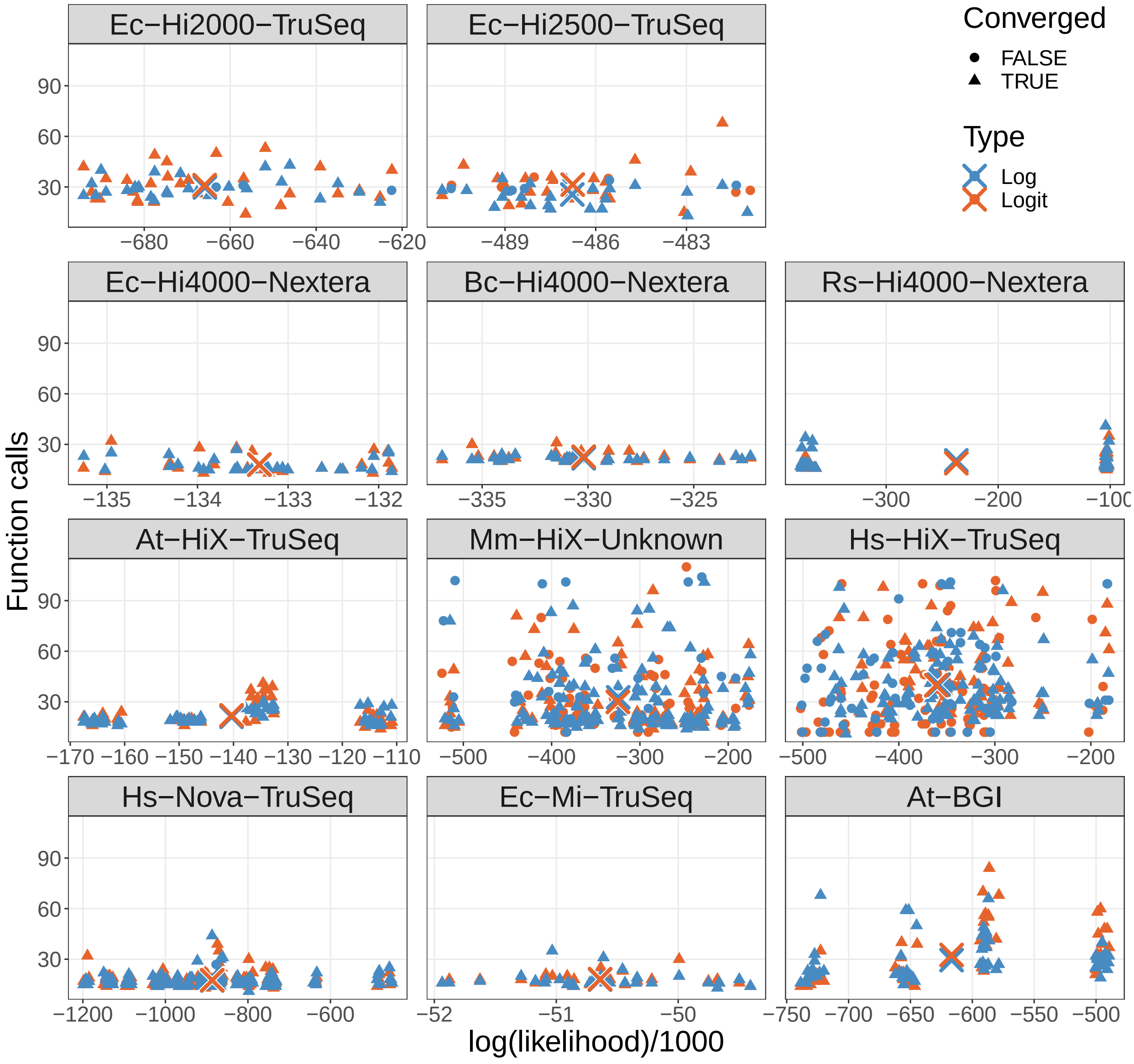

Fig. S41 Comparison of log and logit link function for the GC bias. Dots and triangles are individual fits. Crosses are the mean value for that dataset.

Supplementary Tables

Table S1 Used simulator versions.

Simulator	Version
ReSeq	1.1
	3279e764f74809460e4c702fa9434dabf277e83a
pIRS	2.0.0
	bee9b594f4d0e10580aae77ec411cecec4a77219
ART	2.5.8
	MountRainier-2016-06-05
NEAT	v2.0
	cdb869a2451221ab57bffeffe50329cdd1467c2f
BEAR	02274019ce7c2ac70c2f642368bc0682fb97446a

Table S2 Used program versions.

Program	Version
bcl2fastq	v2.20.0.422
bcftools [2]	1.9
bedtools [3]	v2.25.0
bowtie2 [4]	2.2.5
bwa mem [5]	0.7.13-r1126
freebayes [6]	v1.3.1-dirty
GNU time	1.7
igv [7, 8]	2.6.3
jellyfish [9]	2.0.0
kmc [10]	3.1.1 (2019-05-19)
pilon [11]	1.21
preqc [12]	0.10.14
quast [13]	4.4
samtools [14]	1.9
sga [15]	0.10.14
snakemake [16]	3.5.5
soap [17]	2.21

Supplementary Formulas

Here the exact likelihood and gradient formulas that are used in the program for the coverage model fit are derived, starting from the formulas and information in the main document. While the formulas from the main document are repeated, only new symbols will be explained.

Step 1 Poisson

Internally, the normalization N is always part of the GC bias, because it is absorbed by it for the log-likelihood calculation during this step.

$$\begin{aligned}\mu_n &= N b_{GC}(GC_n) b_{start,n} b_{end,n} \\ &= b_{GC}^N b_{start,n} b_{end,n} \\ &= b_{GC}^N \hat{\mu}_n\end{aligned}$$

$$L_P = \prod_n \frac{\mu_n^{k_n}}{k_n!} e^{-\mu_n}$$

$$\begin{aligned}\log(L_P) &= \sum_n \left[k_n \log(\mu_n) - \log(k_n!) - \mu_n\right] \\ &= \left[-\sum_n \log(k_n!)\right] + \sum_{GC} \sum_{n(GC)} \left[k_n \log(b_{GC}^N \hat{\mu}_n) - b_{GC}^N \hat{\mu}_n\right] \\ &= \left[-\sum_n \log(k_n!)\right] + \sum_{GC} \sum_{n(GC)} \left[k_n \log(b_{GC}^N) + k_n \log(\hat{\mu}_n) - b_{GC}^N \hat{\mu}_n\right] \\ &= \left[-\sum_n \log(k_n!)\right] + \sum_{GC} \left\{\log(b_{GC}^N) \left[\sum_{n(GC)} k_n\right] + \left[\sum_{n(GC)} k_n \log(\hat{\mu}_n)\right] - b_{GC}^N \left[\sum_{n(GC)} \hat{\mu}_n\right]\right\}\end{aligned}$$

$$\frac{\partial \log(L_P)}{\partial b_{GC}^N} = \frac{1}{b_{GC}^N} \left[\sum_{n(GC)} k_n\right] - \left[\sum_{n(GC)} \hat{\mu}_n\right]$$

$$\begin{aligned}0 = \frac{\partial \log(L_P)}{\partial b_{GC}^N} \quad &\Leftrightarrow \quad \sum_{n(GC)} \hat{\mu}_n = \frac{1}{b_{GC}^N} \sum_{n(GC)} k_n \\ &\Leftrightarrow \quad b_{GC}^N = \frac{\sum_{n(GC)} k_n}{\sum_{n(GC)} \hat{\mu}_n} = \frac{①}{②}\end{aligned}$$

In the software, the variable names are ① gc_count_[gc] and ② gc_bias_sum[gc].second. When we use the calculated b_{GC}^N in the likelihood, we always have perfect normalization, thus the normalization is absorbed by the GC bias.

$$\begin{aligned}\log(L_P) &= \left[-\sum_n \log(k_n!)\right] + \sum_{GC} \left\{\left[\sum_{n(GC)} k_n \log(\hat{\mu}_n)\right] + \left[\sum_{n(GC)} k_n\right] \log(b_{GC}^N) - \left[\sum_{n(GC)} k_n\right]\right\} \\ &= ③ + \sum_{GC} \left\{④ + ① \log(b_{GC}^N) - ①\right\}\end{aligned}$$

③ loglike_poisson_base_, ④ gc_bias_sum[gc].first. This leaves the $b_{f,p}$ to be fitted, thus we need their gradients.

$$\frac{\partial b^N_{GC}}{\partial b_{f,p}} = -\frac{b^N_{GC}}{\sum_{n(GC)} \hat{\mu}_n} \sum_{n(GC)} \frac{\partial \hat{\mu}_n}{\partial b_{f,p}}$$

$$\begin{aligned}
\frac{\partial \log(L_P)}{\partial b_{f,p}} &= \sum_{GC} \left\{ \left[\sum_{n(GC)} \frac{k_n}{\hat{\mu}_n} \frac{\partial \hat{\mu}_n}{\partial b_{f,p}} \right] + \frac{\sum_{n(GC)} k_n}{b^N_{GC}} \frac{\partial b^N_{GC}}{\partial b_{f,p}} \right\} \\
&= \sum_{GC} \left\{ \left[\sum_{n(GC)} \frac{k_n}{\hat{\mu}_n} \frac{\partial \hat{\mu}_n}{\partial b_{f,p}} \right] - \left[\sum_{n(GC)} \hat{\mu}_n \right] \frac{b^N_{GC}}{\sum_{n(GC)} \hat{\mu}_n} \sum_{n(GC)} \frac{\partial \hat{\mu}_n}{\partial b_{f,p}} \right\} \\
&= \left[\sum_n \frac{k_n}{\hat{\mu}_n} \frac{\partial \hat{\mu}_n}{\partial b_{f,p}} \right] - \left\{ \sum_{GC} b^N_{GC} \sum_{n(GC)} \left[\frac{\partial \hat{\mu}_n}{\partial b_{f,p}} \right] \right\} \\
&= ⑤ - \left\{ \sum_{GC} b^N_{GC} \sum_{n(GC)} ⑥ \right\}
\end{aligned}$$

⑤ grad_sur_[sur], ⑥ grad_gc_bias_sum[gc][sur]

For the sum version of the flanking bias, this leads to the following:

$$b_{start,n} = \frac{2}{1 + e^{-\sum_p b_{f(p,start),p}}}$$

$$\begin{aligned}
\frac{\partial b_{start,n}}{\partial b_{f,p}} &= -\frac{b_{start,n}}{1 + e^{-\sum_{\tilde{p}} b_{f(\tilde{p},start),\tilde{p}}}} e^{-\sum_{\tilde{p}} b_{f(\tilde{p},start),\tilde{p}}} \left(-\delta_{f(\tilde{p},start),f} \right) \\
&= b_{start,n} \delta_{f(\tilde{p},start),f} \frac{e^{-\sum_{\tilde{p}} b_{f(\tilde{p},start),\tilde{p}}} + 1 - 1}{1 + e^{-\sum_{\tilde{p}} b_{f(\tilde{p},start),\tilde{p}}}} \\
&= b_{start,n} \delta_{f(\tilde{p},start),f} \left(1 - \frac{1}{1 + e^{-\sum_{\tilde{p}} b_{f(\tilde{p},start),\tilde{p}}}} \right) \\
&= b_{start,n} \delta_{f(\tilde{p},start),f} \left(1 - \frac{b_{start,n}}{2} \right)
\end{aligned}$$

$$\begin{aligned}
\frac{\partial \hat{\mu}_n}{\partial b_{f,p}} &= \frac{\partial b_{start,n}}{\partial b_{f,p}} b_{end,n} + b_{start,n} \frac{\partial b_{end,n}}{\partial b_{f,p}} \\
&= b_{start,n} \delta_{f(\tilde{p},start),f} \left(1 - \frac{b_{start,n}}{2} \right) b_{end,n} + b_{start,n} b_{end,n} \delta_{f(\tilde{p},end),f} \left(1 - \frac{b_{end,n}}{2} \right) \\
&= \hat{\mu}_n \left[\delta_{f(\tilde{p},start),f} \left(1 - \frac{b_{start,n}}{2} \right) + \delta_{f(\tilde{p},end),f} \left(1 - \frac{b_{end,n}}{2} \right) \right]
\end{aligned}$$

$$\begin{aligned}
\frac{\partial \log(L_{PS})}{\partial b_{f,p}} = & \left[\sum_n k_n \left[\delta_{f(\tilde{p},start),f} \left(1 - \frac{b_{start,n}}{2} \right) + \delta_{f(\tilde{p},end),f} \left(1 - \frac{b_{end,n}}{2} \right) \right] \right] \\
& - \left\{ \sum_{GC} b^N_{GC} \left[\sum_{n(GC)} \hat{\mu}_n \left[\delta_{f(\tilde{p},start),f} \left(1 - \frac{b_{start,n}}{2} \right) + \delta_{f(\tilde{p},end),f} \left(1 - \frac{b_{end,n}}{2} \right) \right] \right] \right\}
\end{aligned}$$

Inspired by biases of the four nucleotides at a position not being independent, we do not directly use $b_{f,p}$ as fit parameters, but $\tilde{b}_{f,p}$. However, we still have 4 parameters per position, because our attempts to reduce this to 3 did not improve the fitting.

$$b_{f,p} = \tilde{b}_{f,p} - \frac{\sum_{\tilde{s}} \tilde{b}_{\tilde{s},p}}{4} + \delta_{p0}\tilde{b}_{shift}$$

$\tilde{b}_{shift}$ is an additional parameter that can shift the spread of different $\sum_p b_{f(p,start),p}$ to an ideal range for the inverse logit transformation.

$$\begin{aligned} \frac{\partial \log(L_{PS})}{\partial \tilde{b}_{f,p}} &= \sum_{\tilde{s}} \frac{\partial b_{\tilde{s},p}}{\partial \tilde{b}_{f,p}} \frac{\partial \log(L_P)}{\partial b_{\tilde{s},p}} \\ &= \frac{\partial \log(L_P)}{\partial b_{f,p}} - \frac{\sum_{\tilde{s}} \frac{\partial \log(L_P)}{\partial b_{\tilde{s},p}}}{4} \end{aligned}$$

$$\begin{aligned} \frac{\partial \log(L_{PS})}{\partial \tilde{b}_{shift}} &= \sum_{\tilde{s}} \frac{\partial b_{\tilde{s},0}}{\partial \tilde{b}_{shift}} \frac{\partial \log(L_P)}{\partial b_{\tilde{s},0}} \\ &= \sum_{\tilde{s}} \frac{\partial \log(L_P)}{\partial b_{\tilde{s},0}} \end{aligned}$$

For the product version of the flanking bias, the likelihood and gradients are the following.

$$b_{start,n} = \prod_p b_{f(p,start),p}$$

$$\frac{\partial b_{start,n}}{\partial b_{f,p}} = \delta_{f(p,start),f} \frac{b_{start,n}}{b_{f,p}}$$

$$\begin{aligned} \frac{\partial \hat{\mu}_n}{\partial b_{f,p}} &= \frac{\partial b_{start,n}}{\partial b_{f,p}} b_{end,n} + b_{start,n} \frac{\partial b_{end,n}}{\partial b_{f,p}} \\ &= \delta_{f(p,start),f} \frac{b_{start,n}}{b_{f,p}} b_{end,n} + b_{start,n} \delta_{f(p,end),f} \frac{b_{end,n}}{b_{f,p}} \\ &= \frac{\hat{\mu}_n}{b_{f,p}} \left(\delta_{f(p,start),f} + \delta_{f(p,end),f}\right) \end{aligned}$$

$$\begin{aligned} \frac{\partial \log(L_{PP})}{\partial b_{f,p}} = & \left[\sum_n \frac{k_n}{b_{f,p}} \left(\delta_{f(\tilde{p},start),f} + \delta_{f(\tilde{p},end),f}\right)\right] \\ & - \left\{\sum_{GC} b_{GC}^N \left[\sum_{n(GC)} \frac{\hat{\mu}_n}{b_{f,p}} \left(\delta_{f(\tilde{p},start),f} + \delta_{f(\tilde{p},end),f}\right)\right]\right\} \end{aligned}$$

Similar to the sum version, the real fit parameters are $\tilde{b}_{f,p}$.

$$b_{f,p} = \frac{4\tilde{b}_{f,p}}{\sum_{\tilde{s}} \tilde{b}_{\tilde{s},p}}$$

$$\begin{aligned}\frac{\partial b_{s,p}}{\partial \tilde{b}_{f,p}} &= 4\frac{\delta_{s,f}\left[\sum_{\tilde{s}}\tilde{b}_{\tilde{s},p}\right]-\tilde{b}_{s,p}}{\left[\sum_{\tilde{s}}\tilde{b}_{\tilde{s},p}\right]^2} \\ &= \frac{4\delta_{s,f}-\frac{4\tilde{b}_{s,p}}{\sum_{\tilde{s}}\tilde{b}_{\tilde{s},p}}}{\sum_{\tilde{s}}\tilde{b}_{\tilde{s},p}} \\ &= \frac{4\delta_{s,f}-b_{s,p}}{\sum_{\tilde{s}}\tilde{b}_{\tilde{s},p}}\end{aligned}$$

$$\begin{aligned}\frac{\partial\log(L_{PP})}{\partial\tilde{b}_{f,p}} &= \sum_s\frac{\partial b_{s,p}}{\partial\tilde{b}_{f,p}}\frac{\partial\log(L_P)}{\partial b_{s,p}} \\ &= \frac{4\frac{\partial\log(L_P)}{\partial b_{f,p}}-\left[\sum_s b_{s,p}\frac{\partial\log(L_P)}{\partial b_{s,p}}\right]}{\sum_{\tilde{s}}\tilde{b}_{\tilde{s},p}}\end{aligned}$$

Step 2 GC bias spline

$$b_{GC,spline}(GC) = c_1(GC) + c_2(GC)x(GC) + c_3(GC)x^2(GC) + c_4(GC)x^3(GC)$$

$$c_l(GC) = \sum_{j=1}^{6} t_{l,j}(GC)s_j$$

s_j are the six spline parameters, $t_{l,j}(GC)$ are coefficients calculated from the knot positions and $x(GC)$ is the distance to the last knot.

$$\frac{\partial b_{GC,spline}(GC)}{\partial s_{\tilde{j}}} = t_{1,\tilde{j}}(GC) + t_{2,\tilde{j}}(GC)x(GC) + t_{3,\tilde{j}}(GC)x^2(GC) + t_{4,\tilde{j}}(GC)x^3(GC)$$

The likelihood is the Poisson likelihood without the substitution of b^N_{GC}

$$\begin{aligned}\log(L_B) &= \left[-\sum_n\log(k_n!)\right] + \sum_{GC}\left\{\left[\sum_{n(GC)}k_n\log(\hat{\mu}_n)\right] + \left[\sum_{n(GC)}k_n\right]\log(b^N_{GC}) - b^N_{GC}\left[\sum_{n(GC)}\hat{\mu}_n\right]\right\} \\ &= \textcircled{3} + \sum_{GC}\left\{\textcircled{4} + \textcircled{1}\log(b^N_{GC}) - b^N_{GC}\textcircled{2}\right\}\end{aligned}$$

$$\begin{aligned}\frac{\partial\log(L_B)}{\partial s_{\tilde{j}}} &= \frac{\partial\log(L_B)}{\partial b^N_{GC}}\frac{\partial b^N_{GC}}{\partial b_{GC,spline}}\frac{\partial b_{GC,spline}}{\partial s_{\tilde{j}}} \\ &= \sum_{GC}\left\{\left(\frac{1}{b^N_{GC}}\left[\sum_{n(GC)}k_n\right] - \left[\sum_{n(GC)}\hat{\mu}_n\right]\right)\frac{\partial b^N_{GC}}{\partial b_{GC,spline}}\frac{\partial b_{GC,spline}}{\partial s_{\tilde{j}}}\right\} \\ &= \sum_{GC}\left\{\left(\left[\sum_{n(GC)}k_n\right] - b^N_{GC}\left[\sum_{n(GC)}\hat{\mu}_n\right]\right)\left(\frac{1}{b^N_{GC}}\frac{\partial b^N_{GC}}{\partial b_{GC,spline}}\right)\frac{\partial b_{GC,spline}}{\partial s_{\tilde{j}}}\right\}\end{aligned}$$

For the exponential version:

$$b^N_{GC} = Ne^{b_{GC,spline}}$$

$$\frac{\partial b_{GC}^{N}}{\partial b_{GC,spline}} = b_{GC}^{N}$$

For the inverse logit version:

$$b_{GC}^{N} = \frac{2N}{1 + e^{-b_{GC,spline}}}$$

$$\begin{aligned}
\frac{\partial b_{GC}^{N}}{\partial b_{GC,spline}} &= -2N \frac{-e^{-b_{GC,spline}}}{\left(1 + e^{-b_{GC,spline}}\right)^2} \\
&= b_{GC}^{N} \frac{e^{-b_{GC,spline}}}{1 + e^{-b_{GC,spline}}} \\
&= b_{GC}^{N} \frac{1}{1 + e^{b_{GC,spline}}}
\end{aligned}$$

Step 3 Negative binomial

$$L_{NB} = \prod_{n} \left\{ \binom{k_n + r_n - 1}{k_n} \left(1 - \frac{\mu_n}{\mu_n + r_n}\right)^{r_n} \left(\frac{\mu_n}{\mu_n + r_n}\right)^{k_n} \right\}$$

$$r_n = \frac{\mu_n}{\alpha + \beta\mu_n}$$

$$\begin{aligned}
\frac{\partial r_n}{\partial \mu_n} &= \frac{\alpha + \beta\mu_n - \mu_n\beta}{\left(\alpha + \beta\mu\right)^2} \\
&= \frac{\alpha}{\left(\alpha + \beta\mu\right)^2} \\
&= \frac{\alpha r_n^2}{\mu_n^2}
\end{aligned}$$

$$\begin{aligned}
\log(L_{NB}) &= \sum_{n} \left\{ \log\left(\prod_{i=1}^{k_n} \frac{k_n + r_n - 1 + 1 - i}{i} \right) + r_n \log\left(1 - \frac{\mu_n}{\mu_n + r_n}\right) + k_n \log\left(\frac{\mu_n}{\mu_n + r_n}\right) \right\} \\
&= \sum_{n} \left\{ \left[\sum_{i=1}^{k_n} \log\left(\frac{k_n + r_n - i}{i}\right) \right] + r_n \log\left(\frac{r_n}{\mu_n + r_n}\right) + k_n \log\left(\frac{\mu_n}{\mu_n + r_n}\right) \right\} \\
&= \sum_{n} \left\{ \left[\sum_{i=1}^{k_n} \log\left(k_n + r_n - (k_n - i + 1)\right) \right] - \left[\sum_{i=1}^{k_n} \log(i) \right] + r_n \log\left(\frac{r_n}{\mu_n + r_n}\right) + k_n \log\left(\frac{\mu_n}{\mu_n + r_n}\right) \right\} \\
&= \sum_{n} \left\{ \left[\sum_{i=1}^{k_n} \log\left(\frac{r_n + i - 1}{i}\right) \right] + r_n \log\left(\frac{r_n}{\mu_n + r_n}\right) + k_n \log\left(\frac{\mu_n}{\mu_n + r_n}\right) \right\} \\
&= \sum_{n} \left\{ k_n \log\left(\frac{\mu_n}{\mu_n + r_n}\right) + r_n \log\left(\frac{r_n}{\mu_n + r_n}\right) + \left[\sum_{i=1}^{k_n} \log\left(\frac{r_n + (i-1)}{i}\right) \right] \right\}
\end{aligned}$$

$$
\begin{aligned}
\frac{\partial \log(L_{NB})}{\partial \mu_n} = \quad & \sum_n \Bigg\{ \quad \left[k_n \frac{\mu_n + r_n}{\mu_n} \frac{(\mu_n + r_n) - \mu_n \left(1 + \frac{\partial r_n}{\partial \mu_n}\right)}{(\mu_n + r_n)^2} \right] \\
& + \left[\frac{\partial r_n}{\partial \mu_n} \log\left(\frac{r_n}{\mu_n + r_n}\right) + r_n \frac{\mu_n + r_n}{r_n} \frac{\frac{\partial r_n}{\partial \mu_n}(\mu_n + r_n) - r_n \left(1 + \frac{\partial r_n}{\partial \mu_n}\right)}{(\mu_n + r_n)^2} \right] \\
& + \left[\sum_{i=1}^{k_n} \frac{i}{r_n + i - 1} \frac{\frac{\partial r_n}{\partial \mu_n}}{i} \right] \Bigg\} \\
= \quad & \sum_n \Bigg\{ \quad \left[k_n \frac{r_n - \mu_n \frac{\partial r_n}{\partial \mu_n}}{\mu_n(\mu_n + r_n)} \right] + \left[\frac{\mu_n \frac{\partial r_n}{\partial \mu_n} - r_n}{\mu_n + r_n} + \frac{\partial r_n}{\partial \mu_n} \log\left(\frac{r_n}{\mu_n + r_n}\right) \right] + \left[\sum_{i=1}^{k_n} \frac{\frac{\partial r_n}{\partial \mu_n}}{r_n + i - 1} \right] \Bigg\} \\
= \quad & \sum_n \Bigg\{ \quad \frac{(k_n - \mu_n)\left(r_n - \mu_n \frac{\partial r_n}{\partial \mu_n}\right)}{\mu_n(\mu_n + r_n)} + \frac{\partial r_n}{\partial \mu_n} \log\left(\frac{r_n}{\mu_n + r_n}\right) + \left[\frac{\partial r_n}{\partial \mu_n} \sum_{i=1}^{k_n} \frac{1}{r_n + i - 1} \right] \Bigg\} \\
= \quad & \sum_n \Bigg\{ \quad \frac{(k_n - \mu_n)\left(r_n - \mu_n \frac{\alpha r_n^2}{\mu_n^2}\right)}{\mu_n(\mu_n + r_n)} + \frac{\alpha r_n^2}{\mu_n^2} \log\left(\frac{r_n}{\mu_n + r_n}\right) + \left[\frac{\alpha r_n^2}{\mu_n^2} \sum_{i=1}^{k_n} \frac{1}{r_n + i - 1} \right] \Bigg\} \\
= \quad & \sum_n \Bigg\{ \quad \frac{r_n(k_n - \mu_n)}{\mu_n(\mu_n + r_n)} \left(1 - \frac{\alpha r_n}{\mu_n}\right) + \left[\log\left(\frac{r_n}{\mu_n + r_n}\right) + \sum_{i=1}^{k_n} \frac{1}{r_n + i - 1} \right] r_n^2 \frac{\alpha}{\mu_n^2} \Bigg\} \\
= \quad & \sum_n \Bigg\{ \quad \left(\frac{r_n(k_n - \mu_n)}{\mu_n + r_n} \left(1 - \frac{\alpha r_n}{\mu_n}\right) + \left[\log\left(\frac{1}{\frac{\mu_n}{r_n} + 1}\right) + \sum_{i=1}^{k_n} \frac{1}{r_n + (i-1)} \right] r_n^2 \frac{\alpha}{\mu_n} \right) \frac{1}{\mu_n} \Bigg\}
\end{aligned}
$$

$$
\begin{aligned}
\frac{\partial \log(L_{NB})}{\partial s_{\tilde{j}}} \quad = \quad & \frac{\partial \log(L_B)}{\partial \mu_n} \frac{\partial \mu_n}{\partial b_{GC}^N} \frac{\partial b_{GC}^N}{\partial b_{GC,spline}} \frac{\partial b_{GC,spline}}{\partial s_{\tilde{j}}} \\
= \quad & \left[\frac{\partial \log(L_B)}{\partial \mu_n} \mu_n \right] \left[\frac{1}{b_{GC}^N} \frac{\partial b_{GC}^N}{\partial b_{GC,spline}} \right] \frac{\partial b_{GC,spline}}{\partial s_{\tilde{j}}}
\end{aligned}
$$

$$
\frac{\partial \log(L_{NB})}{\partial b_{f,p}} = \left[\frac{\partial \log(L_B)}{\partial \mu_n} \mu_n \right] \left[\frac{1}{\mu_n} \frac{\partial \mu_n}{\partial b_{f,p}} \right]
$$

$$
\begin{aligned}
\frac{\partial \log(L_{NB})}{\partial r_n} = \quad & \sum_n \Bigg\{ \quad \left[k_n \frac{\mu_n + r_n}{\mu_n} \frac{-\mu_n}{(\mu_n + r_n)^2} \right] \\
& + \left[\log\left(\frac{r_n}{\mu_n + r_n}\right) + r_n \frac{\mu_n + r_n}{r_n} \frac{(\mu_n + r_n) - r_n}{(\mu_n + r_n)^2} \right] \\
& + \left[\sum_{i=1}^{k_n} \frac{i}{r_n + i - 1} \frac{1}{i} \right] \Bigg\} \\
= \quad & \sum_n \Bigg\{ \quad \left[\frac{-k_n}{\mu_n + r_n} \right] + \left[\frac{\mu_n}{\mu_n + r_n} + \log\left(\frac{r_n}{\mu_n + r_n}\right) \right] + \left[\sum_{i=1}^{k_n} \frac{1}{r_n + i - 1} \right] \Bigg\} \\
= \quad & \sum_n \Bigg\{ \quad \frac{\mu_n - k_n}{\mu_n + r_n} + \log\left(\frac{1}{\frac{\mu_n}{r_n} + 1}\right) + \left[\sum_{i=1}^{k_n} \frac{1}{r_n + (i-1)} \right] \Bigg\}
\end{aligned}
$$

$$
\frac{\partial r_n}{\partial \alpha} = \frac{-\mu_n}{(\alpha + \beta\mu)^2} = \frac{-r_n^2}{\mu_n}
$$

$$
\begin{aligned}
\frac{\partial \log(L_{NB})}{\partial \alpha} &= \frac{\partial \log(L_B)}{\partial r_n} \frac{\partial r_n}{\partial \alpha} \\
&= \left\{ \frac{r_n^2}{\mu_n} \frac{k_n - \mu_n}{\mu_n + r_n} - \left[\log\left(\frac{1}{\frac{\mu_n}{r_n} + 1} \right) + \sum_{i=1}^{k_n} \frac{1}{r_n + (i-1)} \right] \frac{r_n^2}{\mu_n} \right\}
\end{aligned}
$$

$$
\frac{\partial r_n}{\partial \beta} = \frac{-\mu_n^2}{(\alpha + \beta\mu)^2} = -r_n^2
$$

$$
\begin{aligned}
\frac{\partial \log(L_{NB})}{\partial \beta} &= \frac{\partial \log(L_B)}{\partial r_n} \frac{\partial r_n}{\partial \beta} \\
&= \left\{ r_n^2 \frac{k_n - \mu_n}{\mu_n + r_n} - \left[\log\left(\frac{1}{\frac{\mu_n}{r_n} + 1} \right) + \sum_{i=1}^{k_n} \frac{1}{r_n + (i-1)} \right] r_n^2 \right\}
\end{aligned}
$$

References

1. Wingett, S.: Illumina Patterned Flow Cells Generate Duplicated Sequences. `https://sequencing.qcfail.com/articles/illumina-patterned-flow-cells-generate-duplicated-sequences/` Accessed 2021-06-01
2. Li, H.: A statistical framework for SNP calling, mutation discovery, association mapping and population genetical parameter estimation from sequencing data. Bioinformatics **27**(21), 2987–2993 (2011)
3. Quinlan, A.R., Hall, I.M.: BEDTools: a flexible suite of utilities for comparing genomic features. Bioinformatics **26**(6), 841–842 (2010)
4. Langmead, B., Salzberg, S.L.: Fast gapped-read alignment with Bowtie 2. Nat. Methods **9**(4), 357–359 (2012)
5. H., L.: Aligning sequence reads, clone sequences and assembly contigs with bwa-mem. arXiv **1303.3997v2** (2013)
6. Garrison E, M.G.: Haplotype-based variant detection from short-read sequencing. arXiv **1207.3907** (2012)
7. Robinson, J.T., Thorvaldsdóttir, H., Winckler, W., Guttman, M., Lander, E.S., Getz, G., Mesirov, J.P.: Integrative genomics viewer. Nature Biotechnology **29**, 24–26 (2011)
8. Thorvaldsdóttir, H., Robinson, J.T., Mesirov, J.P.: Integrative genomics viewer (igv): high-performance genomics data visualization and exploration. Briefings in Bioinformatics **14**, 178–192 (2013)
9. Marçais, G., Kingsford, C.: A fast, lock-free approach for efficient parallel counting of occurrences of k-mers. Bioinformatics **27**(6), 764–770 (2011)
10. Kokot, M., Dlugosz, M., Deorowicz, S.: KMC 3: counting and manipulating k-mer statistics. Bioinformatics **33**(17), 2759–2761 (2017)
11. Walker, B.J., Abeel, T., Shea, T., Priest, M., Abouelliel, A., Sakthikumar, S., Cuomo, C.A., Zeng, Q., Wortman, J., Young, S.K., Earl, A.M.: Pilon: an integrated tool for comprehensive microbial variant detection and genome assembly improvement. PLoS ONE **9**(11), 112963 (2014)
12. Simpson, J.T.: Exploring genome characteristics and sequence quality without a reference. Bioinformatics **30**(9), 1228–1235 (2014)
13. Gurevich, A., Saveliev, V., Vyahhi, N., Tesler, G.: QUAST: quality assessment tool for genome assemblies. Bioinformatics **29**(8), 1072–1075 (2013)
14. Li, H., Handsaker, B., Wysoker, A., Fennell, T., Ruan, J., Homer, N., Marth, G., Abecasis, G., Durbin, R.: The Sequence Alignment/Map format and SAMtools. Bioinformatics **25**(16), 2078–2079 (2009)
15. Simpson, J.T., Durbin, R.: Efficient de novo assembly of large genomes using compressed data structures. Genome Res. **22**(3), 549–556 (2012)
16. Köster, J., Rahmann, S.: Snakemake–a scalable bioinformatics workflow engine. Bioinformatics **28**(19), 2520–2522 (2012)
17. Li, R., Yu, C., Li, Y., Lam, T.W., Yiu, S.M., Kristiansen, K., Wang, J.: SOAP2: an improved ultrafast tool for short read alignment. Bioinformatics **25**(15), 1966–1967 (2009)
