# Supplementary material for: ReSeq simulates realistic Illumina high-throughput sequencing data
Source: Genome Biol. 2021 Feb 19;22:67. doi: 10.1186/s13059-021-02265-7 (PMC7896392; doi:10.1186/s13059-021-02265-7)
Supplement: Supplementary file 2 — Additional file 2 QUAST assembly report. [file 13059_2021_2265_MOESM2_ESM.pdf]

Report

	sga-contigs
# contigs (>= 0 bp)	61992
# contigs (>= 1000 bp)	1872
# contigs (>= 5000 bp)	306
# contigs (>= 10000 bp)	137
# contigs (>= 25000 bp)	14
# contigs (>= 50000 bp)	0
Total length (>= 0 bp)	16229865
Total length (>= 1000 bp)	6216155
Total length (>= 5000 bp)	3477799
Total length (>= 10000 bp)	2265808
Total length (>= 25000 bp)	465690
Total length (>= 50000 bp)	0
# contigs	61992
Largest contig	47116
Total length	16229865
Reference length	4616724
GC (%)	48.23
Reference GC (%)	50.80
N50	402
NG50	9781
N75	127
NG75	5017
L50	4813
LG50	142
L75	29805
LG75	303
# misassemblies	3
# misassembled contigs	3
Misassembled contigs length	681
# local misassemblies	4
# unaligned mis. contigs	0
# unaligned contigs	8053 + 0 part
Unaligned length	4567968
Genome fraction (%)	98.928
Duplication ratio	2.553
# N's per 100 kbp	0.00
# mismatches per 100 kbp	6.59
# indels per 100 kbp	0.26
Largest alignment	47116
Total aligned length	11640635
NA50	126
NGA50	9781
NGA75	5017
LA50	25821
LGA50	142
LGA75	303

Misassemblies report

	sga-contigs
# misassemblies	3
# relocations	3
# translocations	0
# inversions	0
# misassembled contigs	3
Misassembled contigs length	681
# local misassemblies	4
# unaligned mis. contigs	0
# mismatches	301
# indels	12
# indels (<= 5 bp)	12
# indels (> 5 bp)	0
Indels length	12

Unaligned report

	sga-contigs
# fully unaligned contigs	8053
Fully unaligned length	4567968
# partially unaligned contigs	0
Partially unaligned length	0
# N's	0

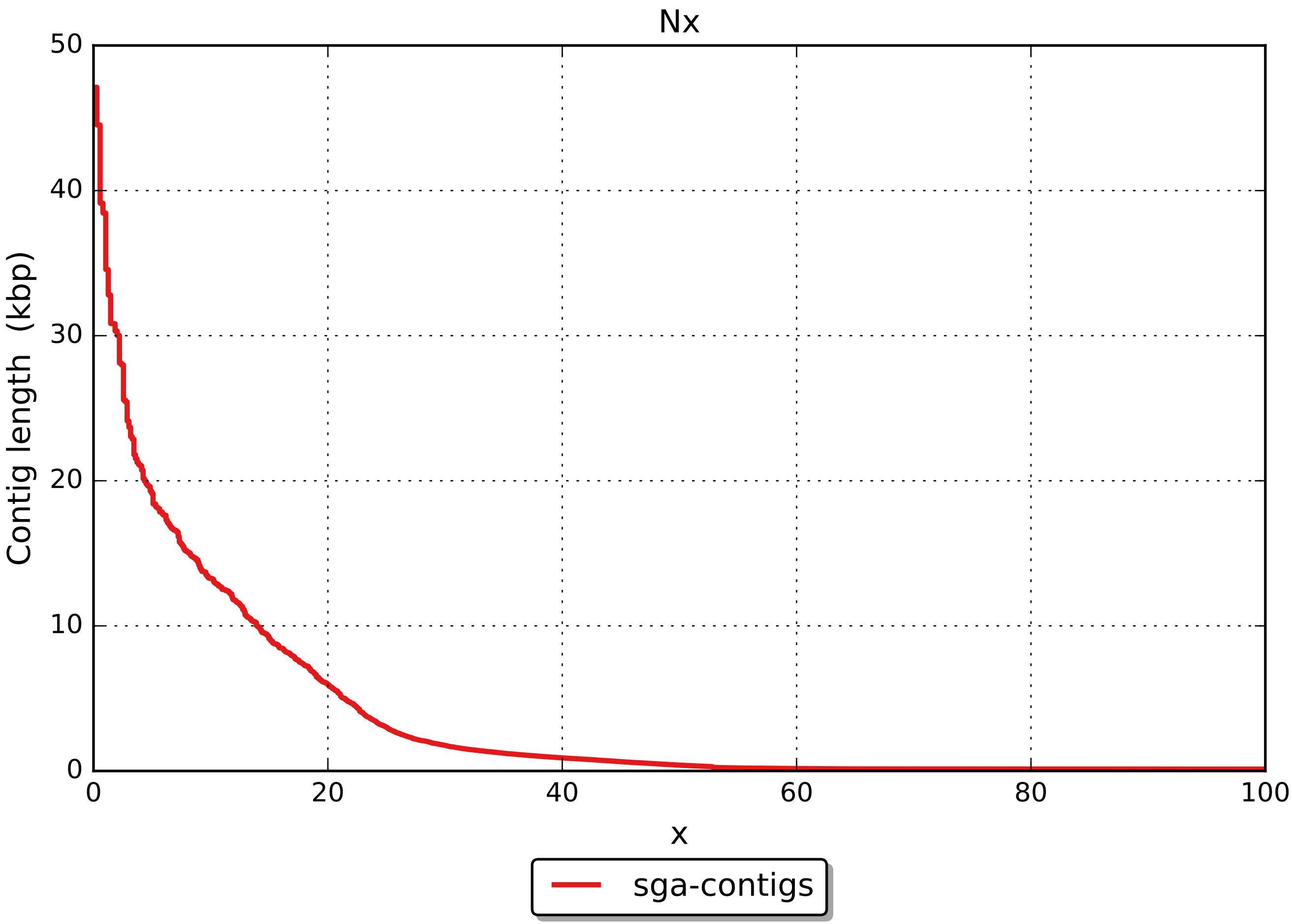
Nx
Contig length (kbp)
x
0
10
20
30
40
50
0
20
40
60
80
100
sga-contigs

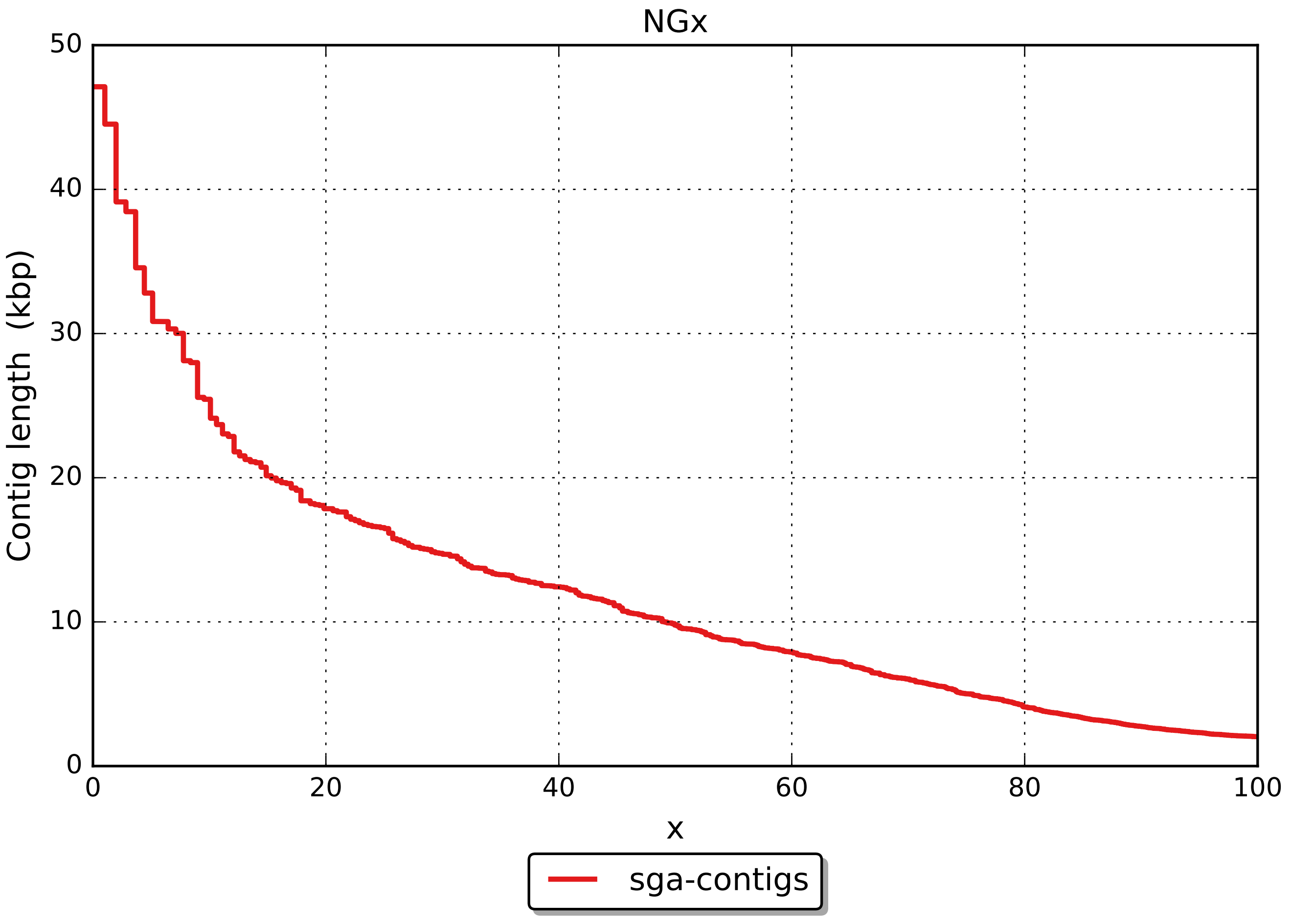
NGx
Contig length (kbp)
0
10
20
30
40
50
0
20
40
60
80
100
x
sga-contigs

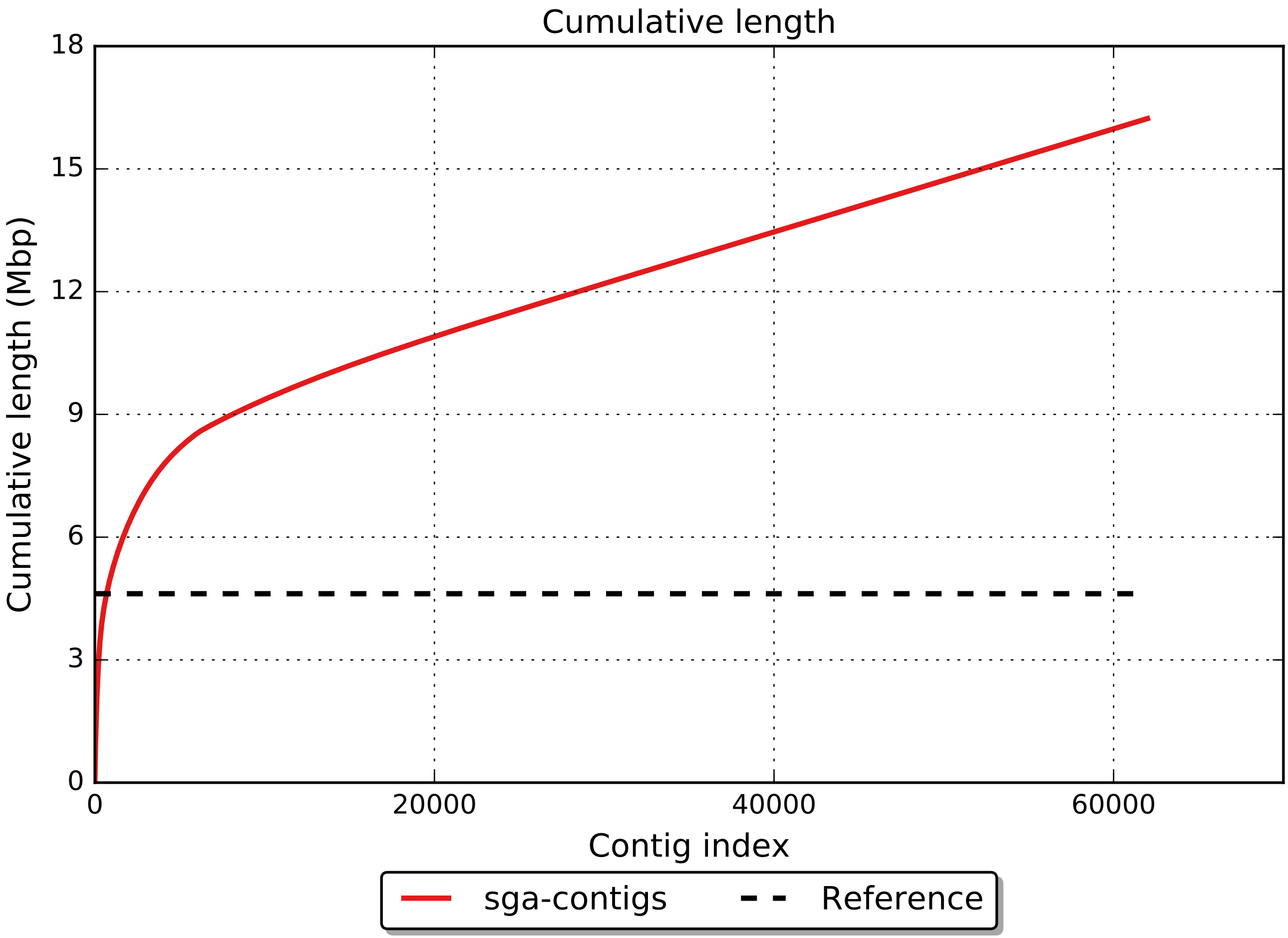

Cumulative length
Cumulative length (Mbp)
0
3
6
9
12
15
18
0
20000
40000
60000
Contig index
sga-contigs
Reference

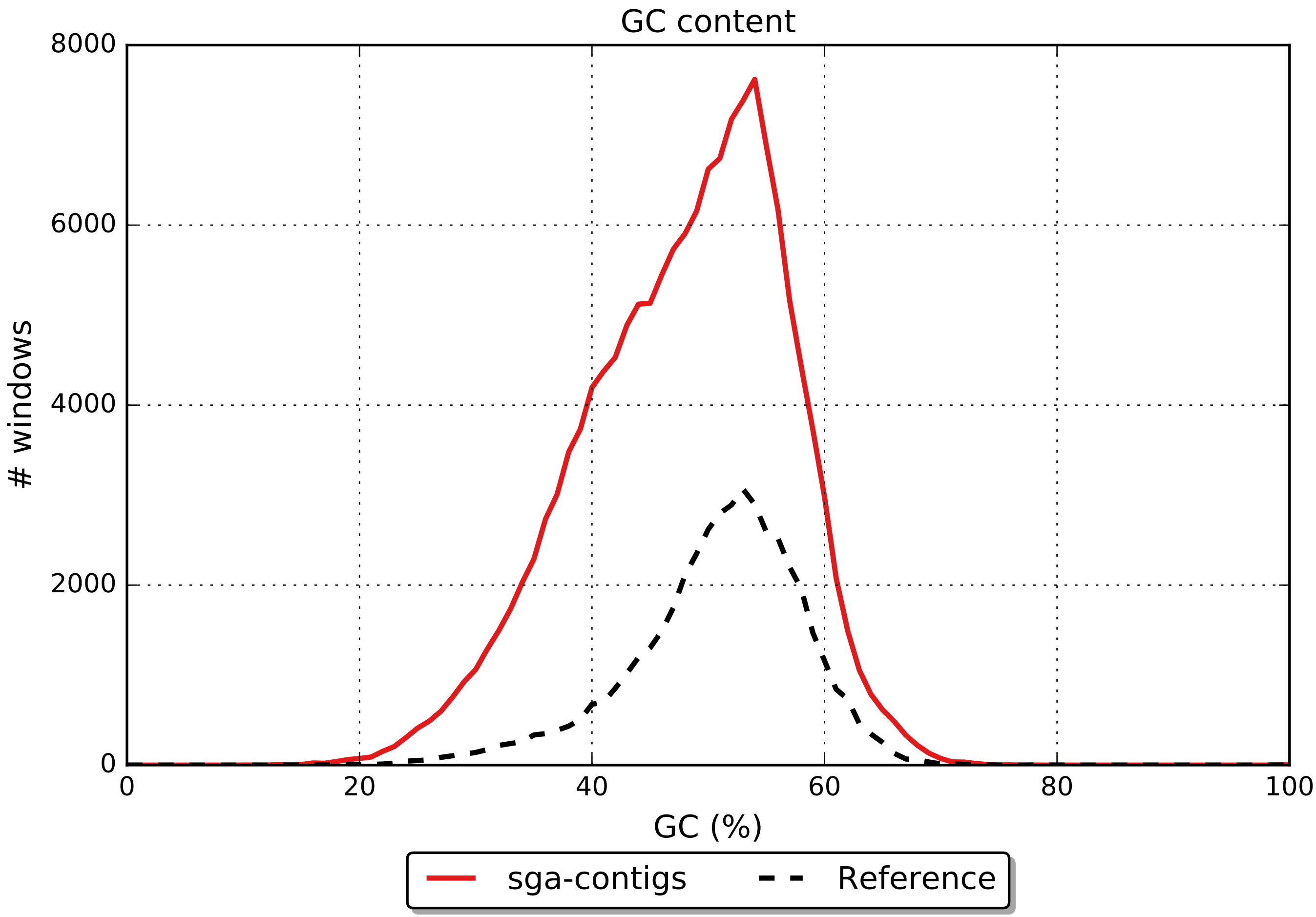
GC content
8000
6000
4000
2000
0
0
20
40
60
80
100
windows
GC (%)
sga-contigs
Reference

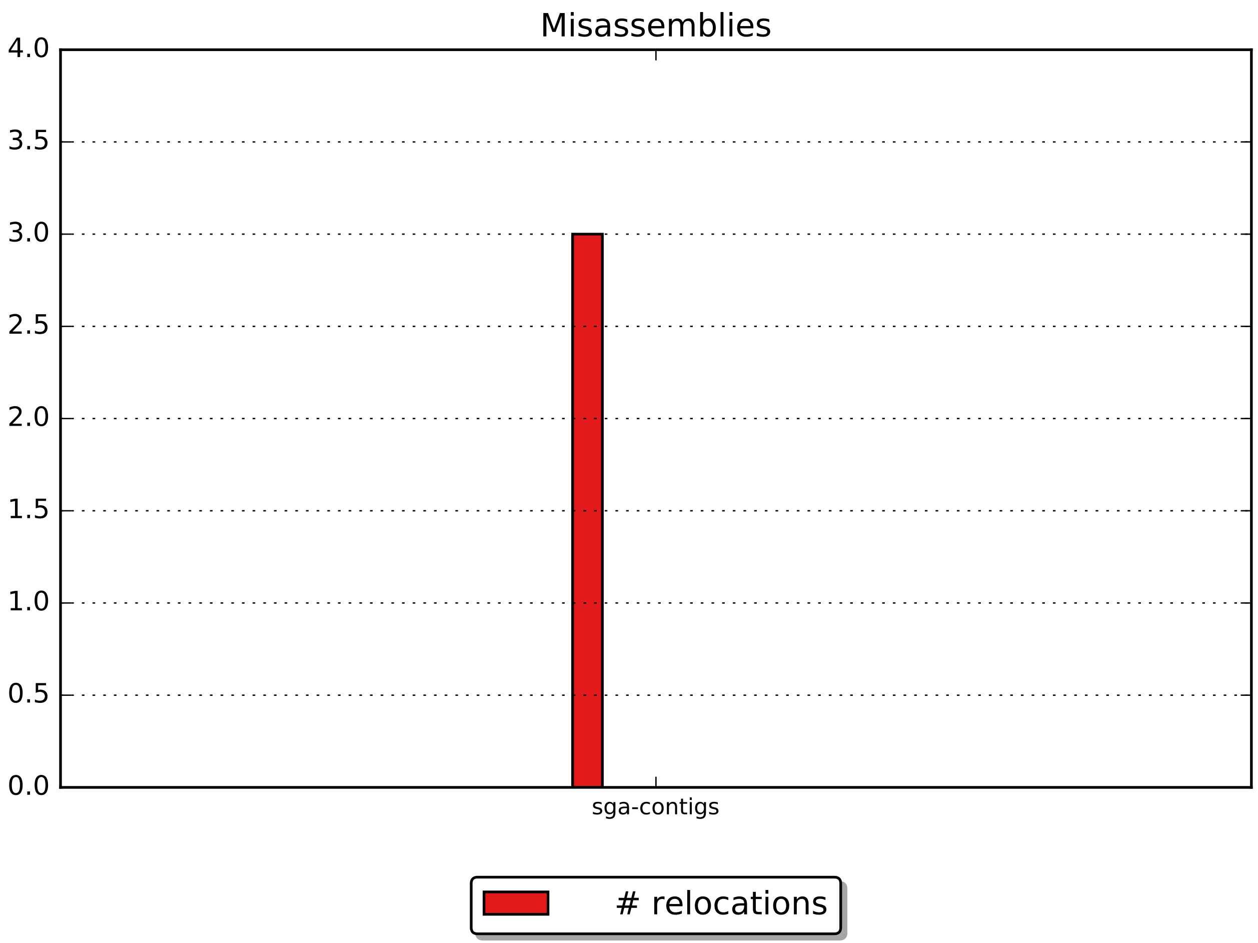

Misassemblies
4.0
3.5
3.0
2.5
2.0
1.5
1.0
0.5
0.0
sga-contigs
relocations

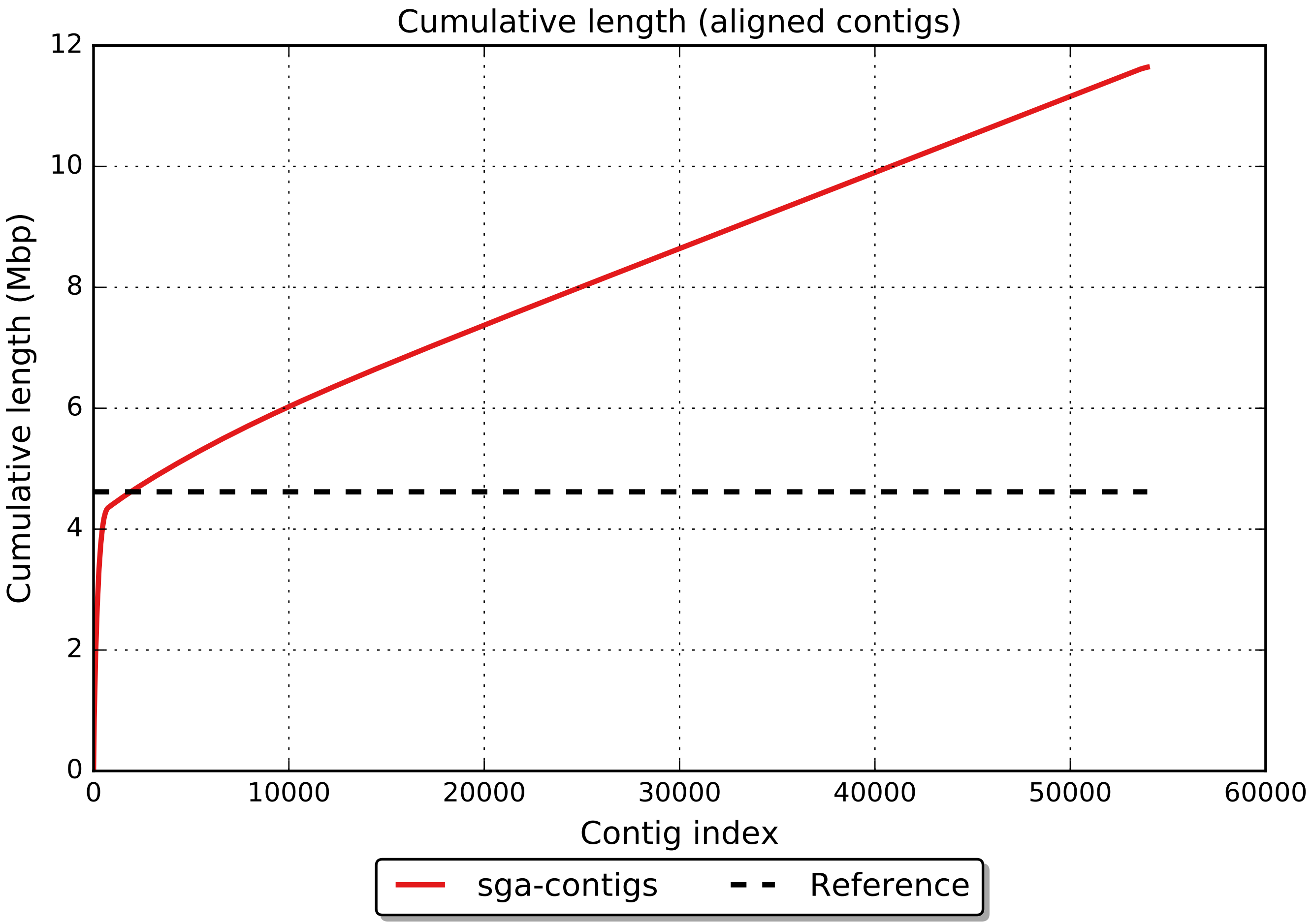

Cumulative length (aligned contigs)
Cumulative length (Mbp)
0
2
4
6
8
10
12
0
10000
20000
30000
40000
50000
60000
Contig index
sga-contigs
Reference

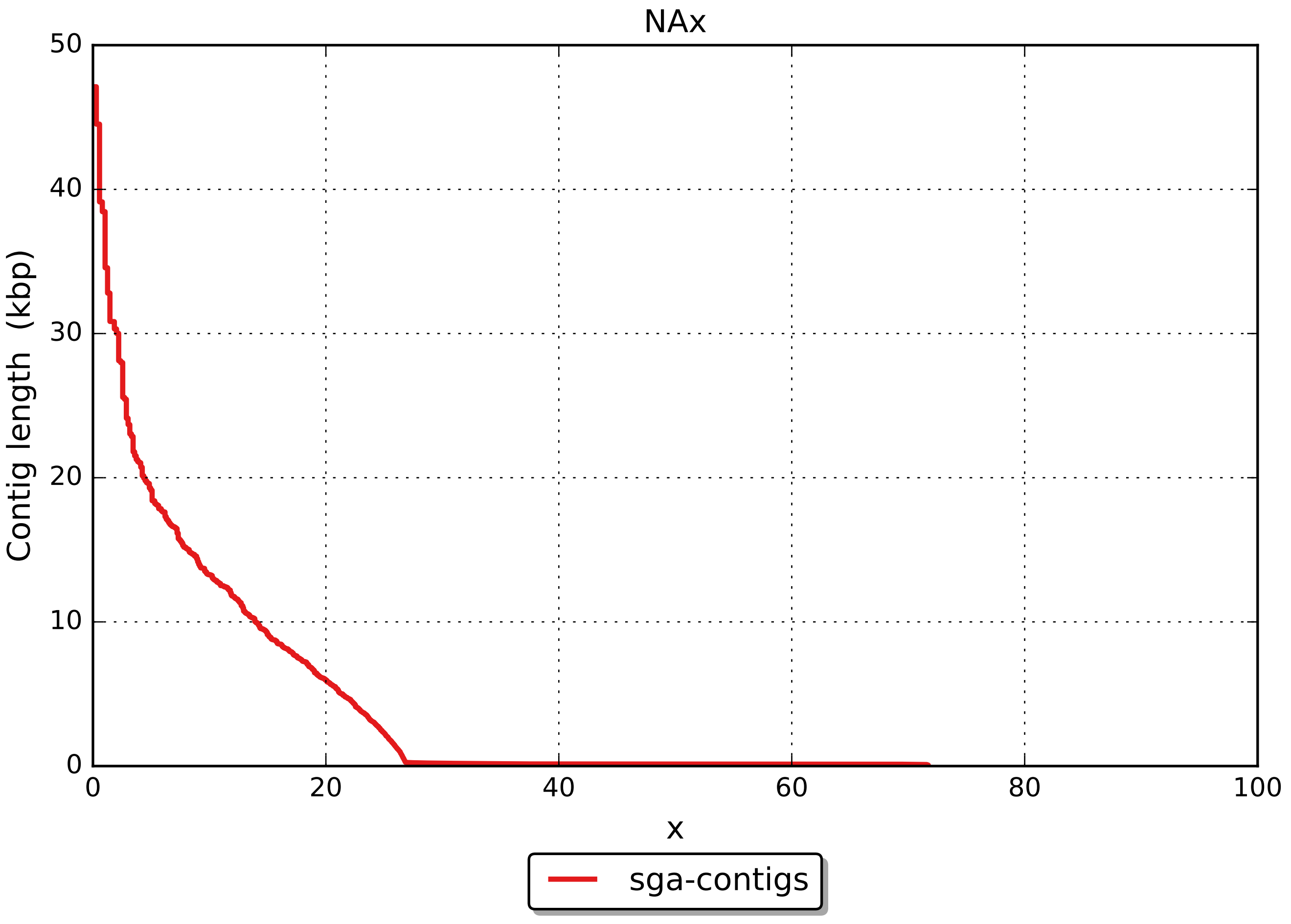

NAx
Contig length (kbp)
x
0
10
20
30
40
50
0
20
40
60
80
100
sga-contigs

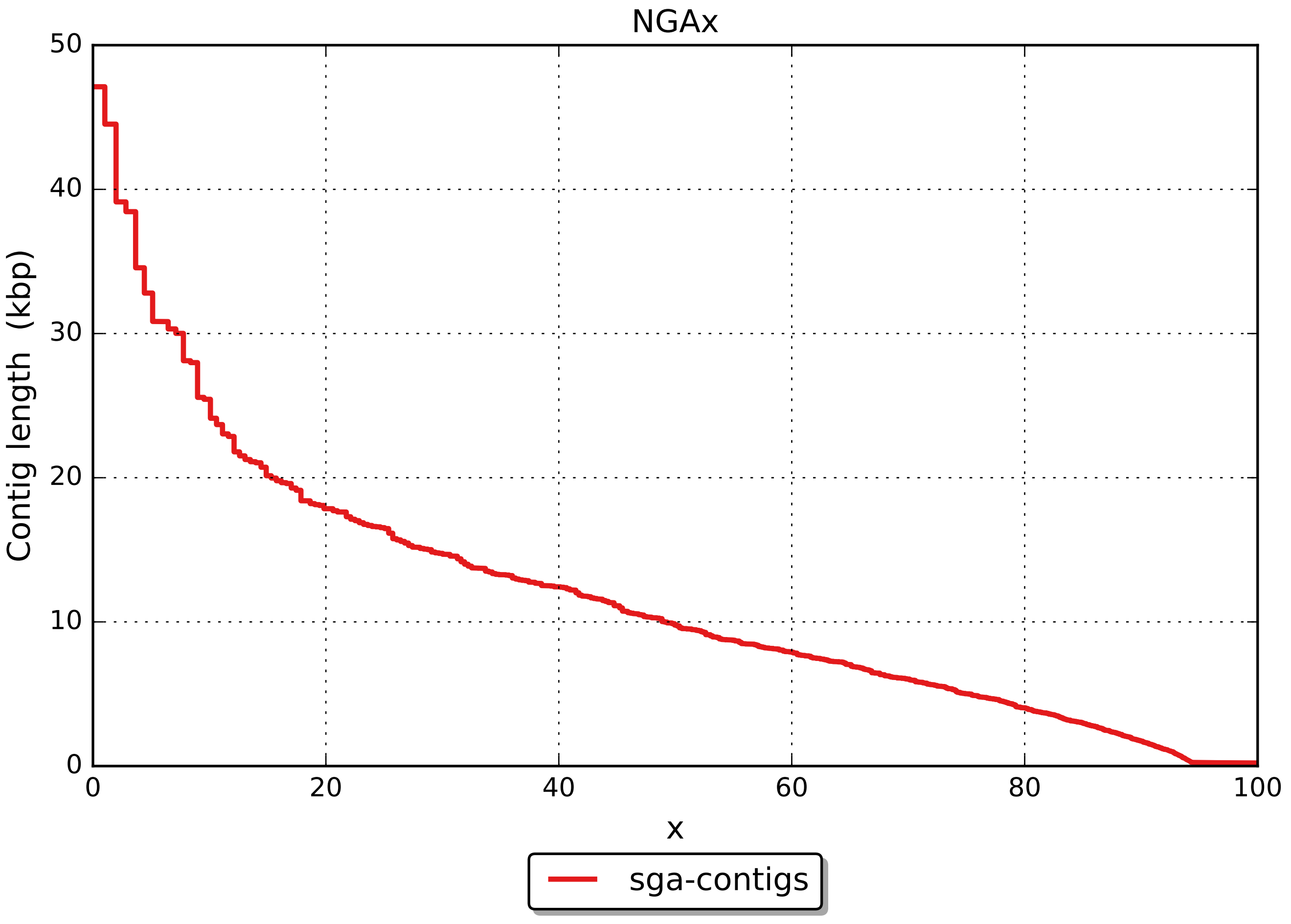
NGAx
Contig length (kbp)
0
10
20
30
40
50
0
20
40
60
80
100
x
sga-contigs
