# Supplementary material for: ReSeq simulates realistic Illumina high-throughput sequencing data
Source: Genome Biol. 2021 Feb 19;22:67. doi: 10.1186/s13059-021-02265-7 (PMC7896392; doi:10.1186/s13059-021-02265-7)
Supplement: Supplementary file 4 — Additional file 4 Review history. [file 13059_2021_2265_MOESM4_ESM.docx]

Review History

**First round of review**

**Reviewer 1**

**Were you able to assess all statistics in the manuscript, including the appropriateness of statistical tests used?**

No

**Were you able to directly test the methods?**

No

**Comments to author:**

This manuscript presents a new method for simulating the Illumina Sequencing reads. Although the authors have made some improvements, this is a very old topic. There are already several available software that can fullfill the purpose, which published many years before (~8-10 years).

The future may turn more to the third generation and fourth generation sequencing technologies, such as Pacbio, nanopore. Even for the second generation technology, Illumina will not be the only one, as I know, BGI's MGISEQ is a strong competitor. Whether the method developed here can be applied to BGI's MGISEQ, and other sequencing technologies?

The manuscript is long, and the logic is not so clear, so it may be hard to read for common readers. I suggest to make a concise manuscript.

**Reviewer 2**

**Were you able to assess all statistics in the manuscript, including the appropriateness of statistical tests used?**

No

**Were you able to directly test the methods?**

Yes

**Comments to author:**

Schmeing and Robinson developed a sophisticated new method called ReSeq for generating genome-wide simulation read data. Generating highly accurate simulation data that mimic the real sequencing data is important for benchmarking bioinformatics methods and for developing new methods.

However, the new method ReSeq developed in this study does not seem to clearly outperform the other methods being compared. For example, in the comparison results shown in Table 3, the method pIRS actually has a better performance than ReSeq. For the rest of comparison results presented in the manuscript, although ReSeq showed advantage in some of the comparisons, there are quite a few comparisons in which it is difficult to tell the difference between ReSeq and other methods or ReSeq performed slightly worse than some other methods.

ReSeq also has a large computational cost. It can use multiple CPUs to reduce running time, but this comes at a heavy memory cost of up to 200GB. Therefore ReSeq does not seem to have an advantage in computational efficiency compared to other methods.

This reviewer also has some concerns about the ReSeq algorithm. ReSeq uses mapped reads to train its model and then generates simulation reads. However the mapped reads usually contain a non-negligible amount of soft-clipped bases, which are reported by most read aligners such as Bowtie2 to indicate those bases that failed to be mapped along with other bases in the same read. These soft-clipped bases are known to include low-sequencing-quality bases, which are important for simulators to generate data similar to the real data. However the soft-clipped bases seem to ignored by ReSeq which may result in inaccurate modelling of the real data.

Has the mapping error been considered by ReSeq when it tries to capture the characteristics of the real data? There are certainly mapping errors in the mapping result reported by any aligner including Bowtie2. The mapping errors need to be identified and corrected before error profile of the real data can be estimated.

How does ReSeq deal with repetitive regions in the genome? Generating simulation reads for repetitive regions, in addition to other regions in the genome is important for assessing the performance of many bioinformatics tools.

How does ReSeq incorporate adapter sequences into its statistics matrices? The schematic in Figure 1 shows that adapter sequences are provided to ReSeq. However, in the Discussion section the authors stated that the adapters are learned from the data. This needs to be clarified.

**Reviewer 3**

**Were you able to assess all statistics in the manuscript, including the appropriateness of statistical tests used?**

No

**Were you able to directly test the methods?**

Yes

**Comments to author:**

The authors proposed a new approach (named as ReSeq) to the simulation of short read sequencing data, especially for Illumina platforms. This is a quite important topic since realistic simulated data has wide applications in the development of bioinformatics tools. There have been quite a lot of such simulators proposed in recent years, however, the authors claim that ReSeq improves the authenticity of synthetic data. Overall, the proposed method is benchmarked in various aspects with several datasets, and manuscript is well-prepared. However, I still have some concerns which should be addressed.

1. I think some parts of the Results section need to be revised. There are many implementation details given in this section, mixed with benchmark results. This makes Results section very long, moreover, not very clear and a little hard to read. I suggest to give some brief introductions of the technical details of ReSeq method in Results section, and focus more on benchmark results. Then, give more design and implementation details in Methods section.

2. There are 8 real sequencing datasets used in Results, which are from various Illumina sequencing platforms and species. However, the platforms seem a little out-of-date, for example, there is no data from the newest Illumina Novaseq platform. Considering that Novaseq has being used in many large-scale genomics studies, and it also has its own characteristics, I suggest to add some benchmarks with real Novaseq datasets in Results.

3. In Figures 2-4, why there are only results for Hiseq 2000/4000 datasets. I think the results of other benchmarked datasets should be also added.

4. The authors claimed that k-mer spectrum is important to investigate the systematic properties of errors and coverage distribution. But their relationships are not very clear. It is better to add more clear explanations. Moreover, why only 51-mer spectrum is used? As it is very important, more results with various k parameter settings should be added.

5. It is very important for short read simulators to demonstrate their ability of cross-species simulations. The authors give the comparisons of ReSeq and other simulators in this aspect (Figure 7). However, only the comparisons on k-mer spectrum. I think this is not enough, other statistics used in Results section should be also added to make a more comprehensive benchmark.

6. The speed of simulation is also important, especially when large-scale simulation datasets are needed. The authors give the comparison of speed as well as memory usage (Figure 8), and claimed that ReSeq has effective parallelization, so that it has lower time cost (wall-time). This is a very good feature, however, I also noticed that the memory footprint of ReSeq is quite high in simulation, even an order of magnitude higher than some tools, such as ART and pIRS. So, I would like to know what will happen if all the tools are run in a multi-process way instead of multi-threading, say, the tools are run in a single thread, and many entities are run simultaneously. Since the memory footprints of some simulators are lower, I think a machine can afford many entities of them, and the total throughput could be higher than that of multi-threading mode.

**Authors Response**

**Point-by-point responses to the reviewers’ comments:**

*Extra revisions unrelated to the Reviewers’ comments:*

*- We trimmed the abstract to 100 words.*

*- We updated all results for version 1.1 of ReSeq and changed the text accordingly*

Reviewer #1: This manuscript presents a new method for simulating the Illumina Sequencing reads. Although the authors have made some improvements, this is a very old topic. There are already several available software that can fullfill the purpose, which published many years before (~8-10 years). The future may turn more to the third generation and fourth generation sequencing technologies, such as Pacbio, nanopore. Even for the second generation technology, Illumina will not be the only one, as I know, BGI's MGISEQ is a strong competitor. Whether the method developed here can be applied to BGI's MGISEQ, and other sequencing technologies?

*- Illumina is still the most widely used technology today and will likely stay an important technology in the near future, due to the costs of third generation sequencing and yet unresolved issues as the high error rates in nanopore data or the length-dependent bias in transcriptome data for PacBio. Furthermore, individual parts of ReSeq are not Illumina-specific. For example, the coverage model might also be suitable for third generation sequencing, but investigating this lies outside the scope of this paper.*

*- The applicability to BGISEQ is indeed an interesting question and we added a BGISEQ-500 dataset. The results suggest that ReSeq can also simulate this related technology.*

The manuscript is long, and the logic is not so clear, so it may be hard to read for common readers. I suggest to make a concise manuscript.

*- We do believe that it is important to raise awareness for generating high-quality simulations, the involved methods and the necessity of excessive tests. Thus, we followed the more detailed advice of reviewer 3 on how to improve the flow and readability of the paper.*

Reviewer #2: Schmeing and Robinson developed a sophisticated new method called ReSeq for generating genome-wide simulation read data. Generating highly accurate simulation data that mimic the real sequencing data is important for benchmarking bioinformatics methods and for developing new methods.

However, the new method ReSeq developed in this study does not seem to clearly outperform the other methods being compared. For example, in the comparison results shown in Table 3, the method pIRS actually has a better performance than ReSeq. For the rest of comparison results presented in the manuscript, although ReSeq showed advantage in some of the comparisons, there are quite a few comparisons in which it is difficult to tell the difference between ReSeq and other methods or ReSeq performed slightly worse than some other methods.

*- It is true that ReSeq does not outperform all other methods in every single test, but overall still gives a substantial improvement in simulation quality. To better highlight this, we included a summary figure of the results shown throughout the paper at the beginning of Discussion.*

*- The specific example of Table 3 seems to be a misconception. The correlation of ReSeq with the real data clearly outperforms any other simulator and for the correlation of simulations with itself, the aim is not to have the highest correlation, but to reach the correlation between real replicates. Thus, ReSeq and pIRS diverge from the optimum in other directions and we do think that slightly overestimating the randomness is better than removing it almost completely as pIRS does. To demonstrate more clearly that the aim is to achieve the correlation of real data, we marked the best entries bold.*

ReSeq also has a large computational cost. It can use multiple CPUs to reduce running time, but this comes at a heavy memory cost of up to 200GB. Therefore ReSeq does not seem to have an advantage in computational efficiency compared to other methods.

*- We added a memory-minimizing setup for all datasets to the computational comparison, showing that the memory cost can be drastically reduced (if not enough memory is available). By choosing the number of threads, the optimal tradeoff between memory usage and speed can be achieved, which is not possible for single-threaded applications. Although this is not increasing computational efficiency, we consider it a clear computational advantage.*

This reviewer also has some concerns about the ReSeq algorithm. ReSeq uses mapped reads to train its model and then generates simulation reads. However the mapped reads usually contain a non-negligible amount of soft-clipped bases, which are reported by most read aligners such as Bowtie2 to indicate those bases that failed to be mapped along with other bases in the same read. These soft-clipped bases are known to include low-sequencing-quality bases, which are important for simulators to generate data similar to the real data. However the soft-clipped bases seem to ignored by ReSeq which may result in inaccurate modelling of the real data.

*- We added a clarification in the Simulations subsection of Methods that we run bowtie2 in the default end-to-end alignment mode, which does not have soft-clipped bases. Under Stored probabilities and conditions in Methods, we added that in the case of soft clipping, we extend the read to its full length ignoring the rare case of InDels in this part.*

Has the mapping error been considered by ReSeq when it tries to capture the characteristics of the real data? There are certainly mapping errors in the mapping result reported by any aligner including Bowtie2. The mapping errors need to be identified and corrected before error profile of the real data can be estimated.

*- ReSeq has an option to set a minimum mapping quality. The default is 2, which leaves only a low amount of false mappings. Furthermore, some checks on the mapping consistency of pairs are performed and repeat regions that are prone to errors are filtered. ReSeq’s filters work well according to the tests on Ec-Hi2500-TruSeq-asm, where a short-read assembly instead of the reference is used; notably, ReSeq can handle this situation much better than other simulators.*

How does ReSeq deal with repetitive regions in the genome? Generating simulation reads for repetitive regions, in addition to other regions in the genome is important for assessing the performance of many bioinformatics tools.

*- Simulating from repetitive regions does not require any special considerations as long as they are part of the simulation template/reference. We clarified this in the Coverage model section of Results. For training, the repetitive regions are filtered out to reduce the amount of false mappings.*

How does ReSeq incorporate adapter sequences into its statistics matrices? The schematic in Figure 1 shows that adapter sequences are provided to ReSeq. However, in the Discussion section the authors stated that the adapters are learned from the data. This needs to be clarified.

*- In the figure and its caption, the adapters are marked as optional input and the text in Methods describes that the adapters can be specified to deal with cases where the automatic detection fails. We added a clarifying sentence in the Result section.*

Reviewer #3: The authors proposed a new approach (named as ReSeq) to the simulation of short read sequencing data, especially for Illumina platforms. This is a quite important topic since realistic simulated data has wide applications in the development of bioinformatics tools. There have been quite a lot of such simulators proposed in recent years, however, the authors claim that ReSeq improves the authenticity of synthetic data. Overall, the proposed method is benchmarked in various aspects with several datasets, and manuscript is well-prepared. However, I still have some concerns which should be addressed. 1. I think some parts of the Results section need to be revised. There are many implementation details given in this section, mixed with benchmark results. This makes Results section very long, moreover, not very clear and a little hard to read. I suggest to give some brief introductions of the technical details of ReSeq method in Results section, and focus more on benchmark results. Then, give more design and implementation details in Methods section.

*- We moved about one page of content from Results into Methods to improve readability. Furthermore, we added subsections to better separate ReSeq details from the benchmark results and moved Comparison of quality values and error rates to better bundle topics.*

2. There are 8 real sequencing datasets used in Results, which are from various Illumina sequencing platforms and species. However, the platforms seem a little out-of-date, for example, there is no data from the newest Illumina Novaseq platform. Considering that Novaseq has being used in many large-scale genomics studies, and it also has its own characteristics, I suggest to add some benchmarks with real Novaseq datasets in Results.

*- We added a NovaSeq and a MiSeq dataset to be more complete on the widely used sequencers.*

3. In Figures 2-4, why there are only results for Hiseq 2000/4000 datasets. I think the results of other benchmarked datasets should be also added.

*- Figure 2 is mostly for illustration purposes and would likely suffer from adding more content to it. Instead, we added an overview on systematic errors in all datasets as a Supplementary Figure.*

*- Figure 3 visualizes internal values and we do not see a clear benefit in showing them for all possible datasets. Instead, it would make the Figure much harder to read.*

*- We split Figure 4 into two Figures to show all datasets and also added a Supplementary Figure with fragment length for all datasets.*

4. The authors claimed that k-mer spectrum is important to investigate the systematic properties of errors and coverage distribution. But their relationships are not very clear. It is better to add more clear explanations.

*- We added a reference to a review article explaining the properties of the k-mer spectrum to not increase the length of the manuscript even further with a detailed explanation.*

*Moreover, why only 51-mer spectrum is used? As it is very important, more results with various k parameter settings should be added.*

*- We added plots with k=31 and k=71 to the Supplementary and showed that the conclusions hold independent of the particular choice of k.*

5. It is very important for short read simulators to demonstrate their ability of cross-species simulations. The authors give the comparisons of ReSeq and other simulators in this aspect (Figure 7). However, only the comparisons on k-mer spectrum. I think this is not enough, other statistics used in Results section should be also added to make a more comprehensive benchmark.

*- We extended the evaluation of cross-species simulations with the missing statistics.*

6. The speed of simulation is also important, especially when large-scale simulation datasets are needed. The authors give the comparison of speed as well as memory usage (Figure 8), and claimed that ReSeq has effective parallelization, so that it has lower time cost (wall-time). This is a very good feature, however, I also noticed that the memory footprint of ReSeq is quite high in simulation, even an order of magnitude higher than some tools, such as ART and pIRS. So, I would like to know what will happen if all the tools are run in a multi-process way instead of multi-threading, say, the tools are run in a single thread, and many entities are run simultaneously. Since the memory footprints of some simulators are lower, I think a machine can afford many entities of them, and the total throughput could be higher than that of multi-threading mode.

*- We added comparisons with multiple instances of ART and NEAT to the Figure.*

*- BEAR has a very high memory consumption and does part of its training in the simulation step. Thus, it is not clear how to use multiple instances in a beneficial way.*

**Second round of review**

**Reviewer 2**

My comments have been satisfactorily addressed.

**Reviewer 3**

The authors have supplied new results and contents to address my raised concerns.
